# Comparative physiological, biochemical, metabolomic, and transcriptomic analyses reveal the formation mechanism of heartwood for *Acacia melanoxylon*

**DOI:** 10.1186/s12870-024-04884-1

**Published:** 2024-04-22

**Authors:** Ruping Zhang, Zhiwei Zhang, Caizhen Yan, Zhaoli Chen, Xiangyang Li, Bingshan Zeng, Bing Hu

**Affiliations:** 1 Key Laboratory of State Forestry Administration on Tropical Forestry, Research Institute of Tropical Forestry, Chinese Academy of Forestry, Guangzhou, 510520 China; 2 Sihui fengfu forestry development co., ltd, Sihui, 526299 China

**Keywords:** Heartwood formation, Metabolomic, Transcriptomic, Flavonoids, Phenolics, Terpenoids, *Acacia melanoxylon*

## Abstract

*Acacia melanoxylon* is well known as a valuable commercial tree species owing to its high-quality heartwood (HW) products. However, the metabolism and regulatory mechanism of heartwood during wood development remain largely unclear. In this study, both microscopic observation and content determination proved that total amount of starches decreased and phenolics and flavonoids increased gradually from sapwood (SW) to HW. We also obtained the metabolite profiles of 10 metabolites related to phenolics and flavonoids during HW formation by metabolomics. Additionally, we collected a comprehensive overview of genes associated with the biosynthesis of sugars, terpenoids, phenolics, and flavonoids using RNA-seq. A total of ninety-one genes related to HW formation were identified. The transcripts related to plant hormones, programmed cell death (PCD), and dehydration were increased in transition zone (TZ) than in SW. The results of RT-PCR showed that the relative expression level of genes and transcription factors was also high in the TZ, regardless of the horizontal or vertical direction of the trunk. Therefore, the HW formation took place in the TZ for *A. melanoxylon* from molecular level, and potentially connected to plant hormones, PCD, and cell dehydration. Besides, the increased expression of sugar and terpenoid biosynthesis-related genes in TZ further confirmed the close connection between terpenoid biosynthesis and carbohydrate metabolites of *A. melanoxylon*. Furthermore, the integrated analysis of metabolism data and RNA-seq data showed the key transcription factors (TFs) regulating flavonoids and phenolics accumulation in HW, including negative correlation TFs (WRKY, MYB) and positive correlation TFs (AP2, bZIP, CBF, PB1, and TCP). And, the genes and metabolites from phenylpropanoid and flavonoid metabolism and biosynthesis were up-regulated and largely accumulated in TZ and HW, respectively. The findings of this research provide a basis for comprehending the buildup of metabolites and the molecular regulatory processes of HW formation in *A. melanoxylon*.

**Supplementary Information:**

The online version contains supplementary material available at 10.1186/s12870-024-04884-1.

## Introduction

*Acacia melanoxylon*R.Br. (or Blackwood) is a precious tree species owing to its high-quality timber and decay resistance when used in harsh environments [1]. It was often used in shelterbelts, habitats, paper pulp, barren hills afforestation, landscaping, and plywood, and its heartwood (HW) was widely regarded as a necessary material for high-quality furniture, flooring, and musical instruments [2]. The commercial quality and value of the wood depend largely on the characteristics of the HW [3]. For example, most of the trade in high-quality furniture of *Acacia melanoxylon* is also based on the selection of high-grades heartwood [1]. *A. melanoxylon*has been known to form heartwood at an early age [4], and we have a wide selection of clones that are more than a decade old and have developed heartwood. As such, it is important to gain a better understanding of the HW formation in *A. melanoxylon* in order to improve the effective yield and economic value of this valuable material.

The timber of some trees can be divided into sapwood (SW) and HW from the cross-section, except bark. There is usually a transition zone (TZ) at the intersection of SW and HW with 1–3 annual rings wide. HW is traditionally defined as no longer containing living cells, while SW is generally considered to contain a small fraction of living cells [5]. The final stage of wood development in most tree species is HW formation [6]. HW formation is a complex process [7, 8], yet the mechanism of HW formation is still not fully understood. Some studies have shown that HW formation is due to the programmed death of ray parenchyma cells [5, 9–11]. As crucial resin in HW, flavonoids, phenolics, and terpenoids are often the judgment substance of model in HW formation because they are rich in the HW of many trees [6, 7]. Meanwhile, the report that ethylene were related to HW has appeared in early studies. Such as, significantly greater quantities of ethylene were produced during the winter by TZ separating SW from HW than SW during the entire year [12]. Leśniewska found that the significant molecular triggers of tyloses are jasmonates working in tandem with ethylene in HW [13]. JA (jasmonic acid) and SA (salicylic acid) dramatically induced the expression of SaTPS1 (*Santalum album*-terpene synthase) and SaTPS2 in *Santalum album* [14]. The precursor GA24 (gibberellin) could be detected in both SW and TZ of Scots pine [15]. Therefore, it is necessary to pay attention to flavonoid and lignin biosynthesis in HW formation.

Heartwood can be found in both angiosperm and gymnosperm tree species [6]. Heartwood can also be generally considered as the active region of extractives, and SW has high moisture content nutrient and sugar transport [16]. Decay resistance and durability of *A. melanoxylon* are due to the extractives accumulated in the HW region [17–20]. The formation of heartwood is marked by the accumulation of extractives [8]. When trees are subjected to biotic or abiotic stress, several HW extractives are generated in live tissues, contributing to the active defense of living tissues [21]. Phenolics and flavonoids are the two significant extractives in HW [22–24]. Moreover, recent studies have found that phenolics and flavonoids also have a great influence on the progress of HW formation [6, 23, 25]. Wounding or physical injury can also accumulate HW extracts in *Aquilaria sinensis* [26]. HW formation by wounding could activate antioxidative enzyme defense systems, such as superoxide dismutase (SOD) and peroxidase (POD) [26–28]. The activity level of polyphenoloxidase (PPO) has recently also been found to be higher in TZ [29].

Studies have shown that the biosynthesis of secondary metabolites in HW is related to gene expression [30, 31]. The flavonoids and phenolics content in TZ are closely related to the gene expression of phenylalanine ammonia-lyase (PAL), flavanone 3’-hydroxylase (F3’H), chalcone isomerase (CHI), and others [31–33]. Besides, recently, terpenoids accumulated in HW have been proven to be closely related to many up-regulated genes and transcription factors (TF) in TZ [8, 29]. Transcription factors have also been proven to be related to the expression of several key transcription factors (MYB, bHLH, and WRKY) in phenylpropanoid and flavonoid metabolism in *Phoebe zhennan* [34]. In production practice, we can use the regulation mechanism of genes and TFs for metabolites to select and synthesize key extracts from HW. Therefore, the relationship between genes and metabolites and between transcription factors and metabolites for the HW formation of *A. melanoxylon* is also worth discussing. In addition, although early study reported that the position of HW began formed was in TZ using gene expression from the horizontal distribution of trunk (SW, TZ) [6]. There are few reports about the expression of heartwood-related genes in the vertical direction of the trunk at different tree heights. Therefore, it is necessary to study the genes related to HW formation from the vertical direction of the trunk.

However, there is less comprehensive and in-depth evidence on the regulation mechanisms of HW formation for *A. melanoxylon*. Besides, it is still unclear whether HW extractives of *A. melanoxylon* are synthesized in the cells of TZ between SW and HW or transported from the SW. Here, we hypothesized that the expression of gene and TFs was consistent in different tree height positions and horizontal distribution (SW, TZ). The expression of gene and TFs had a positive effect on the HW extracts (flavonoids, phenolics, and terpenes), which were related to the transformation of compounds from SW and TZ and contributed to the formation of HW. In this study, the genes and metabolites involved in the HW formation of *A. melanoxylon* were inferred by integrating physiological, biochemical, transcriptional, and metabonomic analysis. Candidate genes and metabolites related to HW formation of *A. melanoxylon* were initially revealed. Our work provides new insight into the HW formation of *A. melanoxylon* and may contribute to improving HW yield for molecular breeding of *A. melanoxylon*.

## Results

### Histological differences between SW, TZ, and HW of *A. melanoxylon*

**Fig. 1:**
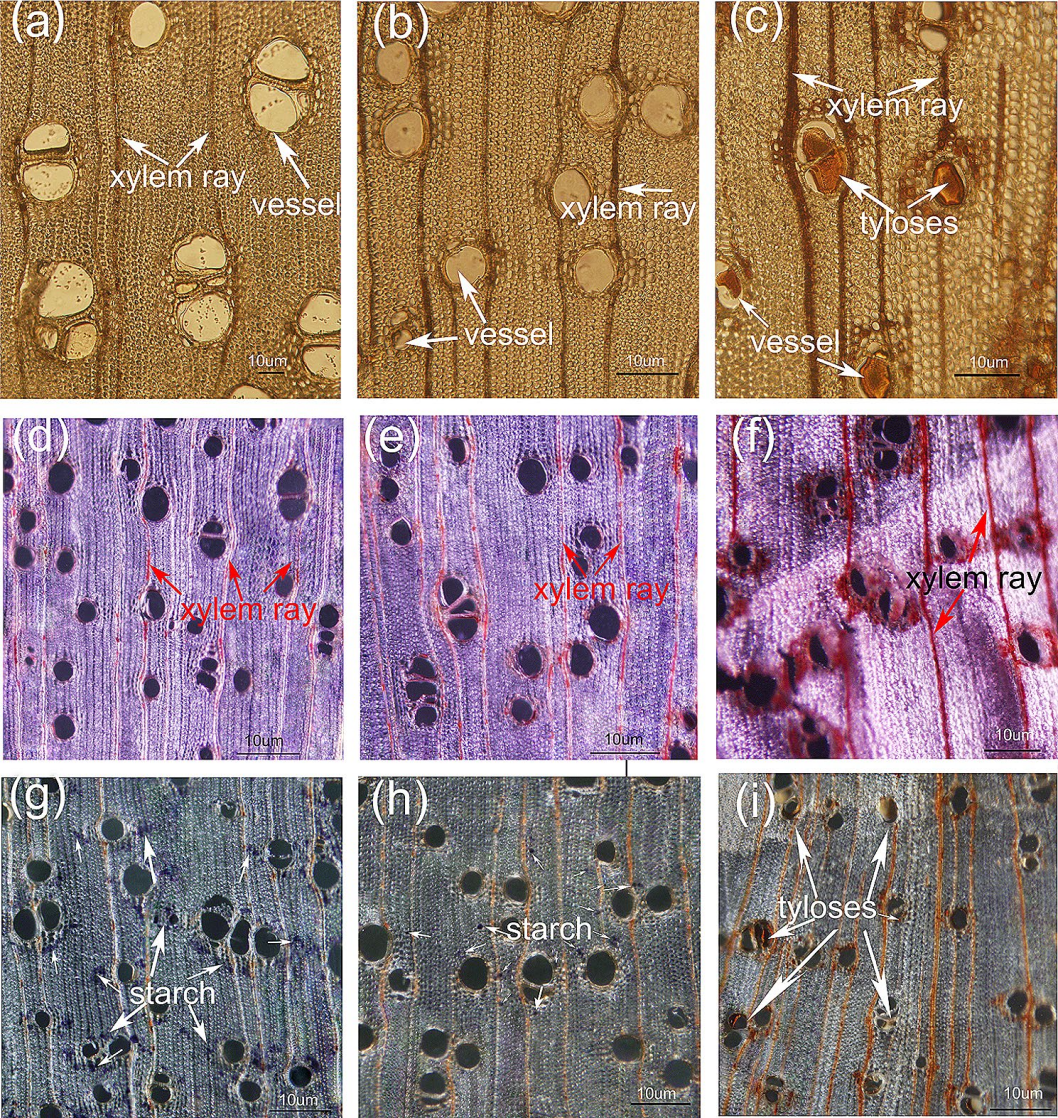
Effect of HW formation on non-structural carbohydrates in *A. melanoxylon*. (**a**, **b**, **c**) As a control group without staining, (**d**, **e**, **f**) PAS staining showed phenols, (**g**, **h**, **i**) I_2_-KI staining showed the remaining starches. Meanwhile, Figures a, d and e are SW. Figures b, e and h are transition areas. In addition, Figures c, f and i are HW. Note: Data are presented in the mean ± SE. Different capital letters indicate that the treatment effect is significantly different at the *p* < 0.05 level. Scale bars = 10 μm

Upon visual inspection, the HW had a reddish brown to brown color, while the SW was seen to be a yellowish white (Figure S1). The TZ was between SW and HW. The borders between SW and TZ, TZ and HW, were usually straightforward to discern and separate. The results of microscopic observation on the cross-section of these three parts showed that most of the vessels were blocked in the HW by the tyloses (Fig. 1c, f, and i). In contrast, little or no tyloses were observed in the vessels from the SW and TZ of *A. melanoxylon* (Fig. 1a, b). Results of PAS (periodic acid-Schiff) staining showed the presence of phenols in ray parenchyma cells, with the accumulation of extractives in HW being greater than that in SW and TZ (Fig. 1d, e, f). The I_2_-KI staining is known to stain starches blue. Our staining results revealed that starches were primarily localized in SW and TZ of *A. melanoxylon*, and the amount of starches decreased from SW to TZ (Fig. 1g, h). Notably, starches were absent in the HW (Fig. 1i). Interestingly, a considerable amount of starches were observed close to the ray parenchyma cells in SW and TZ (Fig. 1g, h).

### The contents of critical substances and endogenous hormones in SW, TZ, and HW of *A. melanoxylon*

**Fig. 2:**
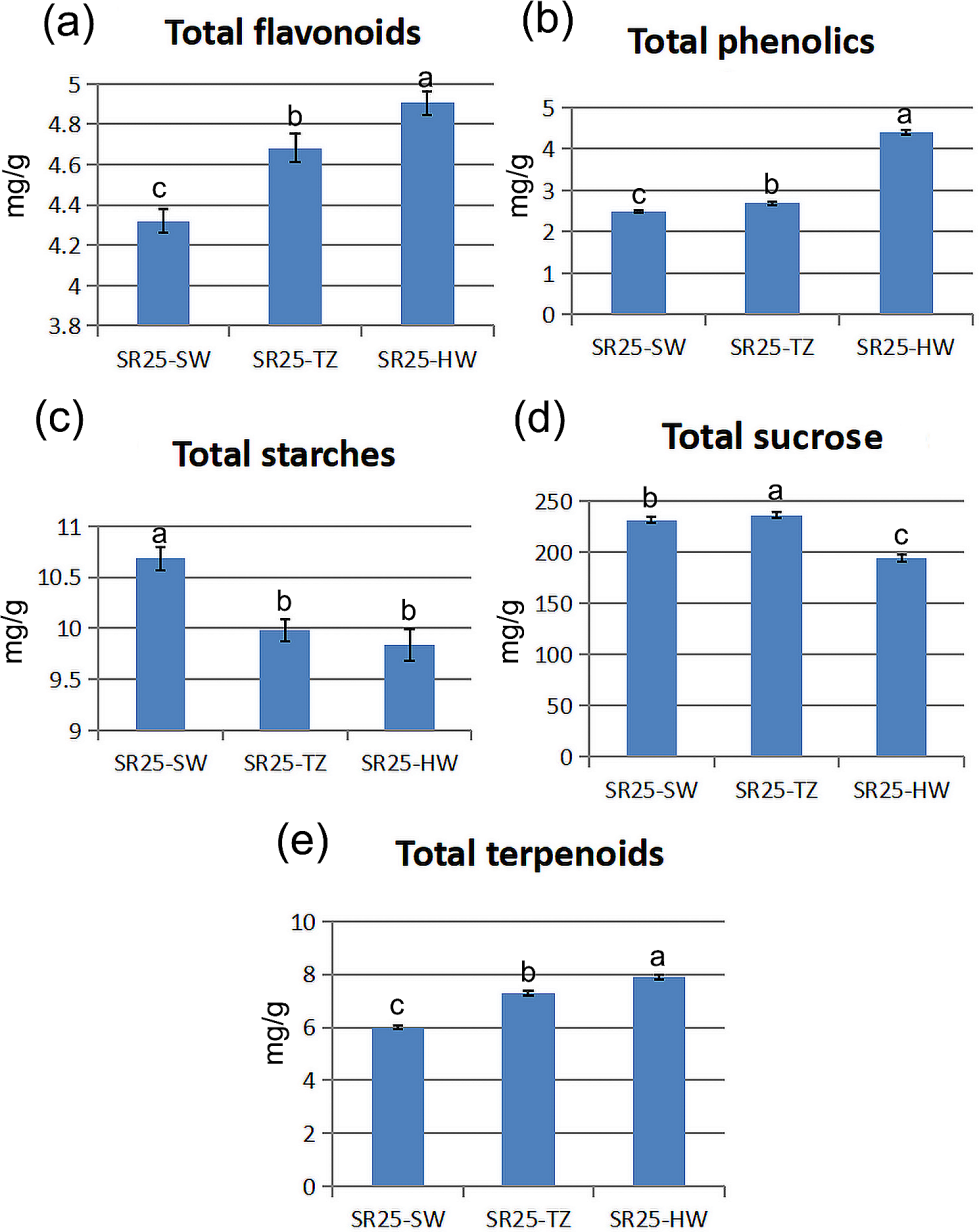
The content comparison of total flavonoids, phenolics, starches, sugars, and terpenoids in SW, TZ, and HW of *A. melanoxylon*. Note: Data are presented in the mean ± SE. Different capital letters indicate that the treatment effect is significantly different at the *p* < 0.05 level

The xylem contains a high concentration of starches, sugars, phenolics, and terpenes, which we have determined. Our results showed a gradual increase in the content of flavonoids, terpenoids, and phenolics in SW, TZ, and HW (Fig. 2a, b, e, Table S1). In contrast, starch contents decreased significantly in TZ and HW compared to SW (Fig. 2c, Table S1). The concentration of soluble sugar in TZ was substantially higher than that of SW, yet significantly lower in HW (Fig. 2d, Table S1). The results of this determination largely mirror the findings of the histological observation.

The results of our hormone determination showed that the content of gibberellin (GA), jasmonic acid (JA), salicylic acid (SA), and cytokinin (CTK) in TZ were all significantly higher than in SW and HW, and the levels of ethene (ETH) were notably higher in TZ and HW compared to SW (Table 1, Table S3). The amount of auxin (IAA), which is responsible for controlling plant growth, decreased in TZ. For all hormone contents, the concentrations of IAA and CTK were notably higher than the other hormones, and the contents of GA, IAA, SA, CTK, and ETH were found to be higher in HW than in SW (Table 1, Table S3).

**Table 1:** Differences of endogenous hormone content in SW, TZ and HW of *A. melanoxylon*

parameter	SR25-SW	SR25-TZ	SR25-HW
GA(µg/L)	0.51(0.02)c	0.60(0.03)a	0.55(0.01)b
JA(µg/L)	0.26(0.01)b	0.29(0.01)a	0.25(0.01)b
IAA(µg/L)	82.79(2.25)b	81.02(3.96)b	96.76(1.50)a
SA(µg/L)	0.19(0)c	0.23(0)a	0.21(0.01)b
CTK(µg/L)	53(2.80)b	58.47(1.05)a	55.28(2.46)b
ETH(µg/L)	0.41(0.01)b	0.44(0.01)a	0.43(0.01)a

Note: Data are presented in the mean ± SE. Different capital letters indicate that the treatment effect is significantly different at the *p* < 0.05 level

### Enzymatic activity related to critical substances biosynthesis pathways in SW, TZ, and HW of *A. melanoxylon*

**Fig. 3:**
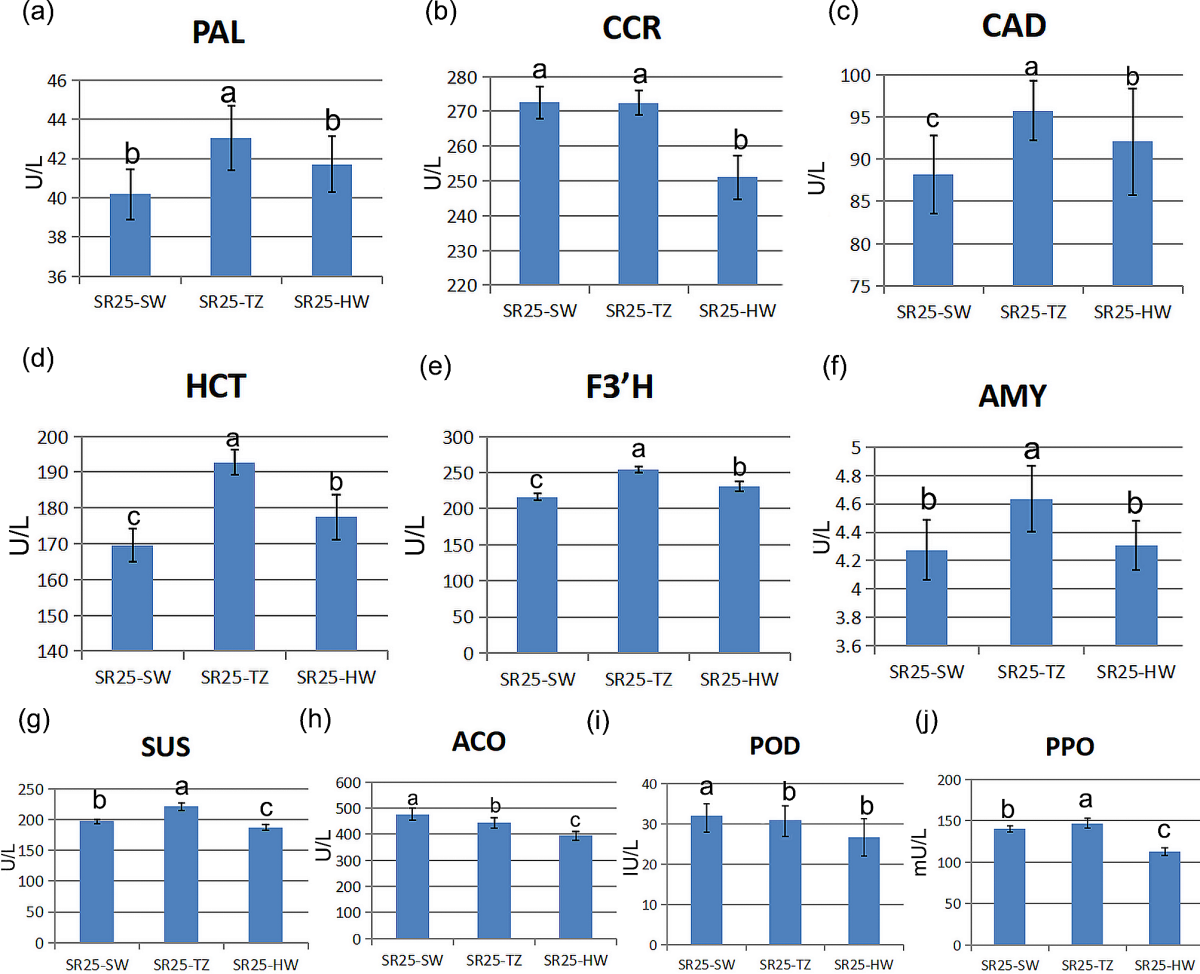
Enzymatic activity of SW, TZ and HW of *A. melanoxylon*. Note, POD: Peroxidase; PPO: Polyphenol Oxidase; ACO: 1-aminocyclopropane-1-carboxylate oxidase; HCT: hydroxycinnamoyl shikimate transferase; F3’H: flavanone 3’-hydroxylase; CAD: cinnamyl-alcohol dehydrogenase; PAL: Phe ammonia lyase; CCR: cinnamoyl-CoA reductase; AMY: alpha-amylase; SuSy: sucrose synthase

Results from enzyme activity tests showed that TZ had significantly higher PAL (phenylalanine ammonia lyase) and CAD (cinnamyl-alcohol dehydrogenase) enzyme activities compared to SW, as demonstrated in Fig. 3a, b, c and Table S2. Alpha-amylase (AMY) and SuSy (sucrose synthase) are significant enzymes in the starch and sucrose metabolism pathway, and their activity is higher in TZ than in HW, as evidenced by Fig. 3f and Table S2. The activity of SuSy was also higher in SW than in HW (Fig. 3g, Table S2). The 1-aminocyclopropane-1-carboxylate oxidase (ACO), which is responsible for the synthesis of ethylene precursors, had a greater enzyme activity in SW and TZ than HW, based on Fig. 3h and Table S2. Additionally, POD and PPO activities were greater in SW compared to HW, with TZ showing a high concentration level (Fig. 3i, j, Table S2).

### Metabonomic signatures of SW, TZ, and HW of *A. melanoxylon*

**Fig. 4:**
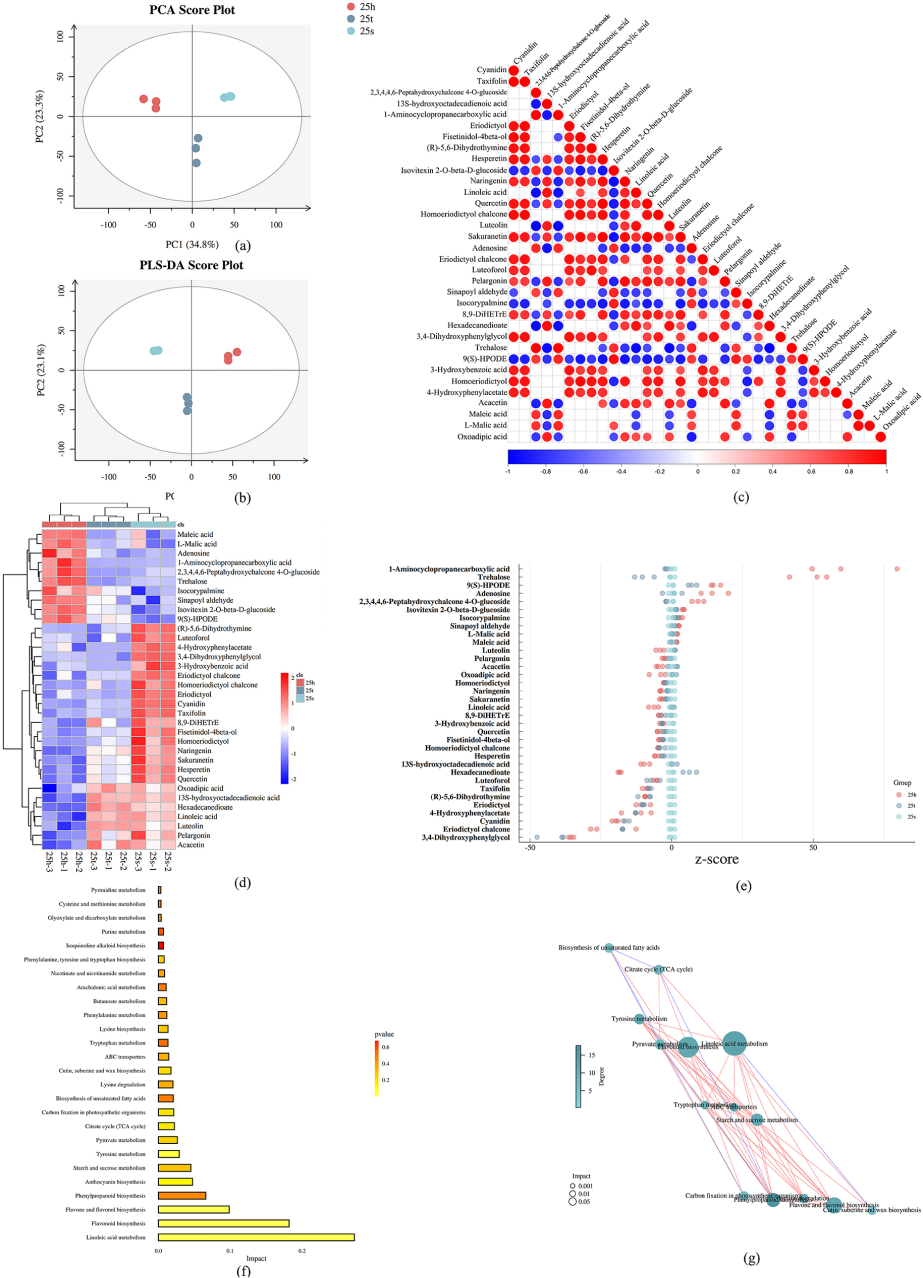
Preliminary analysis of metabonomic data. (**a**–**b**) Principal component analysis of metabolites for SW, TZ, and HW (**c**) Correlation analysis for differential metabolites among SW, TZ, and HW. (**d**) Enrichment analysis of differential metabolites among SW, TZ, and HW (**e**) Z-score (standard score) analysis of 34 DEMs (**f**) Histogram analysis of KEGG metabolic pathway (**g**) Network diagram analysis of pathway correlation of differential metabolites

In order to understand the difference in metabolites between SW, TZ, and HW in *A. melanoxylon* from qualitative and quantitative perspectives, metabolomic profiling was performed based on the UPLC/HR-MS system. The 9 samples of xylem (SW, TZ, and HW) were separated into three groups by both PLS-DA (partial least squares-discriminate analysis) and PCA (principal components analysis). PC1 and PC2 explained that the difference between intra-group is greater than inter-group, and there is a slight difference within the SW (Fig. 4a, b). A total of 4595 differentially accumulated metabolites (DAM) were identified in SW, TZ, and HW. Among these metabolites, 761 up-regulated and 2314 down-regulated DAMs were identified in SW and TZ, 2101 up-regulated and 2271 down-regulated DAMs were identified in HW and SW, and 2504 up-regulated and 1186 down-regulated DAMs were identified in HW and TZ (Table S4). Based on a *p*-value < 0.05 and VIP ≥ 1, DAMs were also identified from different groups (HW vs. SW, TZ vs. SW, HW vs. TZ, HW vs. TZ vs. SW) (Figure S2).

### DEMs between SW, TZ, and HW primarily related to phenylpropanoid and flavonoid biosynthesis, starch and sucrose metabolism

A comprehensive analysis of the differentially enriched metabolites (DEMs) in SW, TZ, and HW groups revealed 34 DEMs, the results of which were represented in a hierarchical clustering (Figure S2). We then conducted a correlation analysis of the DEMs and discovered that acacetin, maleic acid, l-malic acid, and oxoadipic acid had a significant positive correlation with other metabolites (Fig. 4c). In contrast, cyanidin, taxifolin, 2,3,4,4,6-pentahydroxychalcone-4-o-glucoside, and 13s-hydroxyoctadecadienoic acid were significantly inversely associated with other metabolites (Fig. 4c). We also found that the relative contents of ten DEMs (maleic acid, l-malic acid, adenosine, 1-aminocyclopropanecarb oxylic acid, 2,3,4,4,6-peptahydroxychalcone-4-o-glucoside, trehalose, Isocorypalmine, sinapoyl aldehyde, isovitexin 2-o-beta-d-glucoside, 9(S)-HPODE) in HW were significantly higher than those in SW using cluster thermogram and violin diagram analysis (VIP ≥ 1, *p*-value < 0.05, FoldChange > 2) (Fig. 4d, S3). Therefore, the ten DEMs from flavonoid biosynthesis (maleic acid, l-malic acid, adenosine, 1-aminocyclopropanecarboxylic acid, 2,3,4,4,6-peptahydroxychalcone-4-o-glucoside), starch and sucrose metabolism (trehalose), flavone and flavonol biosynthesis (Isocorypalmine, sinapoyl aldehyde, isovitexin 2-o-beta-d-glucoside), and linoleic acid metabolism [9(S)-HPODE] were chosen as potential metabolites that may related to HW formation of *A. melanoxylon*. Besides, to further explore the DEMs related to HW formation, the Z-score (standard score) was used to measure the relative content of 34 metabolites (Fig. 4e). The results showed that four group of metabolites with a high accumulation in HW, including 1-aminocyclopropanecarboxylic acid, trehalose, 9(S)-HPODE, and adenosine. Then, the 34 metabolites were annotated through the Kyoto Encyclopedia of Genes and Genomes (KEGG) database to determine the metabolic pathway of DEMs. Our results revealed that linoleic acid metabolism, flavonoid biosynthesis, phenylpropanoid biosynthesis, flavone and flavanol biosynthesis, and starch and sucrose metabolism were the main significant enriched pathways (Fig. 4f, Table S5). We conducted a correlation analysis and found that phenylpropanoid and flavonoid biosynthesis, starch and sucrose metabolism, were significantly correlated (Fig. 4g).

### Transcriptome signatures of SW, TZ, and HW of *A. melanoxylon*

After quality control of sequencing data for each sample, at least 6.56 Gb of clean data were produced, with a minimum quality score of Q30 of 92.02%. Each library’s mapping ratio ranged from 86.22 to 89.90%.

**Fig. 5:**
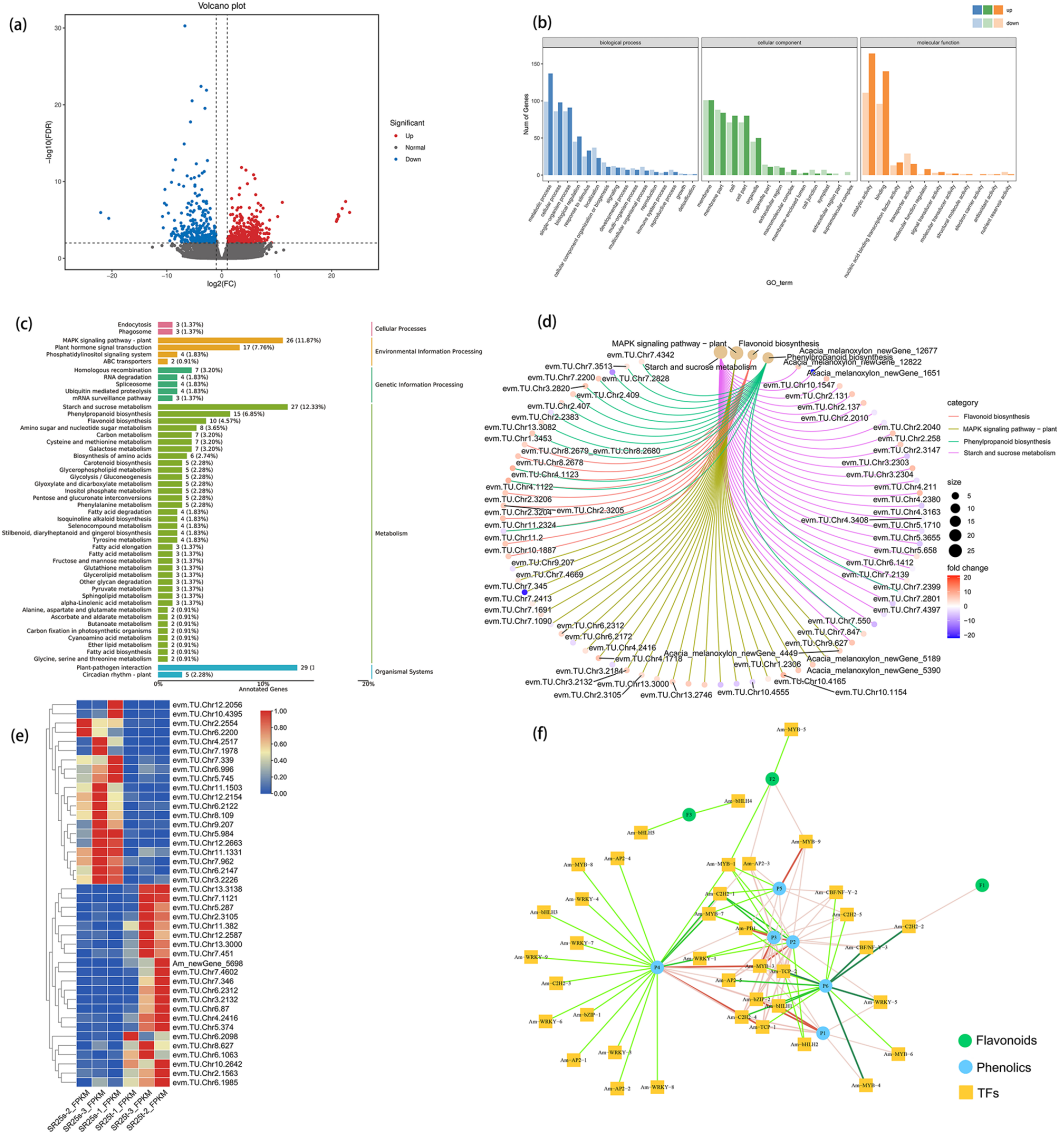
Transcriptomic analysis of *A. melanoxylon*. (**a**) Volcano plots of DEGs of *A. melanoxylon*. (**b**) GO enrichment analysis of DEGs. (**c**-**d**) The KEGG classification and enrichment analysis of DEGs (**e**) Cluster thermogram of differentially expressed transcription factors (**f**) Correlation network diagram between differentially expressed transcription factors and differentially enriched phenolics and flavonoids

A total of 700 transcripts had significant differential expression between TZ and SW, 396 were up-regulated, and 304 were down-regulated (Table S6, Fig. 5a). The results of Gene ontology (GO) analysis indicated that “metabolic process” and “cellular process” were significantly overrepresented in the biological process, “catalytic activity” and “binding” were predominant in the molecular function, “membrane” and “membrane part” was principal in the cellular components (Fig. 5b). The results of KEGG enrichment indicated that the MAPK signaling pathway, starch and sucrose metabolism, phenylpropanoid biosynthesis, and flavonoid biosynthesis were significantly overrepresented (Fig. 5c, d). In addition to the three KEGG pathways, we also explored flavone and flavonol biosynthesis as well as phenylalanine metabolism.

### The phenylpropanoid and flavonoid pathways are implicated in the HW formation of *A. melanoxylon*

**Fig. 6:**
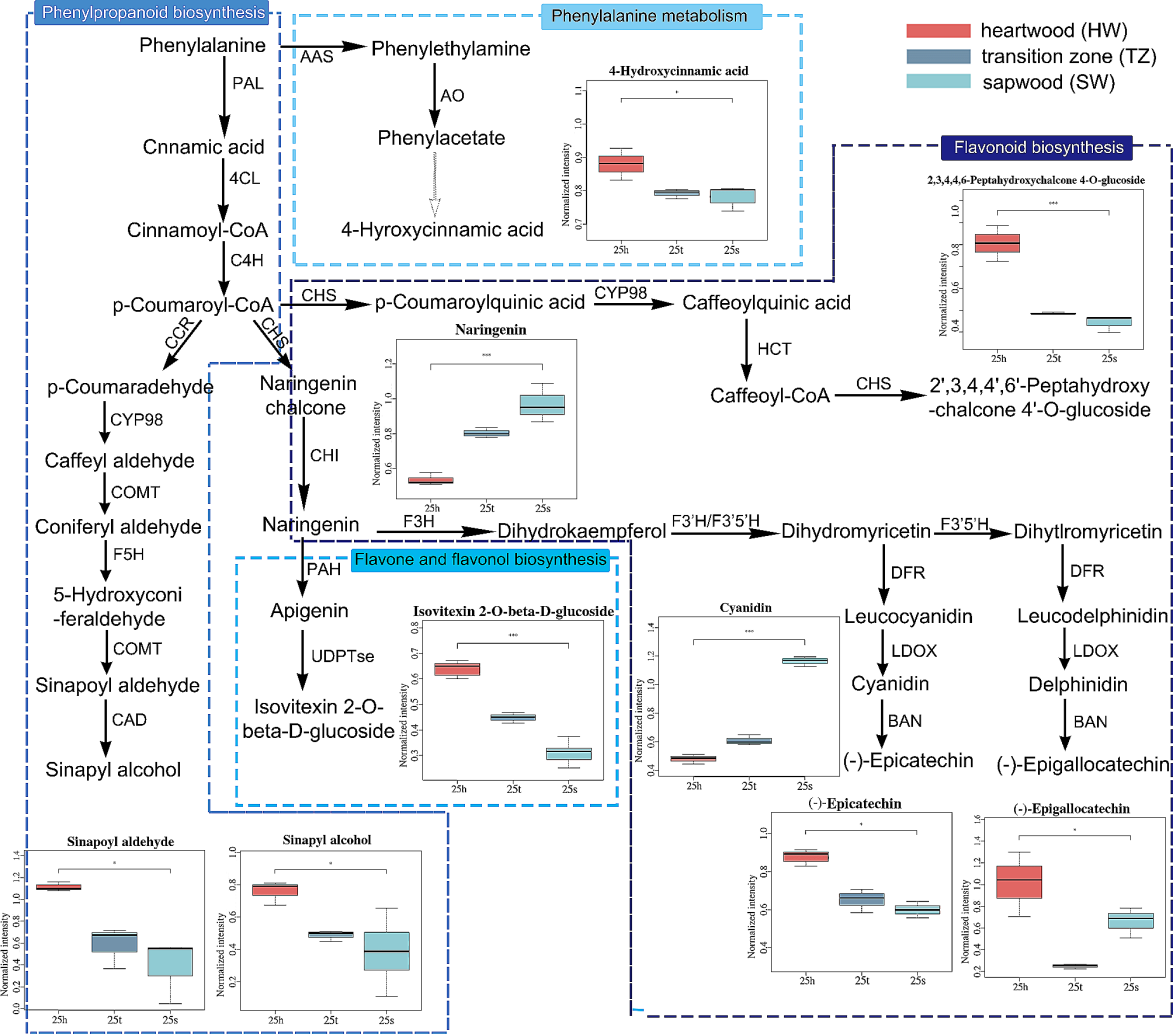
Analysis of differentially enriched metabolites in four pathways related to flavonoids and phenylpropanoids Note, POD: Peroxidase; PPO: Polyphenol Oxidase; ACO: 1-aminocyclopropane-1-carboxylate oxidas; HCT: hydroxycinnamoyl shikimate transferase; F3’H: flavanone 3’-hydroxylase; CAD: cinnamyl-alcohol dehydrogenase; PAL: Phe ammonia lyase; CCR: cinnamoyl-CoA reductase; AMY: alpha-amylase; SuSy: sucrose synthase

The DEMs identified (Fig. 4) mainly come from four pathways, comprising phenylpropanoid metabolism and biosynthesis, flavonoid biosynthesis, flavone, and flavanol biosynthesis. The results of DEGs related to the extracts of HW formation were enriched into several pathways, which mainly involved phenylpropanoid biosynthesis, flavonoid biosynthesis, and flavone and flavanol biosynthesis. Consequently, we created regulatory maps of genes and metabolites with regard to the notable DEMs locations of these five pathways, including the phenylalanine metabolism pathway (Fig. 6 and Figure S4). According to Fig. 6, the metabolites of four pathways varied significantly depending on the radial timber location (SW, TZ, and HW). The concentrations of phenylpropanoid and flavonoid derivatives were notably higher in the TZ and HW than in the SW. For example, the (-)-epicatechin, (-)-epigallocatechin, isovitexin 2-o-beta-d-glucoside, 1-aminocyclopropanecarboxylic acid, sinapoyl aldehyde, 4-hydroxycinnamic acid, sinapoyl aldehyde, and 2,3,4,4,6-Peptahydroxychalcone 4-o-glucoside were significantly higher in HW than in SW and TZ. On the contrary, the accumulation of naringenin and cyanidin in SW was significantly higher than in the TZ and HW (Fig. 6). Naringenin and cyanidin are located upstream of isovitexin 2-o-beta-d-glucoside, (-)-epicatechin, and (-)-epigallocatechin, and their high concentration in SW may be associated with the high concentration of metabolites in HW.

We further examined the gene expression levels that were associated with the four pathways. The gene expression regulatory map is presented in Figure S4. The up-regulation of PAL (evm.TU.Chr2.407, evm.TU.Chr2.409), AAS (aromatic-L-amino-acid decarboxylase) (AM_newGene_1266), AO (primary-amine oxidase) (evm.TU.Chr6.29), COMT (caffeic acid 3-o-methyltransferase) (evm.TU.Chr13.3082), and CAD (evm.TU.Chr1.3453) genes in TZ may contributed to biosynthesis of phenylpropanoid. Up-regulated expression of CHS (chalcone synthase) (evm.TU.Chr8.2679_2680, evm.TU.Chr8.2678), CHI (evm.TU.Chr10.1887), HCT (evm.TU.Chr4.1122, evm.TU.Chr4.1123, evm.TU.Chr11.2324), and F3’H (evm.TU.Chr11.2) genes in TZ may contributed to flavonoids biosynthesis (Fig. 6B). We also found one CCR (evm.TU.Chr2.2383) gene was significantly up-regulated in the SW (Figure S4). The most up-regulated expression patterns of transcripts in the TZ indicated the initial formation of HW.

### The sugars and terpenoids pathways were activated in TZ in the HW formation of *A. melanoxylon*

**Fig. 7:**
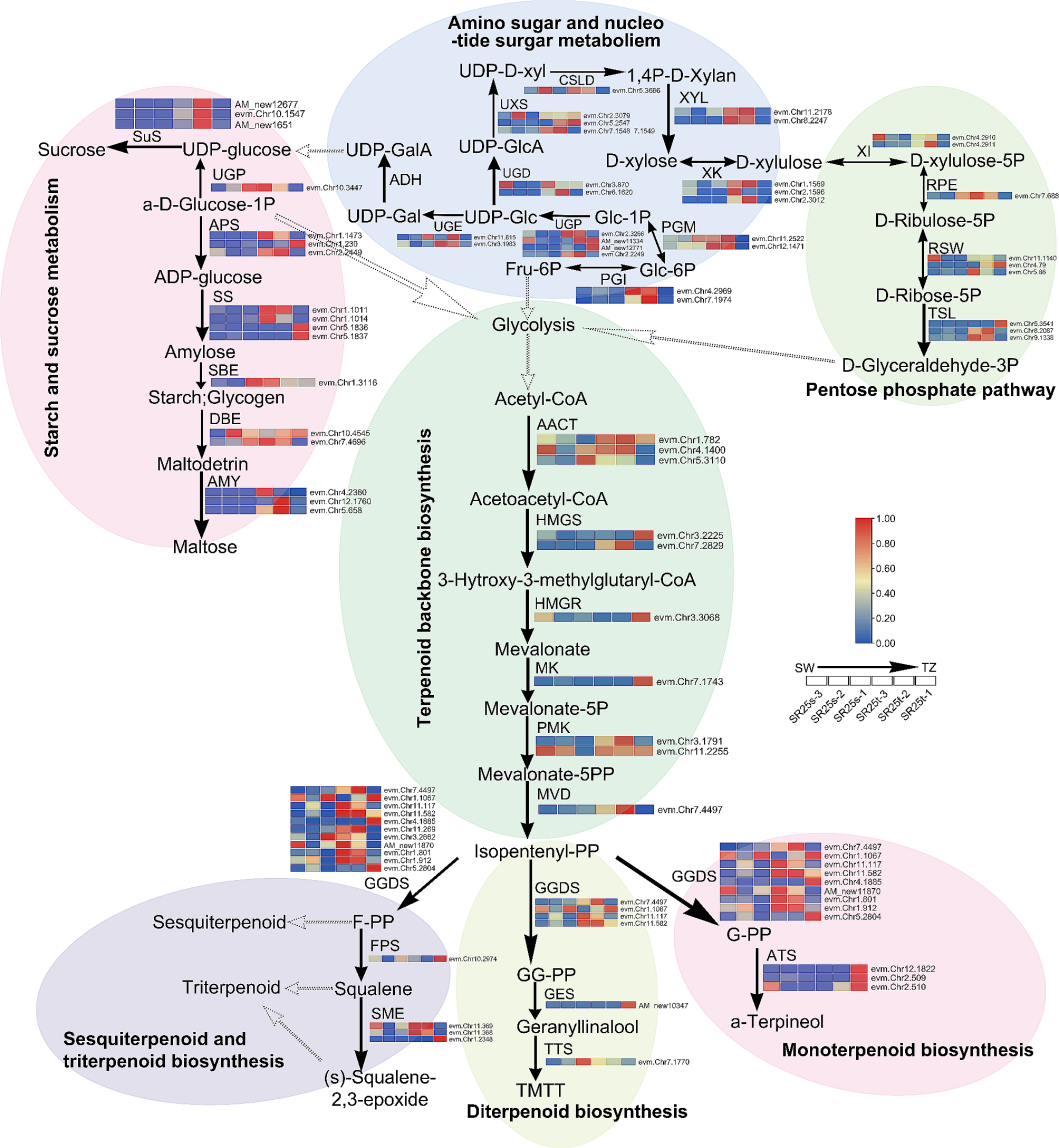
Schematic diagram of different pathways activated in *A. melanoxylon*. Note, SuSy: sucrose synthase; UGP: UTP–glucose-1-phosphate uridylyltransferase; APS: ADP-glucose; SS: starch synthase; SBE: starch branching enzyme/1,4-alpha-glucan branching enzyme; DBE: debranching enzyme 1/isoamylase; AMY: alpha-amylase; ADH: alcohol dehydrogenase; UGE: UDP-glucose 4-epimerase; UGD: UDP-glucose dehydrogenase; UXS: UDP-XYL synthase; CSLD: cellulose synthase-like D5; XYL: xylan 1,4-beta-xylosidase/Glycosyl hydrolase; XK: xylulose kinase-2/ xylulokinase; XI: xylose isomerase, RPE: ribulose-phosphate 3-epimerase; RSW: ribose 5-phosphate isomerase A; TSL: transketolase; PGM: Phosphoglucomutase/phosphomannomutase; PGI: phosphoglucose isomerase 1/ glucose-6-phosphate isomerase; AACT: acetyl-CoA C-acetyltransferase; HMGS: hydroxymethylglutaryl-CoA synthase, HMGR: hydroxymethylglutaryl-CoA reductase; MK: mevalonate kinase; PMK: phosphomevalonate kinase; MVD: diphosphomevalonate decarboxylase; GGDS: geranylgeranyl diphosphate synthase, type III; FPS: farnesyl-diphosphate; SME: squalene monooxygenase; GES: geranylgeranyl diphosphate synthase, type III; TTS: trimethyltridecatetraene/dimethylnonatriene synthase; ATS: (-)-alpha-terpineol synthase

In Fig. 7, the biosynthesis of terpenoids (monoterpenoid, diterpenoid, sesquiterpenoid, and triterpenoid) were initiated by the conversion of isopentenyl-PP, catalyzed by the enzyme geranylgeranyl diphosphate synthase (GGDS). Farnesyl-diphosphate (FPS) converts the activated famesyl-PP (F-PP) into Squalene, and then it was converted into (s)-Squalene-2,3-epoxide under the catalysis of squalene monooxygenase (SME). Geranyllinalool synthase (GES) converted the activated geranylgeranyl-PP (GGPP) into geranyllinalool, and then trimethyltridecatetraene synthase (TTS) converted the geranyllinalool into (E, E)-4,8,12-trimethyltrideca-1,3,7,11-tetraene (TMTT). (-)-alpha-terpineol synthase (ATS) converted the activated geranyl-PP (G-PP) into a-Terpineol. It was determined that TZ was the expressed zone in which GES-encoding transcripts were most abundant, being 17 times more than the other zones (Fig. 7 and Table S7). GGDS-encoding transcripts were 1-3-fold up-regulated expression in the TZ. Congruently, 1–9 fold up-regulation of ATS-encoding transcripts and 1–12 fold up-regulation of SME-encoding transcripts were also observed in TZ than in SW. Upstream of three terpenoid biosynthesis was the terpenoid backbone biosynthesis that provides isopentenyl-PP for terpene synthesis (Fig. 7 and Table S7). The terpenoid backbone biosynthesis started with the conversion of acetyl-CoA to acetoacetyl-CoA by acetyl-CoA c-acetyltransferase (AACT). The following enzyme, hydroxymethylglutaryl-CoA synthase (HMGS), operating on 3-Hydroxy-3-methylglutaryl-CoA, was found to be 2 to 5-fold up-regulated in TZ (Fig. 7 and Table S7). Several transcripts related to terpenoid backbone biosynthesis were also up-regulated with 1- to 3-fold change in TZ than in SW, including hydroxymethylglutaryl-CoA reductase (HMGR), mevalonate kinase (MK), phosphomevalonate kinase (PMK), and diphosphomevalonate decarboxylase (MVD) (Table S7).

Upstream of terpenoid backbone biosynthesis was glycolysis / gluconeogenesis. Further upstream were several sugar metabolism pathways, which provided glycolysis for terpenoid backbone biosynthesis (Fig. 7 and Table S7). Pentose phosphate pathway started with the conversion of d-xylulose-5P to d-Ribulose-5P and d-xylulose by the enzyme ribulose-phosphate 3-epimerase (RPE) and xylose isomerase (XI), respectively. Ribose 5-phosphate isomerase (RSW) converted the d-Ribulose-5P into d-Ribose-5P, and transketolase (TSL) converted the d-Ribose-5p into d-glyceraldehyde-3p that could contribute to glycolysis (Fig. 7 and Table S7). We found transcripts RSW and TSL were up-regulated 1- to 8-fold and 2- to 11-fold in TZ than in SW. We also observed transcripts of xylulose kinase (XK), which were up-regulated 1-280 times in TZ (Fig. 7 and Table S7). XK converted D-xylulose to D-xylose in amino sugar and nucleotide sugar metabolism. On the other hand, D-xylose could also be converted by glucose-1p (Glc-1P) through several enzymes, including those for udp-sugar pyrophosphorylase (UGP), udp-glucose dehydrogenase (UGD), udp-xyl synthase (UXS), and cellulose synthase-like d (CSLD). On the contrary, Glc-1P was converted into fructose 6-phosphate (Fru-6P) by the phosphoglucomutase (PGM) and enzyme phosphoglucose isomerase (PGI), which acted on the downstream glycolysis. Udp-glucose 4-epimerase (UGE) and alcohol dehydrogenase (ADH) converted udp-glc (UDP-Glucose) to udp-glucuronate (UDP-GalA) and acted on the downstream pathway. We also found that ugp-encoding and xyl-encoding transcripts are 1–6 and 1-17-fold in TZ, respectively (Fig. 7 and Table S7). Transcripts encoding sucrose synthase (SuSy) were 28–85 times up-regulated in TZ. SuSy converted udp-glucose to sucrose in starch and sucrose metabolism. The starch synthase (SS) operating on amylose was found to be 3- to 5-fold up-regulated in TZ (Fig. 7 and Table S7). And finally, transcripts for alpha-amylase (AMY) were up-regulated with 18–172 fold change. According to our findings, sugar breakdown was subsequently diverted to downstream processes such as glycolysis/gluconeogenesis, which both built carbon backbone and provided energy for the metabolic process. The transcriptome analysis indicated that terpenoids are produced locally in the TZ.

### Differentially expressed TFs between SW and TZ of *A. melanoxylon*

There were 12 differentially expressed TFs (DETFs) identified. The DETFs were annotated to encode WRKY, MYB, bHLH, C2H2, AP2, bZIP, TCP, CBF-B/NF-YA, C2H2, PB1 families (Fig. 5e and Table S8). The WRKY, MYB, bHLH, C2H2, AP2, and bZIP TFs were up-regulated in TZ compared with those in SW. The TGA4, TCP, CBF-B/NF-YA, C2H2, and PB1 TFs were up-regulated in SW. In the MAPK signaling pathway, transcription factors related to pathogen defense response, cell death, H_2_O_2_ production, and stomatal development were up-regulated in TZ more than in SW (Figure S5). At the same time, we could see that phenylalanine metabolism indirectly leads to the enhancement of disease resistance through TGA in the pathway of plant hormone signal transduction (Figure S6). Besides, A-linolic acid metabolism indirectly affects terpenoid metabolism through JA (Figure S6).

In addition, TFs related to phenylpropanoid and flavonoid synthesis were identified by the analysis of the correlation network (Fig. 5f and Table S7). The results showed that 1 C2H2 transcript positively correlated with flavonoid metabolites (F1 or 4-hydroxycinnamic acid). In contrast, 2 bHLH unigenes negatively correlated with F3 (sinapoyl aldehyde). Besides, 2 TFs genes (MYB) were negative, and 2 TFs genes (1 MYB and 1 AP2.) were positive correlation with F2 (2,3,4,4,6-Peptahydroxychalcone 4-o-glucoside). Moreover, we also found that 9 TFs genes positively correlated with P1 (naringenin). On the other hand, 5 TFs genes were negative, and 15 TFs genes positively correlated with P2 (eriodictyol). 5 TFs genes were negative, and 13 TFs genes positively correlated with P3 (cyanidin). 17 TFs genes were negative, and 9 TFs genes positively correlated with P4 (epicatechin). 3 TFs genes were negative and 7 TFs genes positively correlated with (-)-epigallocatechin (P5). 15 TFs genes had negative correlation with P6 (isovitexin 2-o-beta-d-glucoside). Among these transcription factors, AP2, bZIP, CBF, PB1, and TCP have more transcripts that are positively correlated with phenylpropanoids and flavonoids (Fig. 5f and Table S8). Likewise, WRKY and MYB have more transcripts that are positively correlated with the phenylpropanoids and flavonoids (Fig. 5f and Table S8). Transcripts of C2H2 and bHLH have almost the same positive and negative correlation with phenylpropanoid and flavonoid metabolites (Fig. 5f and Table S8).

### DEGs related to phytohormone, PCD, and dehydration between SW and TZ of *A. melanoxylonbetween*

The plant hormones were found to be related to HW formation. In the study, the sequences encoding ACO (ACC or 1-amino cyclopropane carboxylic acid oxidase) had a 15–116 fold induction in TZ compared to SW. And transcripts responding to jasmonic acid (JA) showed a 16–69 fold change in TZ than in SW (Table S9). Transcripts related to auxin transport and auxin response were 8- 4,324,971 times down-regulated in SW, though sequences involved in auxin biosynthesis were not found in TZ (Table S9). Meanwhile, three transcripts related to SA (Salicylic acid) and three transcripts related to GA (Gibberellin) were up-regulated with 15–48 and 3–58 fold changes in TZ than in SW, respectively (Table S9). Despite the lack of evidence connecting CTK (Cytokinin) to HW formation, four transcripts related to CTK biosynthetic were found to be decreased by 3–23 times in TZ compared to SW.

Wood development begins with the differentiation of the vascular cambium into secondary xylem cells, followed by cell expansion and secondary wall deposition, and finally, programmed cell death and HW formation. Wood senescence in TZ is accompanied by dehydration of ray parenchyma cells. Transcripts encoding cytoskeleton-associated protein (CKAP) were up-regulated in TZ, with an 8 fold change (Table S9). CKAP is involved in PCD of maize. The desiccation-related protein (DRP) is related to the dehydration tolerance of plants. We observed that a DRP transcript was up-regulated 100 times in TZ compared to SW (Table S9). Moreover, a group of aquaporin-like transcripts was 22–395 fold-change down-regulated in TZ than in SW (Table S9).

### Expression patterns of genes related to TFs, phenylpropanoid, flavonoid, and sugars pathways in SW and TZ along the tree height of *A. melanoxylon*

**Fig. 8:**
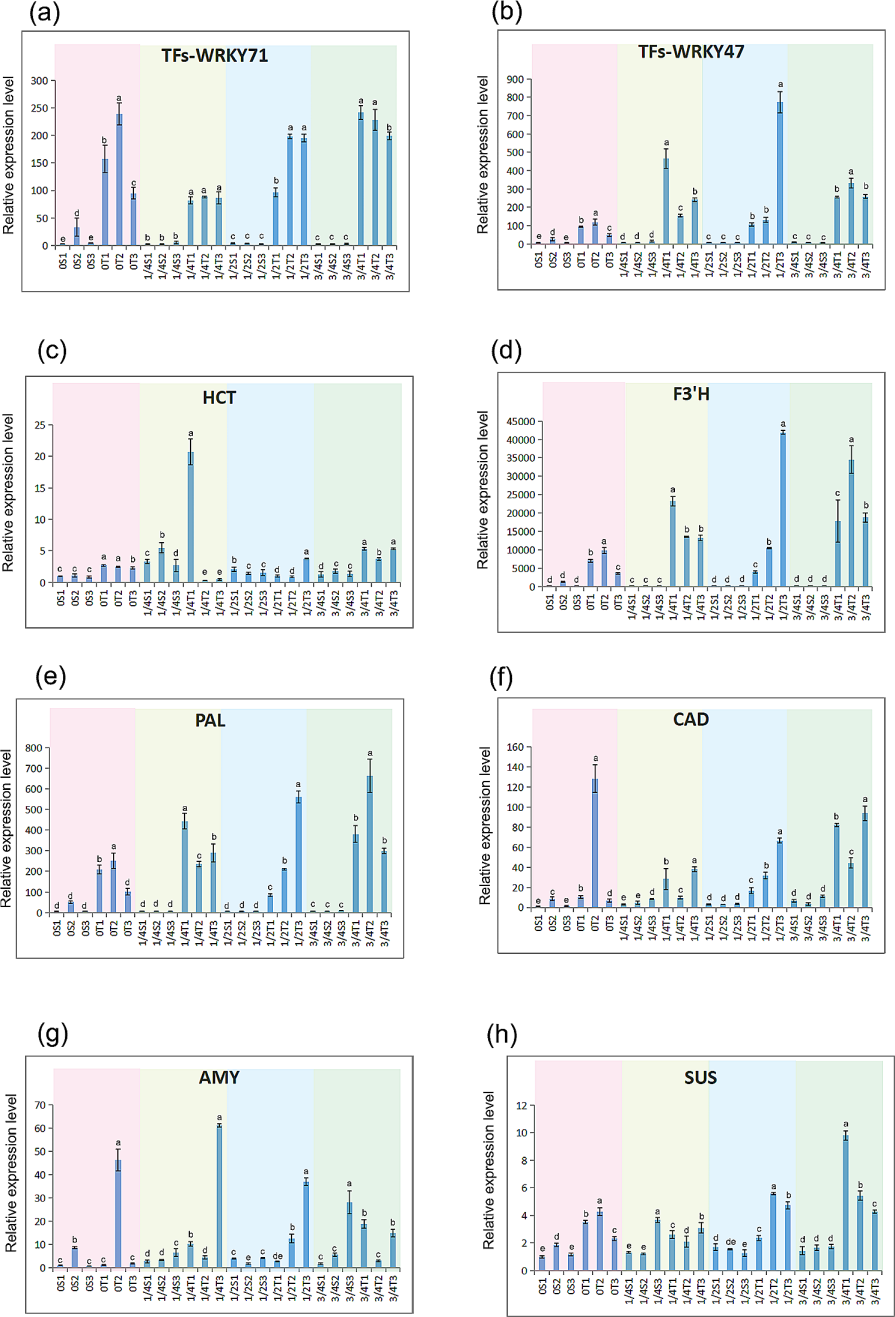
Expression profile of TFs and genes in different tree height/tree ages of *A. melanoxylon*. The expression level of other transcripts is based on the ribosomal protein L4E (RPL4) transcripts

Among 91 DEGs related to sugar, terpenoids, phenolics, and flavonoids, we selected several gene families for quantitative PCR verification (Table S7, S10). The expression of DEGs in SW and TZ at four positions of tree height (ground or base or 0, 1/4, 1/2, and 3/4 total tree height) were detected and analyzed by qRT-PCR. The other transcripts were expressed in relation to the geometric mean of RPL4, as observed in Fig. 8 and Table S10-11. The results of the study indicated a strong connection between FPKM and RT-PCR, verifying that all gene expressions were consistent. For instance, from the FPKM results, the expression level of HCT (evm.TU.Chr4.1122) and F3’H (evm.TU.Chr11.2) was 347 and 76 times higher in TZ than in SW of *A. melanoxylan*, respectively. Moreover, the expression level of PAL (evm.TU.Chr2.409) and CAD (evm.TU.Chr1.3453) were 31 and 64 folds higher in TZ, respectively. This suggested that the majority of the DEGs were up-regulated in TZ at the upstream of the phenylpropanoid and flavonoid biosynthesis pathways. In addition, AMY (evm.TU.Chr4.2380) and SuSy (evm.TU.Chr10.1547) were 172 and 28 folds higher in TZ, respectively. And two WRKY TFs (evm.TU.Chr2.1563, evm.TU.Chr7.346) were found to be 7 and 27 fold changes in TZ compared to SW, respectively. The transcript expression showed an increase in TZ compared to SW, even when the location of the tree height was at the ground/base. And expression levels of 8 unigenes were up-regulated in TZ more than in SW, regardless of the tree ages/tree heights (Fig. 8, Table S11).

From the qRT-PCR results, RNA yields were regular at horizontal direction of the trunk (SW and TZ), but they were not systematic throughout the vertical direction of the trunk (different tree height/age). Expression levels of WRKY71 were increased in the TZ from 1/4 to 3/4 tree height. However, the expression level of WRKY71 was raised in the TZ throughout the whole tree height. And except for the second TZ sample of CAD, expression levels of F3’H, PAL, and CAD in TZ also gradually increased with tree height. In addition, several transcripts encoding HCT, AMY, and SuSy were expressed at low levels for SW across all tree heights/ages (Fig. 8, Table S11). The expression level of these transcripts varied between SW and TZ in different level of tree heights, and notably, the TZ generally followed few rules.

## Discussion

### Relationship between enzyme activities and HW formation

Dehon found that flavan-3-ols were abundantly localized in the TZ and hypothesized that they are physiological substrates of peroxidases [35]. Enzyme activity levels related to phenylpropanoid and flavonoid biosynthesis pathways (HCT, F3’H, CAD, and PAL) were also high in TZ of *A. melanoxylan*. By contrast, the enzyme activity level of CCR is high not only in TZ but also in SW. Besides, enzyme activity levels of SuSy and AMY from starch and surcose metabolism were higher in TZ than in SW. This is consistent with our microscopic observation of cells. Earlier research found that ethylene concentration was also increased in the TZ [16]. However, activities level of ACO is high both in TZ and in SW. All in all, from the aspect of enzyme activity, we speculated that HW began to form in the TZ of *A. melanoxylan*. Besides, enzyme activities of POD and PPO were found to be involved in processes of HW formation [26, 35]. Generally, the highest activity was found in the cambial zone and the SW. A peak of activity was also observed in the TZ of *A. melanoxylan*, the same as walnut trunk and *Phoebe hui* [29, 35].

### Balance of plant hormones during HW formation

Plant hormones GA, IAA, ETH, CTK, SA, and JA have been proved to affect the formation of wood [14, 36–39]. Early research found that ethylene and auxin may influence the formation process of HW [6, 12]. In our study, even though ACO activity was high in both SW and TZ, RNAs were substantially expressed in the TZ than in SW. This is similar to Lim’s research results [6]. Meanwhile, our results that IAA concentration was reduced in the TZ is consistent with the results of the previous study [15]. In addition to the increase in ethylene concentration, GA, JA, SA, and CTK were also increased in TZ, which is similar to the result of *Aquilaria sinensi* [26]. Our findings reveal that auxin may have an antagonistic effect not only with ethylene, but also with other hormones during the HW formation process of *A. melanoxylon*. Moreover, in our study, transcripts related to these hormones (GA, ETH, CTK, SA, and JA) have been proved to be up-regulated in TZ of *A. melanoxylon*. And several TFs related to auxin response, however, were downregulated in TZ. Therefore, in future breeding, the genes corresponding to these five hormones (GA, ETH, CTK, SA, and JA) can be induced to up-regulate expression in the TZ and reduce the expression of auxin-related genes, which may promote the formation of HW.

### Regulation of DEGs and DEMs related to phenylpropanoid and flavonoid pathways in HW formation

HW formation is generally characterized by the accumulation of phenolic substances [32]. The first enzyme of the phenylpropanoid pathway, PAL, catalyzes the deamination of phenylalanine to yield cinnamic acid. CAD catalyzes the conversion of hydroxycinnamyl aldehydes to alcohols [40]. The genes encoding PAL, CAD, and COMT in phenylpropanoid biosynthesis showed high expression in TZ of Scots Pine [6, 41]. In our study, the genes encoding PAL, CCR, COMT, and CAD were also up-regulated strongly in the TZ of *A. melanoxylon*. It has been proved that the accumulations of metabolites in the TZ and HW are closely correlated with the expression levels of the genes from phenylpropanoid biosynthesis [34, 42]. Based on our results in phenylpropanoid biosynthesis and metabolism, we found that three metabolites (sinapoyl aldehyde, sinapyl alcohol, 4-hyroxycinnamic acid) were significantly abundant accumulated in HW and TZ. Flavonoid biosynthesis has also proved to be an important factor during the process of HW formation [41]. CHS catalyzes the first step of flavonoid biosynthesis by directing carbon flux from phenylpropanoid to the flavonoid metabolism pathway [30]. HCT catalyzes the reactions both immediately preceding and following p-Coumaroylquinic acid by the insertion of the hydroxyl group [43]. In our results, HCT, CHS, CHI, and F3’H, as key genes of flavonoid biosynthesis in HW [23, 44], were also highly expressed in TZ of *A. melanoxylon*. Meanwhile, four phenolic-related metabolites, 2,3,4,4,6-peptahydroxy-chalcone 4-o-glucoside, (-)-epicatechin, (-)-epigallocatechin, and isovitexin 2-o-beta-d-glucoside from were also up-regulated in the HW. Epicatechin and epigallocatechin-3-O-gallate were also identified in the HW of *Acacia catechu* [45]. Several DEMs regulated by genes have been proven to be related to the metabolites of HW formation, such as Celdon et al. characterized SaCYP736A167 as a multi-substrate P450, which stereo-selectively produces (Z)-a-santalol, (Z)-b-santalol, (Z)-epi-b-santalol and (Z)-a-exo-bergamotol, matching authentic sandalwood oil of HW [8]. Yeh et al. found that the up-regulation expression of several genes (PAL, C4H, HCT, COMT, and CCR) was related to regulating secondary metabolite biosynthesis in TZ [42]. These findings reveal that the up-regulation of differential genes and metabolites is related to the HW formation of *A. melanoxylon*. Furthermore, we speculate that the up-regulated expression of these genes is related to the differential accumulation of these metabolites in SW, TZ, and HW of *A. melanoxylon*.

On the other hand, in addition to the high content of these metabolites in the TZ and HW, the activities of the four enzymes (HCT, F3’H, CAD, and PAL) are also higher in the TZ (Fig. 3d, e, f, g), and the contents of total phenolics and total flavonoids are higher in the TZ (Fig. 2c, d). Based on these findings, we speculate that the type of HW formation for *A. melanoxylon* is similar to the type I or *Robinia*-Type reported by Kampe and Magel [46]. However, this is slightly different from the type I recently described by Celedon et al. in three models of HW formation [7], because our phenolics and flavonoids continue to accumulate largely in the HW.

### From carbon skeletons to terpenoids biosynthesis

The existence of starches in SW has been proved by most tree species containing HW [26, 29, 47]. As we can see, starch concentration in TZ was dramatically reduced, and histochemical results suggested starches decreased. The most important contributors to starch and sucrose metabolism were sucrose synthase (SuSy) and alpha-amylase (AMY) [6, 48, 49]. Our study found that both transcripts encoding by these enzymes were up-regulated 28–85 and 7-172 times in TZ than in SW, respectively. Moreover, Transcripts related to amino sugar and nucleotide sugar metabolism and pentose phosphate pathway were also up-regulated in TZ. These findings indicated that sugar metabolism was diverted downstream to produce precursors to the terpenoid backbone.

Glycolysis is a transition between sugar metabolism and terpenoid backbone biosynthesis. Hydroxymethylglutaryl-CoA synthase converts acetoacetyl-CoA into 3-hydroxy-3-methylglutaryl (HMG)-CoA, which is essential for terpenoid backbone biosynthesis [50]. Transcripts for the enzyme were significantly up-regulated in TZ than in SW. The mevalonate is a target for metabolic engineering because it supplies precursors for isoprenoid biosynthesis [51]. Mevalonate kinase (MK) and phosphomevalonate kinase (PMK), converted mevalonate into mevalonate-5pp, were strongly found to be up-regulated in TZ. Up-regulation of transcripts may indicate that mevalonate is used not only as a direct source of mevalonic acid but also as a precursor for terpenoid biosynthesis.

Geranylgeranyl diphosphate (GGPP) produced by GGDS serves as a precursor for many diphosphates, including farnesyl-PP, Geranylgeranyl-PP, and Geranyl-PP [52, 53]. Farnesyl-diphosphate (FPS) reduced farnesyl-PP to squalene, and then squalene monooxygenase (SME) oxidized squalene to (s)-squalene-2, 3-epoxide in sesquiterpenoid and triterpenoid biosynthesis [54, 55]. Up-regulation of FPS and SME transcripts may indicate a relatively high accumulation of F-PP and squalene for sesquiterpenes and monoterpenes in HW. In the diterpenoid biosynthesis pathway, the genes encoding geranyllinalool synthases (GES), which are responsible for the biosynthesis of geranyllinalool [56], were also to up-regulated strongly in TZ. (-)-terpineol synthase (ATS) from the TPS subfamily can catalyze the formation of alpha-terpineol [57, 58]. ATS-related transcripts were also up-regulated in TZ, meaning the main monoterpenoid was alpha-terpineol. These findings suggested that the quantity and diversity of terpenoids in *A. melanoxylon* HW. Furthermore, combining the results of total sugar and total terpene and the substantial upregulation of transcripts involved in terpenoid biosynthesis, we suggested that these extractives were mainly produced in the TZ, with carbon skeleton provided by sucrose. The type of HW formation in *A. melanoxylon* is the type I in this respect [6].

### Transcriptional control during HW formation

Several candidate transcription factors: MYB12, MYB1, AP2-ERF, bZIP, NAC, MYB, C2C2, C2H2, and GRAS are shown to be associated with metabolite flux and the phenylpropanoid pathway to the production of flavonols/anthocyanin [59]. TFs in *Camellia sinensis*, such as MYB, AP2/ERF, bZIP, and TCP, were dramatically expressed and focused on the regulation of genes in the upstream synthesis of phenylalanine but inhibited the formation of flavones [60]. In our study, AP2 and bZIP were highly expressed in TZ and had a positive correlation with phenolics and flavonoids. Besides, CBF, PB1, and TCP had a negative correlation with phenolics and flavonoids of *A. melanoxylon*. On the other hand, studies have revealed that MYB and WRKY TFs were also involved in the regulation of genes from the flavonoid and phenylpropanoid biosynthesis pathway [61, 62]. MYB and WRKY were highly expressed in TZ, but there is still a negative correlation with the biosynthesis of phenolics and flavonoids. Phenolics and flavonoids are important components of the extract during HW formation [7, 8]. We speculate that these transcription factors are also the key factors affecting the HW formation of *A. melanoxylon*.

### PCD and dehydration may be related to HW formation

Ray parenchyma cell death in wood, combined with tylosis development, wood dryness, and accumulation of HW extracts, changes SW to HW [63]. The process of HW formation is accompanied by the dehydration of tracheids, the death of ray parenchyma cells, and the deposition of HW extract, which leads to wood coloring [5, 47]. Studies found that dehydration of tracheids at the boundary between SW and intermediate wood occurred continuously. Upon binding to CKAP5, T-DM1 (Ado-trastuzumab emtansine) causes cell membrane damage and leads to calcium influx into the cells, resulting in a disorganized microtubule network and apoptosis [64]. Aquaporins have also been proved to be involved in regulating water transport between cells and maintaining water balance [65, 66]. So, up-regulated CKAP and DRP in the TZ may explain that PCD and dehydration are involved in the HW formation of *A. melanoxylon*. The up regulation of transcripts related to PCD and CKAP in TZ may be a critical symbol for distinguishing programmed HW formation and extract biosynthesis in *A. melanoxylon*.

### HW formation pattern along the tree height/tree age

Nucleic acids are degraded, and cell structure is lost during the SW-to-HW transition [46]. Nucleic acid fragmentation increased with sample age [67]. In our study, in the TZ between SW and HW, the increased expression of transcripts encoding F3’H, PAL, and CAD may be related to the decrease of tree age/tree height (Fig. 8). Expression patterns of transcription factors in lignified tissues are dissimilar in different tree age. For example, TgMYB2 expression is higher in young lignified tissues in the *Tectona grandis* [68]. We also found that WRKY71 presented higher expression in young TZ tissues of *A. melanoxylon* (Fig. 8). We boldly speculate that the higher expression of these transcripts may be related to the age of the wood. It is undeniable that the expression level of the xylem transcriptome is significantly affected by tree age and season [69].

## Conclusion

The goal of our study was to preliminarily judge the biochemical and molecular foundation of HW formation of *A. melanoxylon*. Microstructure status, hormone and enzyme activities, differential genes, and metabolites were compared using SW, TZ, and HW of *A. melanoxylon*. Ten metabolites were dominantly in phenolics and flavonoids in the HW formation of *A. melanoxylon*. Both microscopic observation and content determination proved that the total amount of starches decreased, and phenolics and flavonoids increased gradually from SW to HW. The transcripts of plant hormone, PCD, and dehydration were also increased in TZ. Furthermore, the increased expression of sugar and terpenoid biosynthesis-related genes in TZ also explained why the biosynthesis of terpenoids is closely related to carbohydrate metabolites of *A. melanoxylon*. These proved that the HW formation of *A. melanoxylon* occurred in the TZ, and the HW formation may be related to plant hormones, PCD, and cell dehydration. And the increased expression of sugar- and terpenoid biosynthesis-related genes in TZ also explained that the biosynthesis of terpenoids is related to carbohydrate metabolites of *A. melanoxylon*. We also found that higher expression of transcripts in TZ may be related to the age of wood. Moreover, the integrated analysis of metabolome and transcriptome showed the key transcription factors (TFs) regulating flavonoids and phenolics accumulation in HW. And the genes and metabolites from phenylpropanoid and flavonoid metabolism and biosynthesis were up-regulated and largely accumulated in TZ, respectively. The test of RT-qPCR and enzyme activity verified the accuracy of these differentially expressed genes and enzymes. Our findings have vital significance for a comprehensive understanding of the HW formation of *A. melanoxylon* and provide the basis for improving HW yield breeding in the future. The molecular functions of relevant genes need further verification for assessing the detailed mechanism of the HW formation. We may alter the metabolic processes of plants through external stimuli or disturbances (to increase the metabolites that deepen the color of HW), which may promote the formation of HW and thereby enhance the commercial value of *A. melanoxylon*.

## Material and method

### Plant material

A schematic diagram of the HW, SW, and TZ is shown in Figure S1. The SW, HW, and TZ of *A. melanoxylon* clone SR25 were selected at diameter in breast height (DBH) for plant material at the end of December 2021. The ownership of SR25 clone belongs to the Research Institute of Tropical Forestry, Chinese Academy of Forestry. Professor Zeng Bingshan is the official appraiser of this plant material. And, this plant material is not in the public specimen bank and belongs to the protection period of new varieties. The samples were all collected from 10-year-old trees cultured in an experimental forest of Zhongba Town, Heyuan City, Guangdong Province, China (23.40 N, 115.19E). Except for the samples used for microscopic observation, the samples were taken after cutting down trees, and the rest were taken with growth cones (no trees were cut down). A total of 6 trees from the exact clone were drilled 15 incremental cores at DBH and immediately put in liquid nitrogen for subsequent determination. In addition, according to the previous research on HW clones, it is found that there is almost no difference between the exact clones on the same site [4]. According to the color difference of HW, TZ, and SW of each tree, it is divided into 6, 1 and 3 annual tree rings, respectively. And we selected SW and HW with 1–2 annual rings facing the bark.

### Microscopic observation of the three regions (SW, TZ and HW)

The stem Sects. (3–4 cm long) of *A. melanoxylon* SR25 were collected from the main stem at DBH of three trees according to the position (SW, TZ, and HW). Then, stem sections were cut into blocks (1 × 1 × 1 cm3) and put into FAA stationary liquid. Samples were rinsed in buffer and then deionized water before dehydrated in an ethanol series and kept in 75% ethanol. The samples were rewashed before slicing. Subsequently, 18 μm sections were sliced from the blocks using a Semi-automatic microtome (Leica RM2255, Germany). Sections were stained with Periodic Acid-Schiff (PAS) and iodine-potassium iodide (I_2_-KI) for 20 min, washed and mounted in glycerol. All sections were observed under an optical microscope (OLYMPUS BX51, Japan) with DPContro software. More than twenty 1 mm^2^ visual fields were photographed and saved with cross-sections from every position (SW, TZ, and HW).

### Non-structural carbohydrate content detection

The stem sections’ collection time and method were the same as above. The stem sections were dried to a constant weight and crushed with a 40-mesh sieve. Non-structural carbohydrates and lignin content were detected by Shanghai Jingkang Biology Engineering Co., Ltd (Shanghai, China). The detection of starch was based on the anthrone colorimetric method, and absorbance at 620 nm was read [70]. The determination of reducing sugar was taken using the Miniaturization-DNS method with absorbance at 540 nm [71].

### Total phenolics, total terpenes, and total flavonoids contents assay

The sample collection method of total flavonoids was the same as above. Subsequently, about 0.1 g samples were added to 1 mL 60% ethanol solution, and ultrasonic extraction was used to extract the supernatant [72]. Finally, the content of total flavonoids in the extract was detected by a spectrophotometer with the absorbance at 470 m with the Tannin as standard by micro method [73]. The sample collection method of total phenolics and terpenes was the same as above. The content of total phenolics was detected at 760 nm absorbance with the Gallic acid as standard by micro method [73]. The content of total terpenes was determined with monoterpenes and aromatic alcohols as standard by vanillin-glacial acetic acid colorimetric method [74], and absorbance at 535 nm was read.

### Hormone and enzyme contents measurement

At first, the wood core from the stem section was drilled and collected at DBH from six SR25 clones using a growth cone. Secondly, the drilled wood cores were quickly cut into pieces according to the position (SW, TZ, and HW) with surgical scissors, put into a tin paper bag, and then quickly put into liquid nitrogen for preservation. Stem samples were sent to the Shanghai Jingkang Biology Engineering Co., Ltd (Shanghai, China) for determination of phytohormone contents [gibberellin (GA), jasmonic acid (JA), auxin (IAA), salicylic acid (SA) and ethrel (ETH)] and Enzyme contents [4-coumaric Acid Coenzyme Aligase (4CL), ACC oxidase (ACO), Alpha-amylase (AMS), Phenylalanine ammonia-lyase (PAL), Polyphenol Oxidase (PPO), Peroxidase (POD), naringenin 3-dioxygenase (F3’H), Shikimate O-hydroxycinnamoyltransferase (HCT), Cinnamyl-alcohol dehydrogenase (CAD), Cinnamoyl-CoA reductase (CCR), Sucrose synthase (SuSy)].

### Metabolite extraction and profiling

The Metabolite extraction: (1) Accurately weigh 200 mg (± 1%) of the sample into a 2 mL EP tube, accurately add 0.6 mL of methanol (including internal standard), add 100 mg of glass beads, and vortex for 1 min, (2) Grind at 50 Hz for 60 s in a high-throughput tissue grinder and repeat twice, (3) Room temperature ultrasound for 15 min, (4) Centrifuge at 12,000 rpm4 ℃ for 10 min, take 200 µL supernatant, and add it to the detector bottle, (5) Take 20 µL from each sample to the quality control (QC) samples. (These QC samples were used to monitor deviations of the analytical results from these pool mixtures and compare them to the errors caused by the analytical instrument itself).

A mixed mix standard curve was prepared as the study of Chen [75, 76]. Then, chromatographic separation was operated by an ACQUITY UPLC® HSS T3 (150 × 2.1 mm, 1.8 μm, Waters) column with previous research methods [77]. Mass spectrometry conditions are as follows. The Orbitrap analyzer scanned over a mass range of m/z 100-1 000 for the full scan at a mass resolution of 60 000. Data-dependent acquisition (DDA) MS/MS experiments were performed with an HCD scan. Dynamic exclusion was implemented to remove unnecessary information in MS/MS spectra [77, 78]. Finally, the original data was obtained with format conversion like Smith [79]. The relative quantitative analysis is based on the method of UPLC/HRMS. Metabolite identification was performed using the BioMarker database [79]. Ropls package in R language is used to describe the principal component analysis of metabolites in different groups (SR25HW vs. SR25SW, SR25TZ vs. SR25SW, and SR25HW vs. SR25TZ vs. SR25SW). *P*-value ≤ 0.05 and VIP ≥ 1, one-way ANOVA *p*-value ≤ 0.05 and VIP ≥ 1, the two methods were used to screen differential metabolites [80, 81]. The pheatmap package of R (v3.3.2) is used to cluster the relative values of DEM. And the Z score (standard score) is converted based on the relative content of metabolites. KEGG (Kyoto Encyclopedia of Genes and Genomes) is used to determine the enrichment pathway of differential metabolism. MetPA (www.metaboanalyst.ca) was used to analyze the related metabolic pathways of different metabolites in each group, and the related network diagram of metabolic pathways was drawn according to the results.

### RNA extraction

The separated SW and TZ were used for extracting RNA. And RNA-seq was detected by the Illumina NovaSeq6000 platform. Accurately weighing 100 mg (± 1%) of the sample was used for metabolite extraction preparation. Total RNA was extracted from SW, HW, and TZ using the Aidlab Plant RNA extraction Kit called EASY Spin (Aidlab Biotech, Beijing, China). HW, SW, and TZ are all three biological repetitions.

### The transcriptome assembly, annotation, and analysis

The transcriptomes of *A. melanoxylon* were developed by the Illumina NovaSeq6000 platform. After the library passed the quality inspection, the PE150 pattern was sequenced. And clean data were mapped to the assembled *A. melanoxylon* genome. Mapped data were then used for the library quality evaluation, structural level analysis, and differential expression analysis [82]. In this project, DEGs were identified with Fold Change ≥ 2 and FDR < 0.01. Then GO/KEGG enrichment, GSEA, differential alternative splicing (AS), and protein interactions of DEGs were analyzed. Gene function was annotated by BLASTP, and SwissProt, Nr, Pfam, KEGG and InterPro were used to screen the functional domains of the proteins.

Heatmap, GO and KEGG enrichment analysis were performed by using Tbtools v1.045 [83]. The iTAK software was used to classify TFs within the module into different families [84].

### Correlation analysis of metabolites and TFs

Differentially expressed TFs were subjected to an association analysis of differentially accumulated phenylpropanoids and flavonoids. The correlation analysis in both metabolite intensity and expression level of transcripts was detected by calculating the Pearson correlation coefficient (PCC). Cytoscape version 3.9.1 (Cytoscape Consortium, USA) was used to draw the network map between TFs and metabolites (screening criterion was |PCC| ≥ 0.8).

### Expression validation of the genes with RT-PCR (real-time quantitative)

We collected samples from the living trees that had just been cut down. The samples were collected quickly with a sterilized chisel and hammer from the base or 0, 1/4, 1/2, and 3/4 of the trunk in the SW and the TZ. And we selected the SW with 1–2 annual rings facing the bark. Total RNA was isolated from the SW and TZ. For the qRT-PCR analysis, the Monad cDNA Kit (Monad, China) was used to synthesize the cDNA, and the SYBR-Green PCR kit (QIAGEN, Germany) was used to obtain a reagent mixture for the PCR test. Then, ABI7500 (ABI, USA) was used to perform the qPCR procedure. The expression level of genes was calculated in triplicate based on the 2^−ΔΔCt^ algorithm, and RPL4 was used as the internal reference gene. The primer sequences designed using Primer3Plus.

### Electronic supplementary material

Below is the link to the electronic supplementary material.

**Additional file 1: Figure S1**. A schematic diagram of the HW, SW, and TZ in disc at breast height of *A. melanoxylon.*

**Additional file 2: Table S1**. Determination data of key substances in three positions (SW, TZ, and HW) of *A. melanoxylon*. Note: Data are presented in the mean ± SE. Different capital letters indicate that the treatment effect is significantly different at the *p* < 0.05 level.

**Additional file 3: Figure S2**. Differential metabolite cluster thermogram between four groups. (a–d) Figures a-d belong to comparison groups HW vs. TZ, HW vs. SW, TZ vs. SW, HW vs. TZ vs. SW respectively.

**Additional file 4: Table S2**. Determination data of enzyme activity related to HW formation in three positions (SW, TZ, and HW) of *A. melanoxylon*. Note: Data are presented in the mean ± SE. Different capital letters indicate that the treatment effect is significantly different at the *p* < 0.05 level

**Additional file 5: Figure S3**. Structure and peak area of metabolites involved in HW formation.

**Additional file 6: Table S3**. Determination data of plant hormones in three positions (SW, TZ, and HW).

**Additional file 7: Figure S4**. Regulatory map of gene expression related to phenylalanine and flavonoid biosynthesis. PAL: phenylalanine ammonia-lyase; AAS: aromatic-L-amino-acid decarboxylase; AO: primary-amine oxidase; 4CL: Phenylalanine ammonia-lyase; C4H: trans-cinnamate 4-monooxygenase; CCR: cinnamoyl-CoA reductase; CHS: chalcone synthase; 4HA: 4-Hydroxycinnamyl aldehyde; F5H: ferulate-5-hydroxylase; COMT: caffeic acid 3-o-methyltransferase; CAD: cinnamyl-alcohol dehydrogenase; HCT: shikimate O-hydroxycinnamoyltransferase; CHI: chalcone isomerase; FLSII: flavone synthase II; CHS: chalcone synthase; VORM: vitexin 2’’-O-rhamnoside 7-O-methyltransferase. The arrows in the figure represent enzymatic reactions.

**Additional file 8: Table S4**. Differential expressed metabolites identified in three groups.

**Additional file 9: Figure S5**. Pathway diagram of plant hormone signal transduction of *A. melanoxylon*. Note, the red box represents only the up-regulation sequence, and the blue box represents both up-regulated genes and down-regulated sequences.

**Additional file 10: Table S5**. KEGG enriched analysis of 34 metabolites for *A. melanoxylon*.

**Additional file 11: Figure S6**. Pathway diagram of MAPK signal (plant) of *A. melanoxylon*. Note, the red box represents only the up-regulation sequence, the green box represents only the down-regulation sequence, and the blue box represents both up-regulated genes and down-regulated sequences. The dotted box is framed to describe the object.

**Additional file 12: Table S6**. Statistics of differential expressed genes in SR25SW vs. SR25TZ.

**Additional file 13: Table S7**. Differential expressed genes related to plant hormones in SR25SW vs. SR25TZ.

**Additional file 14: Table S8**. Connection network between TFs and metabolites related to phenylpropanoids and flavonoids.

**Additional file 15: Table S9**. Differential expressed genes of terpenoids and sucrose metabolism identified in SR25SW vs. SR25TZ.

**Additional file 16: Table S10**. Gene differential expression of qRT-PCR in horizontal and vertical directions of the trunk.

**Additional file 17: Table S11**. Raw data of FPKM related to the genes of RT-PCR in SR25SW vs. SR25TZ.

## Data Availability

Sequence data that support the findings of this study have been deposited in the NGDC database under BioProject. However, the date of publicity is December 31(st), 2024. And we can also publish the raw data after the article is published.
