# Supplementary material for: Comparative physiological, biochemical, metabolomic, and transcriptomic analyses reveal the formation mechanism of heartwood for Acacia melanoxylon
Source: BMC Plant Biol. 2024 Apr 22;24:308. doi: 10.1186/s12870-024-04884-1 (PMC11034122; doi:10.1186/s12870-024-04884-1)
Supplement: Supplementary file 1 — Additional file 1: Figure S1. A schematic diagram of the HW, SW, and TZ in disc at breast height of A. melanoxylon. [file 12870_2024_4884_MOESM1_ESM.docx]

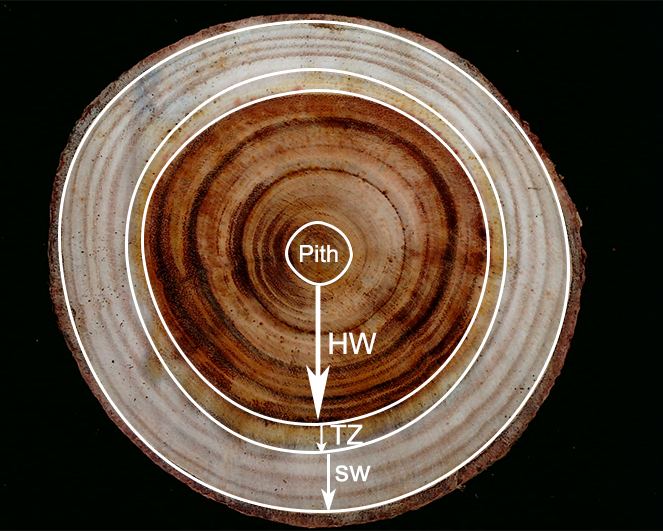


**Additional file 1:FigureS1.** A schematic diagram of the HW, SW, and TZ in disc at breast height of *A. melanoxylon*
