# Supplementary material for: Comparative physiological, biochemical, metabolomic, and transcriptomic analyses reveal the formation mechanism of heartwood for Acacia melanoxylon
Source: BMC Plant Biol. 2024 Apr 22;24:308. doi: 10.1186/s12870-024-04884-1 (PMC11034122; doi:10.1186/s12870-024-04884-1)
Supplement: Supplementary file 2 — Additional file 2: Table S1. Determination data of key substances in three positions (SW, TZ, and HW) of A. melanoxylon. Note: Data are presented in the mean ± SE. Different capital letters indicate that the treatment effect is significantly different at the p < 0.05 level. [file 12870_2024_4884_MOESM2_ESM.docx]

**Additional file 2:Table S1.** Determination data of key substances in three positions (SW, TZ, and HW) of *A. melanoxylon*. Note: Data are presented in the mean ± SE. Different capital letters indicate that the treatment effect is significantly different at the p ＜ 0.05 level.

| substances | encode | SR25-SW | SR25-TZ | SR25-HW |
| --- | --- | --- | --- | --- |
| satrches | average | 10.68 | 9.98 | 9.84 |
|  | **STD** | 0.114431964 | 0.110479843 | 0.156114979 |

| substances | encode | SR25-SW | SR25-TZ | SR25-HW |
| --- | --- | --- | --- | --- |
| phenolics | average | 2.48 | 2.69 | 4.39 |
|  | **STD** | 0.035582196 | 0.048851191 | 0.046634641 |

| substances | encode | SR25-SW | SR25-TZ | SR25-HW |
| --- | --- | --- | --- | --- |
| flavonoids | average | 4.32 | 4.68 | 4.90 |
|  | **STD** | 0.056330477 | 0.070147827 | 0.058808539 |

| substances | encode | SR25-SW | SR25-TZ | SR25-HW |
| --- | --- | --- | --- | --- |
| surgars | average | 231.19 | 235.94 | 193.92 |
|  | **STD** | 2.489862781 | 3.437356058 | 3.313558007 |

| substances | encode | SR25-SW | SR25-TZ | SR25-HW |
| --- | --- | --- | --- | --- |
| terpenoids | average | 6.00 | 7.29 | 7.88 |
|  | **STD** | 0.061557701 | 0.076323376 | 0.098244226 |
