# Supplementary material for: Comparative physiological, biochemical, metabolomic, and transcriptomic analyses reveal the formation mechanism of heartwood for Acacia melanoxylon
Source: BMC Plant Biol. 2024 Apr 22;24:308. doi: 10.1186/s12870-024-04884-1 (PMC11034122; doi:10.1186/s12870-024-04884-1)
Supplement: Supplementary file 3 — Additional file 3: Figure S2. Differential metabolite cluster thermogram between four groups. (a–d) Figures a-d belong to comparison groups HW vs. TZ, HW vs. SW, TZ vs. SW, HW vs. TZ vs. SW respectively. [file 12870_2024_4884_MOESM3_ESM.docx]

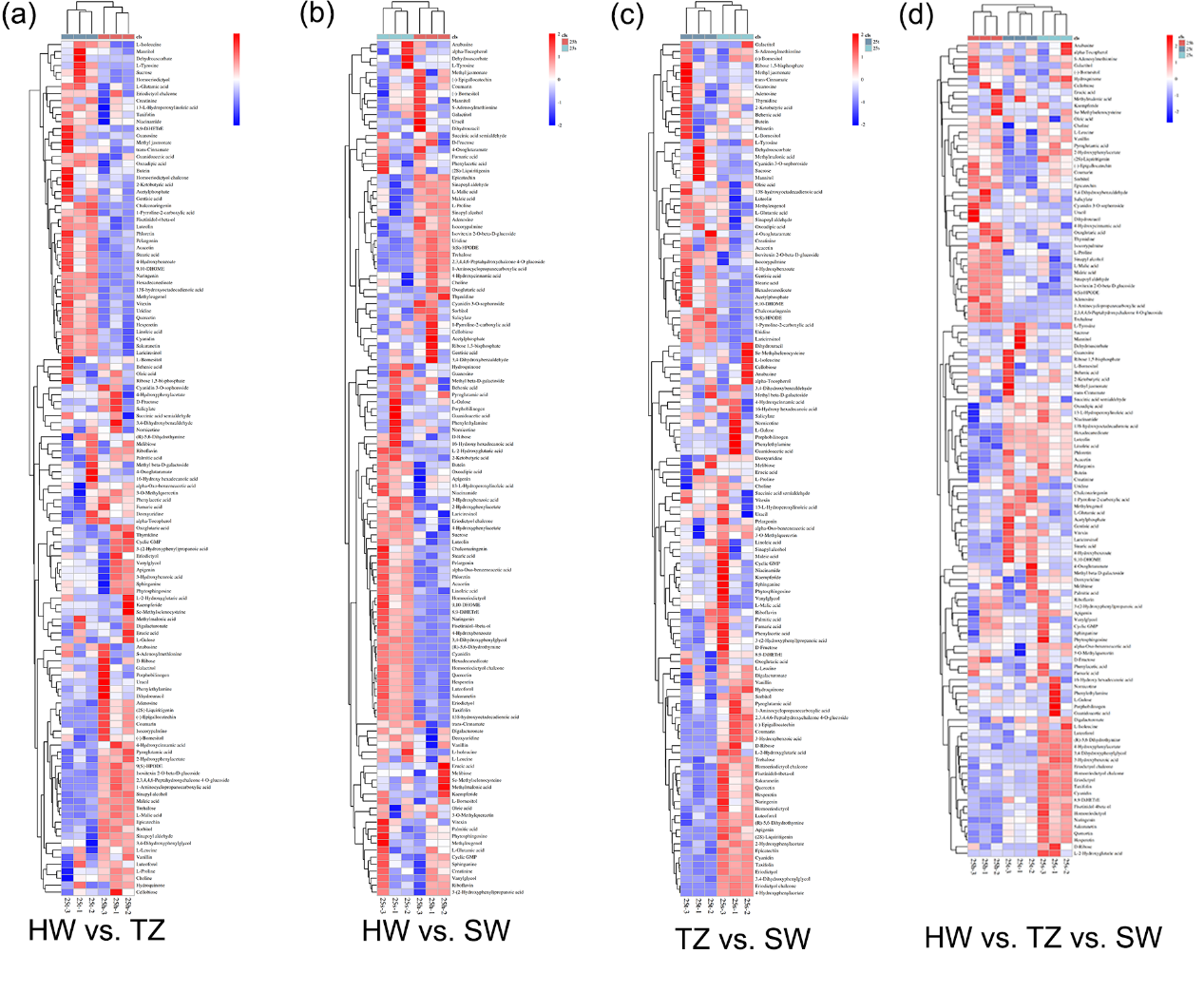
**Additional file 3:Figure S2.** Differential metabolite cluster thermogram between four groups. (a–d) Figures a-d belong to comparison groups HW vs. TZ, HW vs. SW, TZ vs. SW, HW vs. TZ vs. SW respectively.
