# Supplementary material for: Comparative physiological, biochemical, metabolomic, and transcriptomic analyses reveal the formation mechanism of heartwood for Acacia melanoxylon
Source: BMC Plant Biol. 2024 Apr 22;24:308. doi: 10.1186/s12870-024-04884-1 (PMC11034122; doi:10.1186/s12870-024-04884-1)
Supplement: Supplementary file 4 — Additional file 4: Table S2. Determination data of enzyme activity related to HW formation in three positions (SW, TZ, and HW) of A. melanoxylon. Note: Data are presented in the mean ± SE. Different capital letters indicate that the treatment effect is significantly different at the p < 0.05 level [file 12870_2024_4884_MOESM4_ESM.docx]

**Additional file 4:Table S2.** Determination data of enzyme activity related to HW formation in three positions (SW, TZ, and HW) of *A. melanoxylon*. Note: Data are presented in the mean ± SE. Different capital letters indicate that the treatment effect is significantly different at the p ＜ 0.05 level.

| enzyme | encode | SR25-SW | SR25-TZ | SR25-HW |
| --- | --- | --- | --- | --- |
| ACO | ACO-1 | 496.78 | 454.75 | 376.63 |
|  | ACO-2 | 491.83 | 447.27 | 387.19 |
|  | ACO-3 | 487.43 | 415.69 | 387.08 |
|  | ACO-4 | 491.06 | 421.74 | 386.09 |
|  | ACO-5 | 443.31 | 468.72 | 423.50 |
|  | ACO-6 | 448.37 | 452.55 | 403.15 |
|  | 平均值 | 476.4595381 | 443.4517844 | 393.9401539 |
|  | **STD** | 23.96136254 | 20.52124095 | 16.80706166 |
| AMY | AMY-1 | 3.99 | 4.62 | 4.12 |
|  | AMY-2 | 4.04 | 4.59 | 4.10 |
|  | AMY-3 | 4.32 | 4.92 | 4.53 |
|  | AMY-4 | 4.33 | 4.87 | 4.45 |
|  | AMY-5 | 4.52 | 4.29 | 4.33 |
|  | AMY-6 | 4.43 | 4.51 | 4.30 |
|  | 平均值 | 4.27353337 | 4.633512877 | 4.306694422 |
|  | **STD** | 0.211599115 | 0.231039503 | 0.17372541 |
| PAL | PAL-1 | 39.87 | 43.66 | 39.56 |
|  | PAL-2 | 39.80 | 43.72 | 40.75 |
|  | PAL-3 | 39.14 | 40.52 | 42.04 |
|  | PAL-4 | 38.97 | 41.51 | 42.44 |
|  | PAL-5 | 42.37 | 44.72 | 43.69 |
|  | PAL-6 | 40.90 | 44.07 | 41.78 |
|  | 平均值 | 40.17724844 | 43.03450284 | 41.71136876 |
|  | **STD** | 1.273145887 | 1.639074223 | 1.421459608 |
| PPO | PPO-1 | 146.08 | 153.00 | 107.19 |
|  | PPO-2 | 141.73 | 151.03 | 108.54 |
|  | PPO-3 | 137.34 | 137.06 | 119.05 |
|  | PPO-4 | 136.78 | 142.90 | 115.39 |
|  | PPO-5 | 138.06 | 152.27 | 109.99 |
|  | PPO-6 | 140.52 | 145.60 | 114.76 |
|  | 平均值 | 140.085881 | 146.9769533 | 112.4870213 |
|  | **STD** | 3.504357116 | 6.285136671 | 4.614904901 |
| POD | POD-1 | 32.91 | 31.27 | 26.06 |
|  | POD-2 | 32.89 | 30.23 | 27.18 |
|  | POD-3 | 31.54 | 30.61 | 25.94 |
|  | POD-4 | 31.55 | 30.12 | 25.75 |
|  | POD-5 | 29.83 | 31.83 | 27.49 |
|  | POD-6 | 30.27 | 30.79 | 26.93 |
|  | 平均值 | 31.4981572 | 30.80777511 | 26.56087923 |
|  | **STD** | 1.28281675 | 0.648304155 | 0.73127989 |
| F3'H | F3H-1 | 212.50 | 262.93 | 229.33 |
|  | F3H-2 | 210.56 | 256.49 | 224.14 |
|  | F3H-3 | 225.28 | 256.66 | 230.98 |
|  | F3H-4 | 226.76 | 259.68 | 235.21 |
|  | F3H-5 | 209.30 | 245.76 | 232.92 |
|  | F3H-6 | 211.81 | 243.71 | 232.30 |
|  | 平均值 | 216.0357793 | 254.2035985 | 230.8122594 |
|  | **STD** | 7.824579507 | 7.731931117 | 3.814218401 |
| HCT | HCT-1 | 162.58 | 194.56 | 175.50 |
|  | HCT-2 | 165.74 | 193.22 | 174.90 |
|  | HCT-3 | 180.25 | 193.31 | 178.43 |
|  | HCT-4 | 174.99 | 192.61 | 180.99 |
|  | HCT-5 | 163.14 | 188.75 | 178.15 |
|  | HCT-6 | 170.16 | 193.73 | 177.08 |
|  | 平均值 | 169.475627 | 192.6974811 | 177.510683 |
|  | **STD** | 7.050873779 | 2.03739902 | 2.208830819 |
| CAD | CAD-1 | 89.29 | 96.75 | 92.75 |
|  | CAD-2 | 91.37 | 96.11 | 93.74 |
|  | CAD-3 | 83.65 | 95.12 | 88.73 |
|  | CAD-4 | 85.31 | 94.13 | 89.54 |
|  | CAD-5 | 89.33 | 96.71 | 93.74 |
|  | CAD-6 | 90.00 | 95.44 | 93.98 |
|  | 平均值 | 88.15637684 | 95.70799155 | 92.07920043 |
|  | **STD** | 2.994060141 | 1.016959013 | 2.333569149 |
| CCR | CCR-1 | 268.83 | 273.22 | 244.10 |
|  | CCR-2 | 269.72 | 269.98 | 247.15 |
|  | CCR-3 | 281.29 | 260.82 | 259.99 |
|  | CCR-4 | 278.11 | 269.85 | 262.16 |
|  | CCR-5 | 268.32 | 281.16 | 249.06 |
|  | CCR-6 | 269.53 | 279.32 | 243.78 |
|  | 平均值 | 272.6350404 | 272.391342 | 251.0412388 |
|  | **STD** | 5.588168032 | 7.373540409 | 8.044741217 |
| SUSY | SUSY-1 | 199.00 | 226.69 | 178.40 |
|  | SUSY-2 | 197.34 | 224.51 | 184.61 |
|  | SUSY-3 | 188.82 | 224.61 | 186.17 |
|  | SUSY-4 | 190.10 | 224.56 | 182.43 |
|  | SUSY-5 | 202.45 | 214.90 | 196.44 |
|  | SUSY-6 | 204.20 | 211.26 | 193.17 |
|  | 平均值 | 196.984472 | 221.0865619 | 186.8702726 |
|  | **STD** | 6.329119166 | 6.362728162 | 6.757628949 |
