# Supplementary figures and images for: Comparative physiological, biochemical, metabolomic, and transcriptomic analyses reveal the formation mechanism of heartwood for Acacia melanoxylon
Source: BMC Plant Biol. 2024 Apr 22;24:308. doi: 10.1186/s12870-024-04884-1 (PMC11034122; doi:10.1186/s12870-024-04884-1)

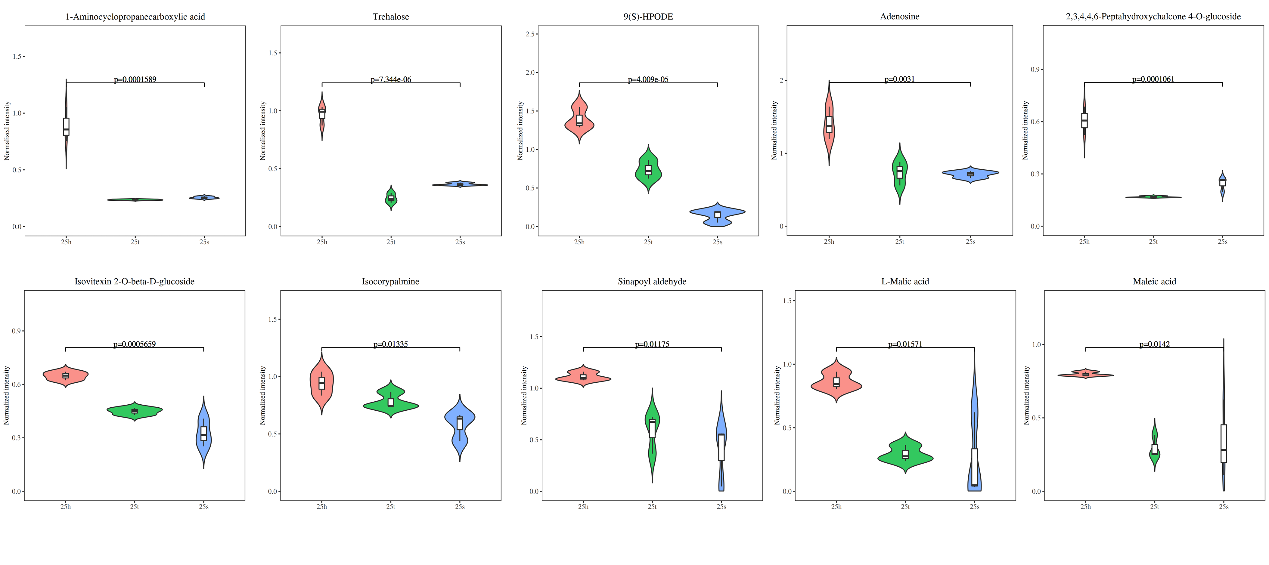
**Additional file 5:FigureS3.** Structure and peak area of metabolites involved in HW formation

Supplement: Supplementary file 5 — Additional file 5: Figure S3. Structure and peak area of metabolites involved in HW formation. [file 12870_2024_4884_MOESM5_ESM.docx]
