# Supplementary material for: Comparative physiological, biochemical, metabolomic, and transcriptomic analyses reveal the formation mechanism of heartwood for Acacia melanoxylon
Source: BMC Plant Biol. 2024 Apr 22;24:308. doi: 10.1186/s12870-024-04884-1 (PMC11034122; doi:10.1186/s12870-024-04884-1)
Supplement: Supplementary file 6 — Additional file 6: Table S3. Determination data of plant hormones in three positions (SW, TZ, and HW). [file 12870_2024_4884_MOESM6_ESM.docx]

**Additional file 6**:**Table S3.** Determination data of plant hormones in three positions (SW, TZ, and HW).

| μg/L | GA-1 | GA-2 | GA-3 | GA-4 | GA-5 | GA-6 | AVERAGE | SD |
| --- | --- | --- | --- | --- | --- | --- | --- | --- |
| SR25-SW | 0.49 | 0.50 | 0.54 | 0.52 | 0.48 | 0.50 | 0.51 | 0.02 |
| SR25-TZ | 0.62 | 0.60 | 0.62 | 0.61 | 0.56 | 0.56 | 0.60 | 0.03 |
| SR25-HW | 0.54 | 0.53 | 0.55 | 0.55 | 0.56 | 0.56 | 0.55 | 0.01 |
|  | JA-1 | JA-2 | JA-3 | JA-4 | JA-5 | JA-6 |  |  |
| SR25-SW | 0.25 | 0.25 | 0.27 | 0.27 | 0.27 | 0.26 | 0.26 | 0.01 |
| SR25-TZ | 0.30 | 0.29 | 0.29 | 0.28 | 0.29 | 0.30 | 0.29 | 0.01 |
| SR25-HW | 0.24 | 0.25 | 0.25 | 0.26 | 0.25 | 0.26 | 0.25 | 0.01 |
|  | IAA-1 | IAA-2 | IAA-3 | IAA-4 | IAA-5 | IAA-6 |  |  |
| SR25-SW | 80.74 | 81.05 | 86.38 | 84.58 | 82.52 | 81.44 | 82.79 | 2.25 |
| SR25-TZ | 76.99 | 80.46 | 87.41 | 83.31 | 77.05 | 80.87 | 81.02 | 3.96 |
| SR25-HW | 99.09 | 96.83 | 95.99 | 94.52 | 97.00 | 97.09 | 96.76 | 1.50 |
|  | SA-1 | SA-2 | SA-3 | SA-4 | SA-5 | SA-6 |  |  |
| SR25-SW | 0.19 | 0.19 | 0.18 | 0.19 | 0.19 | 0.20 | 0.19 | 0.00 |
| SR25-TZ | 0.23 | 0.23 | 0.23 | 0.23 | 0.23 | 0.23 | 0.23 | 0.00 |
| SR25-HW | 0.22 | 0.22 | 0.21 | 0.21 | 0.21 | 0.21 | 0.21 | 0.01 |
|  | CTK-1 | CTK-2 | CTK-3 | CTK-4 | CTK-5 | CTK-6 |  |  |
| SR25-SW | 52.90 | 52.86 | 56.56 | 55.78 | 49.96 | 49.92 | 53.00 | 2.80 |
| SR25-TZ | 59.21 | 57.97 | 58.23 | 57.16 | 60.14 | 58.12 | 58.47 | 1.05 |
| SR25-HW | 58.79 | 57.70 | 52.99 | 52.95 | 54.07 | 55.20 | 55.28 | 2.46 |
|  | ETH-1 | ETH-2 | ETH-3 | ETH-4 | ETH-5 | ETH-6 |  |  |
| SR25-SW | 0.41 | 0.41 | 0.40 | 0.40 | 0.43 | 0.42 | 0.41 | 0.01 |
| SR25-TZ | 0.46 | 0.46 | 0.44 | 0.43 | 0.44 | 0.43 | 0.44 | 0.01 |
| SR25-HW | 0.45 | 0.43 | 0.44 | 0.44 | 0.44 | 0.44 | 0.44 | 0.01 |
