# Supplementary material for: Comparative physiological, biochemical, metabolomic, and transcriptomic analyses reveal the formation mechanism of heartwood for Acacia melanoxylon
Source: BMC Plant Biol. 2024 Apr 22;24:308. doi: 10.1186/s12870-024-04884-1 (PMC11034122; doi:10.1186/s12870-024-04884-1)
Supplement: Supplementary file 7 — Additional file 7: Figure S4. Regulatory map of gene expression related to phenylalanine and flavonoid biosynthesis. PAL: phenylalanine ammonia-lyase; AAS: aromatic-L-amino-acid decarboxylase; AO: primary-amine oxidase; 4CL: Phenylalanine ammonia-lyase; C4H: trans-cinnamate 4-monooxygenase; CCR: cinnamoyl-CoA reductase; CHS: chalcone synthase; 4HA: 4-Hydroxycinnamyl aldehyde; F5H: ferulate-5-hydroxylase; COMT: caffeic acid 3-o-methyltransferase; CAD: cinnamyl-alcohol dehydrogenase; HCT: shikimate O-hydroxycinnamoyltransferase; CHI: chalcone isomerase; FLSII: flavone synthase II; CHS: chalcone synthase; VORM: vitexin 2’’-O-rhamnoside 7-O-methyltransferase. The arrows in the figure represent enzymatic reactions. [file 12870_2024_4884_MOESM7_ESM.docx]

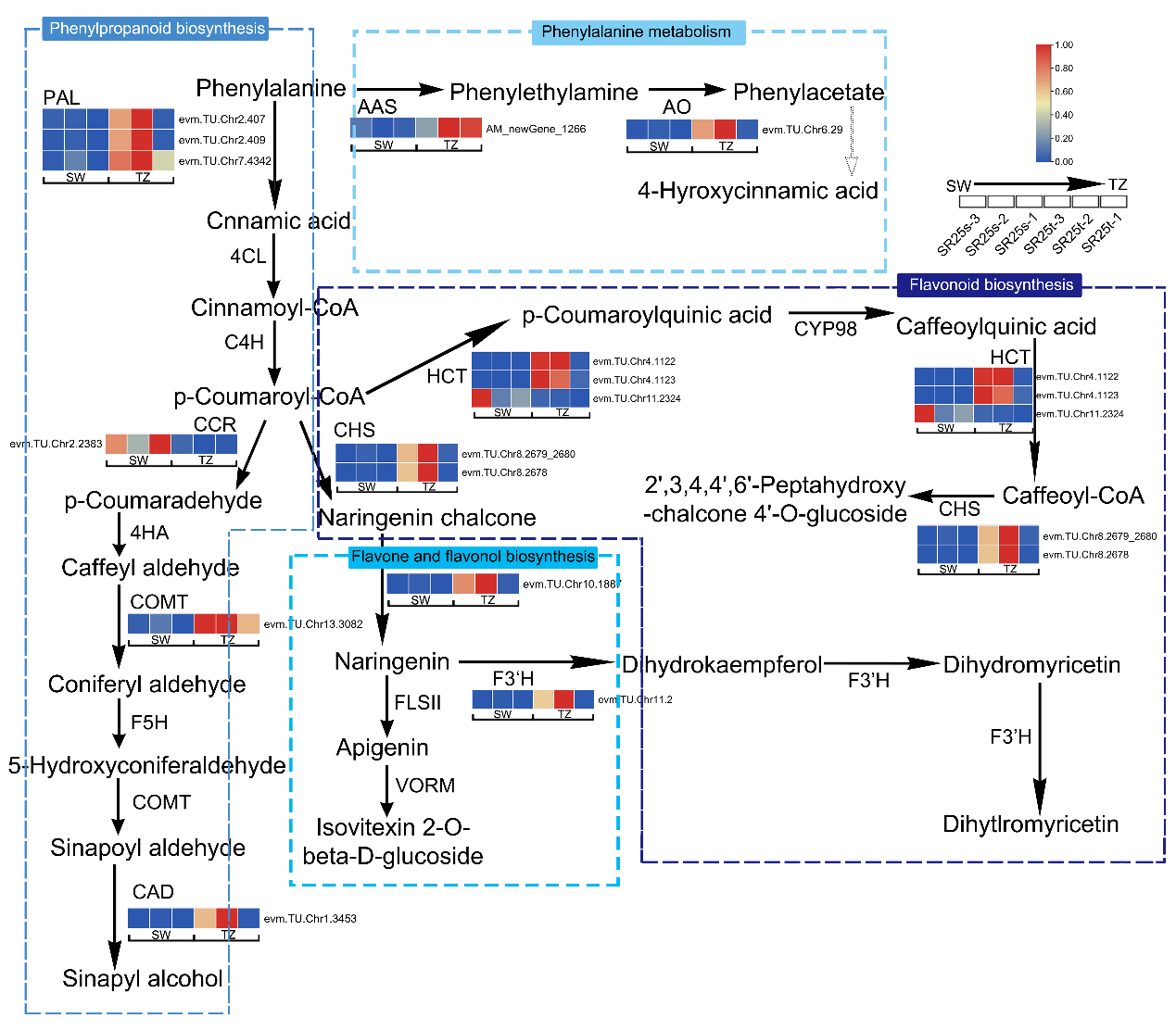
**Additional file 7:Figure S4.** Regulatory map of gene expression related to phenylalanine and flavonoid biosynthesis. PAL: phenylalanine ammonia-lyase; AAS: aromatic-L-amino-acid decarboxylase; AO: primary-amine oxidase; 4CL: Phenylalanine ammonia-lyase; C4H: trans-cinnamate 4-monooxygenase; CCR: cinnamoyl-CoA reductase; CHS: chalcone synthase; 4HA: 4-Hydroxycinnamyl aldehyde; F5H: ferulate-5-hydroxylase; COMT: caffeic acid 3-o-methyltransferase; CAD: cinnamyl-alcohol dehydrogenase; HCT: shikimate O-hydroxycinnamoyltransferase; CHI: chalcone isomerase; FLSII**:** flavone synthase II; CHS: chalcone synthase; VORM: vitexin 2''-O-rhamnoside 7-O-methyltransferase. The arrows in the figure represent enzymatic reactions.
