# Supplementary material for: Comparative physiological, biochemical, metabolomic, and transcriptomic analyses reveal the formation mechanism of heartwood for Acacia melanoxylon
Source: BMC Plant Biol. 2024 Apr 22;24:308. doi: 10.1186/s12870-024-04884-1 (PMC11034122; doi:10.1186/s12870-024-04884-1)
Supplement: Supplementary file 8 — Additional file 8: Table S4. Differential expressed metabolites identified in three groups. [file 12870_2024_4884_MOESM8_ESM.docx]

**Additional file 8:Table S4.** Differential expressed metabolites identified in three groups.

| DEM Set | up | down |
| --- | --- | --- |
| SR25HW vs. SR25SW | 2101 | 2271 |
| SR25TZ vs. SR25SW | 761 | 2314 |
| SR25HW vs SR25TZ | 2504 | 1186 |
