# Supplementary material for: Comparative physiological, biochemical, metabolomic, and transcriptomic analyses reveal the formation mechanism of heartwood for Acacia melanoxylon
Source: BMC Plant Biol. 2024 Apr 22;24:308. doi: 10.1186/s12870-024-04884-1 (PMC11034122; doi:10.1186/s12870-024-04884-1)
Supplement: Supplementary file 9 — Additional file 9: Figure S5. Pathway diagram of plant hormone signal transduction of A. melanoxylon. Note, the red box represents only the up-regulation sequence, and the blue box represents both up-regulated genes and down-regulated sequences. [file 12870_2024_4884_MOESM9_ESM.docx]

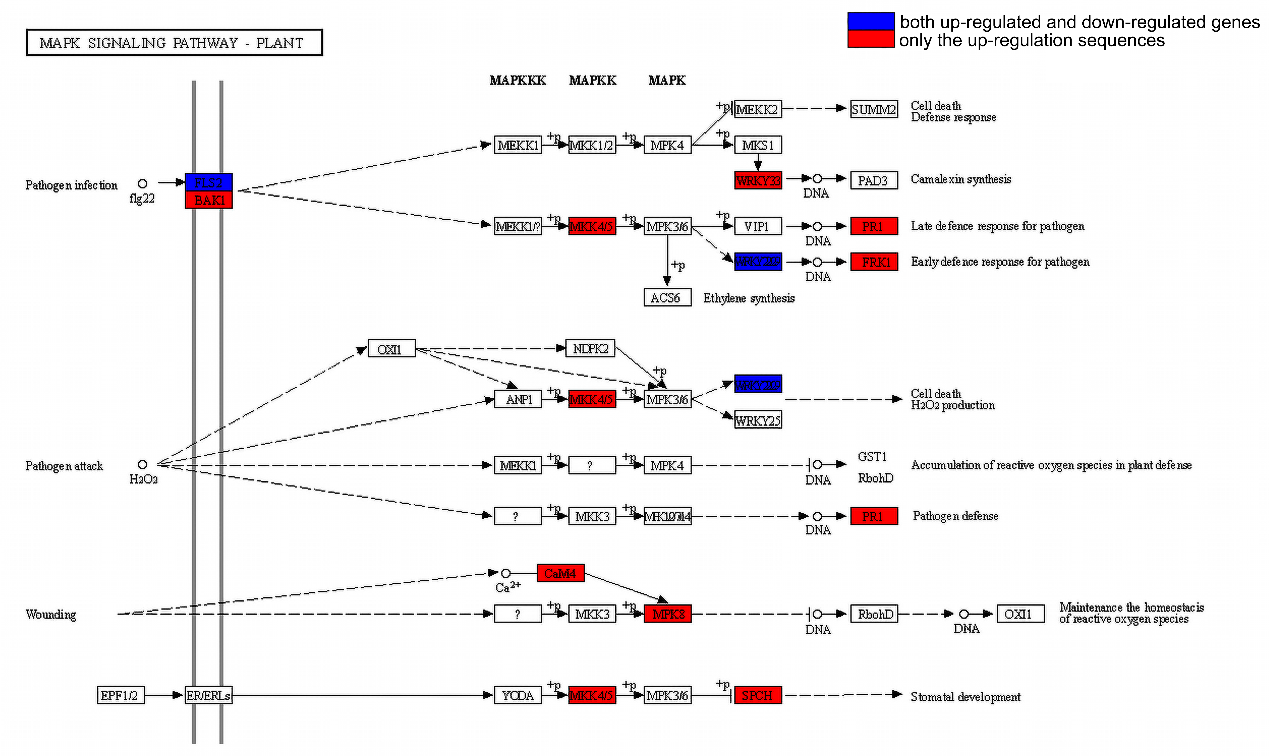
 **Additional file 9:FigureS5.** Pathway diagram of plant hormone signal transduction of *A. melanoxylon*. Note, the red box represents only the up-regulation sequence, and the blue box represents both up-regulated genes and down-regulated sequences.
