# Supplementary material for: Comparative physiological, biochemical, metabolomic, and transcriptomic analyses reveal the formation mechanism of heartwood for Acacia melanoxylon
Source: BMC Plant Biol. 2024 Apr 22;24:308. doi: 10.1186/s12870-024-04884-1 (PMC11034122; doi:10.1186/s12870-024-04884-1)
Supplement: Supplementary file 10 — Additional file 10: Table S5. KEGG enriched analysis of 34 metabolites for A. melanoxylon. [file 12870_2024_4884_MOESM10_ESM.docx]

**Additional file 10:Table S5.** KEGG enriched analysis of 34 metabolites for *A. melanoxylon*.

| pathway | Total | Hits | Raw p | -log(p) | Holm adjust | FDR | Impact | pathway |
| --- | --- | --- | --- | --- | --- | --- | --- | --- |
| ABC transporters | 138.00 | 20.00 | 0.00 | 8.76 | 0.02 | 0.00 | 0.14 | ath02010 |
| Phenylalanine metabolism | 60.00 | 12.00 | 0.00 | 8.70 | 0.02 | 0.00 | 0.26 | ath00360 |
| Aminoacyl-tRNA biosynthesis | 52.00 | 10.00 | 0.00 | 7.11 | 0.08 | 0.02 | 0.19 | ath00970 |
| Nicotinate and nicotinamide metabolism | 55.00 | 10.00 | 0.00 | 6.66 | 0.13 | 0.02 | 0.22 | ath00760 |
| Tyrosine metabolism | 78.00 | 12.00 | 0.00 | 6.22 | 0.20 | 0.03 | 0.22 | ath00350 |
| Monobactam biosynthesis | 45.00 | 8.00 | 0.00 | 5.40 | 0.45 | 0.06 | 0.18 | ath00261 |
| Linoleic acid metabolism | 28.00 | 6.00 | 0.01 | 5.25 | 0.52 | 0.06 | 0.34 | ath00591 |
| Citrate cycle (TCA cycle) | 20.00 | 5.00 | 0.01 | 5.22 | 0.52 | 0.06 | 0.13 | ath00020 |
| Phenylpropanoid biosynthesis | 68.00 | 10.00 | 0.01 | 5.04 | 0.62 | 0.06 | 0.19 | ath00940 |
| Lysine degradation | 50.00 | 8.00 | 0.01 | 4.75 | 0.82 | 0.07 | 0.18 | ath00310 |
| Flavone and flavonol biosynthesis | 51.00 | 8.00 | 0.01 | 4.63 | 0.92 | 0.07 | 0.15 | ath00944 |
| Carbon fixation in photosynthetic organisms | 23.00 | 5.00 | 0.01 | 4.60 | 0.94 | 0.07 | 0.16 | ath00710 |
| Arginine biosynthesis | 23.00 | 5.00 | 0.01 | 4.60 | 0.94 | 0.07 | 0.34 | ath00220 |
| Butanoate metabolism | 42.00 | 7.00 | 0.01 | 4.50 | 1.00 | 0.07 | 0.28 | ath00650 |
| Phenylalanine, tyrosine and tryptophan biosynthesis | 34.00 | 5.00 | 0.05 | 3.01 | 1.00 | 0.30 | 0.11 | ath00400 |
| Galactose metabolism | 46.00 | 6.00 | 0.05 | 2.92 | 1.00 | 0.30 | 0.14 | ath00052 |
| Lysine biosynthesis | 35.00 | 5.00 | 0.05 | 2.91 | 1.00 | 0.30 | 0.17 | ath00300 |
| Starch and sucrose metabolism | 37.00 | 5.00 | 0.07 | 2.71 | 1.00 | 0.35 | 0.19 | ath00500 |
| Glutathione metabolism | 38.00 | 5.00 | 0.07 | 2.61 | 1.00 | 0.37 | 0.19 | ath00480 |
| Pyruvate metabolism | 31.00 | 4.00 | 0.11 | 2.20 | 1.00 | 0.53 | 0.07 | ath00620 |
| beta-Alanine metabolism | 32.00 | 4.00 | 0.12 | 2.12 | 1.00 | 0.56 | 0.13 | ath00410 |
| Cyanoamino acid metabolism | 45.00 | 5.00 | 0.13 | 2.05 | 1.00 | 0.56 | 0.12 | ath00460 |
| Sulfur metabolism | 33.00 | 4.00 | 0.13 | 2.03 | 1.00 | 0.56 | 0.10 | ath00920 |
| Caffeine metabolism | 22.00 | 3.00 | 0.14 | 1.96 | 1.00 | 0.57 | 0.13 | ath00232 |
| Valine, leucine and isoleucine biosynthesis | 23.00 | 3.00 | 0.16 | 1.86 | 1.00 | 0.61 | 0.20 | ath00290 |
| Glyoxylate and dicarboxylate metabolism | 62.00 | 6.00 | 0.16 | 1.81 | 1.00 | 0.62 | 0.12 | ath00630 |
| Cysteine and methionine metabolism | 63.00 | 6.00 | 0.17 | 1.76 | 1.00 | 0.62 | 0.10 | ath00270 |
| Glycine, serine and threonine metabolism | 50.00 | 5.00 | 0.18 | 1.74 | 1.00 | 0.62 | 0.20 | ath00260 |
| Sphingolipid metabolism | 25.00 | 3.00 | 0.19 | 1.69 | 1.00 | 0.63 | 0.19 | ath00600 |
| Biosynthesis of various secondary metabolites - part 2 | 81.00 | 7.00 | 0.21 | 1.57 | 1.00 | 0.69 | 0.10 | ath00998 |
| Cutin, suberine and wax biosynthesis | 27.00 | 3.00 | 0.22 | 1.53 | 1.00 | 0.70 | 0.07 | ath00073 |
| Oxidative phosphorylation | 16.00 | 2.00 | 0.25 | 1.40 | 1.00 | 0.77 | 0.20 | ath00190 |
| Pantothenate and CoA biosynthesis | 30.00 | 3.00 | 0.27 | 1.33 | 1.00 | 0.80 | 0.13 | ath00770 |
| Synthesis and degradation of ketone bodies | 6.00 | 1.00 | 0.31 | 1.17 | 1.00 | 0.91 | 0.07 | ath00072 |
| Arginine and proline metabolism | 78.00 | 6.00 | 0.32 | 1.13 | 1.00 | 0.92 | 0.13 | ath00330 |
| C5-Branched dibasic acid metabolism | 34.00 | 3.00 | 0.33 | 1.10 | 1.00 | 0.93 | 0.10 | ath00660 |
| Ascorbate and aldarate metabolism | 50.00 | 4.00 | 0.35 | 1.05 | 1.00 | 0.95 | 0.17 | ath00053 |
| Anthocyanin biosynthesis | 66.00 | 5.00 | 0.36 | 1.02 | 1.00 | 0.95 | 0.10 | ath00942 |
| Taurine and hypotaurine metabolism | 22.00 | 2.00 | 0.38 | 0.96 | 1.00 | 0.99 | 0.14 | ath00430 |
| Fructose and mannose metabolism | 54.00 | 4.00 | 0.41 | 0.90 | 1.00 | 1.00 | 0.16 | ath00051 |
| Pentose and glucuronate interconversions | 56.00 | 4.00 | 0.43 | 0.84 | 1.00 | 1.00 | 0.10 | ath00040 |
| Plant hormone signal transduction | 12.00 | 1.00 | 0.52 | 0.65 | 1.00 | 1.00 | 0.08 | ath04075 |
| Vitamin B6 metabolism | 29.00 | 2.00 | 0.52 | 0.65 | 1.00 | 1.00 | 0.10 | ath00750 |
| Histidine metabolism | 47.00 | 3.00 | 0.54 | 0.62 | 1.00 | 1.00 | 0.04 | ath00340 |
| Glycolysis / Gluconeogenesis | 31.00 | 2.00 | 0.56 | 0.58 | 1.00 | 1.00 | 0.07 | ath00010 |
| Tropane, piperidine and pyridine alkaloid biosynthesis | 68.00 | 4.00 | 0.59 | 0.53 | 1.00 | 1.00 | 0.07 | ath00960 |
| Pentose phosphate pathway | 35.00 | 2.00 | 0.63 | 0.47 | 1.00 | 1.00 | 0.14 | ath00030 |
| Nitrogen metabolism | 19.00 | 1.00 | 0.69 | 0.37 | 1.00 | 1.00 | 0.08 | ath00910 |
| Riboflavin metabolism | 20.00 | 1.00 | 0.71 | 0.34 | 1.00 | 1.00 | 0.01 | ath00740 |
| alpha-Linolenic acid metabolism | 44.00 | 2.00 | 0.75 | 0.29 | 1.00 | 1.00 | 0.02 | ath00592 |
| Betalain biosynthesis | 24.00 | 1.00 | 0.77 | 0.26 | 1.00 | 1.00 | 0.04 | ath00965 |
| Inositol phosphate metabolism | 47.00 | 2.00 | 0.78 | 0.25 | 1.00 | 1.00 | 0.01 | ath00562 |
| Amino sugar and nucleotide sugar metabolism | 108.00 | 5.00 | 0.78 | 0.24 | 1.00 | 1.00 | 0.05 | ath00520 |
| Propanoate metabolism | 48.00 | 2.00 | 0.79 | 0.23 | 1.00 | 1.00 | 0.07 | ath00640 |
| Ubiquinone and other terpenoid-quinone biosynthesis | 92.00 | 4.00 | 0.81 | 0.21 | 1.00 | 1.00 | 0.14 | ath00130 |
| Biotin metabolism | 28.00 | 1.00 | 0.82 | 0.19 | 1.00 | 1.00 | 0.01 | ath00780 |
| Glycerophospholipid metabolism | 52.00 | 2.00 | 0.83 | 0.19 | 1.00 | 1.00 | 0.05 | ath00564 |
| Purine metabolism | 95.00 | 4.00 | 0.83 | 0.19 | 1.00 | 1.00 | 0.02 | ath00230 |
| Glucosinolate biosynthesis | 77.00 | 3.00 | 0.85 | 0.16 | 1.00 | 1.00 | 0.02 | ath00966 |
| Thiamine metabolism | 31.00 | 1.00 | 0.85 | 0.16 | 1.00 | 1.00 | 0.01 | ath00730 |
| Folate biosynthesis | 58.00 | 2.00 | 0.87 | 0.14 | 1.00 | 1.00 | 0.03 | ath00790 |
| Tryptophan metabolism | 83.00 | 3.00 | 0.88 | 0.13 | 1.00 | 1.00 | 0.04 | ath00380 |
| Zeatin biosynthesis | 39.00 | 1.00 | 0.91 | 0.09 | 1.00 | 1.00 | 0.01 | ath00908 |
| Terpenoid backbone biosynthesis | 45.00 | 1.00 | 0.94 | 0.06 | 1.00 | 1.00 | 0.04 | ath00900 |
| Biosynthesis of unsaturated fatty acids | 74.00 | 2.00 | 0.94 | 0.06 | 1.00 | 1.00 | 0.04 | ath01040 |
| Arachidonic acid metabolism | 75.00 | 2.00 | 0.94 | 0.06 | 1.00 | 1.00 | 0.02 | ath00590 |
| Fatty acid degradation | 50.00 | 1.00 | 0.96 | 0.05 | 1.00 | 1.00 | 0.00 | ath00071 |
| Carotenoid biosynthesis | 115.00 | 3.00 | 0.97 | 0.03 | 1.00 | 1.00 | 0.03 | ath00906 |
| Fatty acid biosynthesis | 58.00 | 1.00 | 0.97 | 0.03 | 1.00 | 1.00 | 0.01 | ath00061 |
| Isoquinoline alkaloid biosynthesis | 122.00 | 3.00 | 0.98 | 0.02 | 1.00 | 1.00 | 0.06 | ath00950 |
| Pyrimidine metabolism | 65.00 | 1.00 | 0.98 | 0.02 | 1.00 | 1.00 | 0.05 | ath00240 |
| Indole alkaloid biosynthesis | 70.00 | 1.00 | 0.99 | 0.01 | 1.00 | 1.00 | 0.05 | ath00901 |
| Porphyrin and chlorophyll metabolism | 142.00 | 3.00 | 0.99 | 0.01 | 1.00 | 1.00 | 0.01 | ath00860 |
| Diterpenoid biosynthesis | 122.00 | 1.00 | 1.00 | 0.00 | 1.00 | 1.00 | 0.03 | ath00904 |
| Flavonoid biosynthesis | 74.00 | 15.00 | 0.00 | 10.76 | 0.00 | 0.00 | 0.20 | ath00941 |
| Alanine, aspartate and glutamate metabolism | 28.00 | 10.00 | 0.00 | 12.94 | 0.00 | 0.00 | 0.52 | ath00250 |
