# Supplementary material for: Comparative physiological, biochemical, metabolomic, and transcriptomic analyses reveal the formation mechanism of heartwood for Acacia melanoxylon
Source: BMC Plant Biol. 2024 Apr 22;24:308. doi: 10.1186/s12870-024-04884-1 (PMC11034122; doi:10.1186/s12870-024-04884-1)
Supplement: Supplementary file 11 — Additional file 11: Figure S6. Pathway diagram of MAPK signal (plant) of A. melanoxylon. Note, the red box represents only the up-regulation sequence, the green box represents only the down-regulation sequence, and the blue box represents both up-regulated genes and down-regulated sequences. The dotted box is framed to describe the object. [file 12870_2024_4884_MOESM11_ESM.docx]

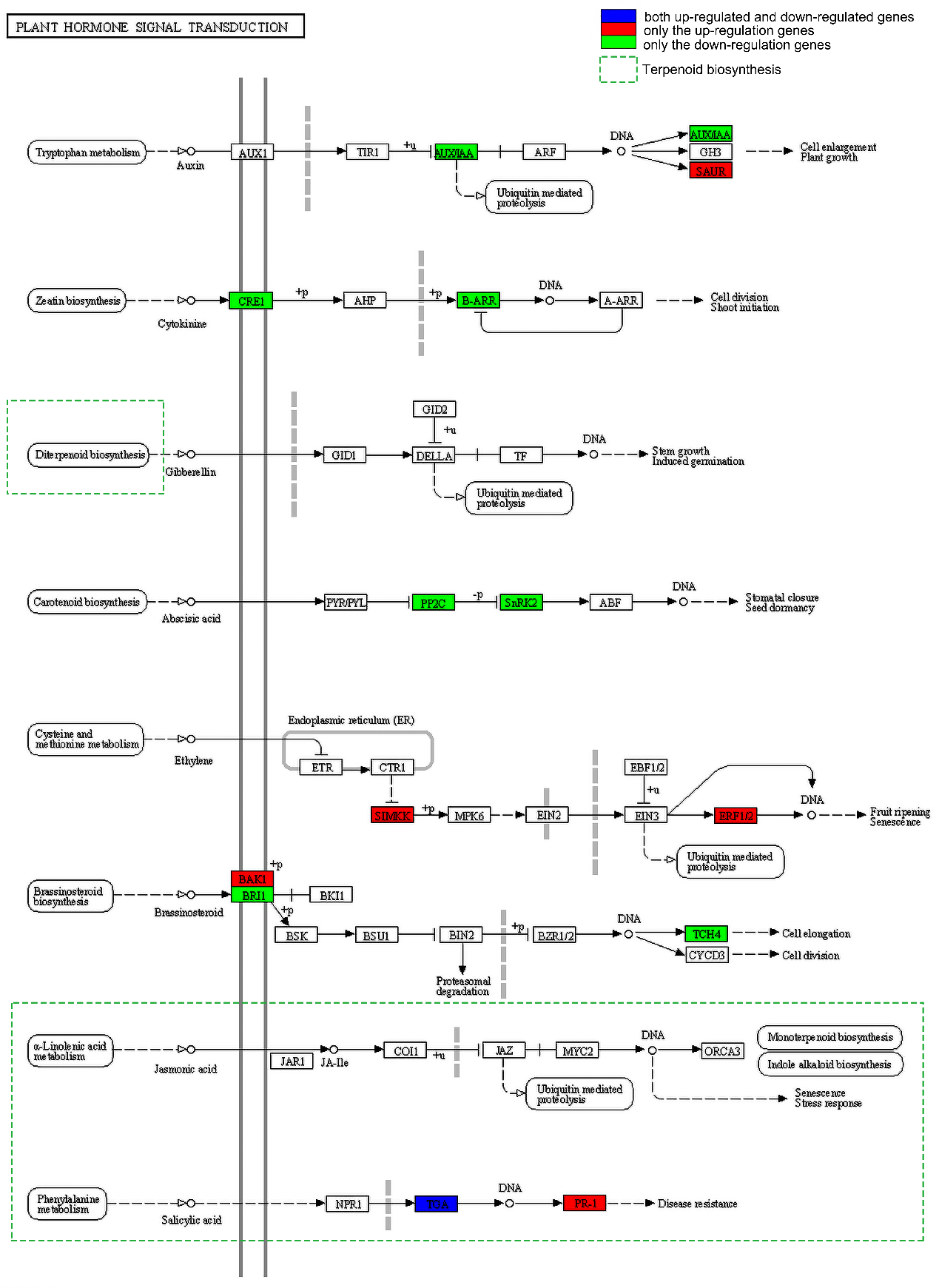
 **Additional File 11:figureS6.** Pathway diagram of MAPK signal (plant) of *A. melanoxylon*. Note, the red box represents only the up-regulation sequence, the green box represents only the down-regulation sequence, and the blue box represents both up-regulated genes and down-regulated sequences. The dotted box is framed to describe the object.
