# Supplementary material for: Comparative physiological, biochemical, metabolomic, and transcriptomic analyses reveal the formation mechanism of heartwood for Acacia melanoxylon
Source: BMC Plant Biol. 2024 Apr 22;24:308. doi: 10.1186/s12870-024-04884-1 (PMC11034122; doi:10.1186/s12870-024-04884-1)
Supplement: Supplementary file 12 — Additional file 12: Table S6. Statistics of differential expressed genes in SR25SW vs. SR25TZ. [file 12870_2024_4884_MOESM12_ESM.docx]

**Additional file 12:Table S6.** Statistics of differential expressed genes in SR25SW vs. SR25TZ.

| DEG Set | DEG Number | up-regulated | down-regulated |
| --- | --- | --- | --- |
| SR25SW vs. SR25TZ | 700 | 396 | 304 |
