# Supplementary material for: Comparative physiological, biochemical, metabolomic, and transcriptomic analyses reveal the formation mechanism of heartwood for Acacia melanoxylon
Source: BMC Plant Biol. 2024 Apr 22;24:308. doi: 10.1186/s12870-024-04884-1 (PMC11034122; doi:10.1186/s12870-024-04884-1)
Supplement: Supplementary file 13 — Additional file 13: Table S7. Differential expressed genes related to plant hormones in SR25SW vs. SR25TZ. [file 12870_2024_4884_MOESM13_ESM.docx]

**Additional file 13: Table S7.** Differential expressed genes related to plant hormones in SR25SW vs. SR25TZ.

| Gene family | GeneID | SR25s-3 | SR25s-2 | SR25s-1 | SR25t-3 | SR25t-2 | SR25t-1 | FDR | log2FC | FC |
| --- | --- | --- | --- | --- | --- | --- | --- | --- | --- | --- |
| PAL | evm.TU.Chr2.407 | 2.74 | 2.24 | 0.34 | 46.01 | 65.63 | 1.85 | 0.01 | 4.35 | 20.45 |
| PAL | evm.TU.Chr2.409 | 0.85 | 1.56 | 0.10 | 35.28 | 50.65 | 1.26 | 0.00 | 4.94 | 30.65 |
| PAL | evm.TU.Chr7.4342 | 20.44 | 58.78 | 14.25 | 266.38 | 323.75 | 146.21 | 0.01 | 2.85 | 7.20 |
| CCR | evm.TU.Chr2.2383 | 102.07 | 48.31 | 133.45 | 16.48 | 9.33 | 11.16 | 0.00 | (2.79) | 0.14 |
| COMT | evm.TU.Chr13.3082 | 23.46 | 72.47 | 8.85 | 538.75 | 541.04 | 349.70 | 0.00 | 3.62 | 12.31 |
| CAD | evm.TU.Chr1.3453 | 0.02 | 0.14 | 0.04 | 5.87 | 9.33 | 0.12 | 0.00 | 5.99 | 63.77 |
| AAS | AM_newGene_1266 | 2.24 | 0.45 | 1.00 | 4.54 | 16.98 | 15.99 | 0.00 | 3.56 | 11.76 |
| AO | evm.TU.Chr6.29 | 0.38 | 1.27 | 0.11 | 36.89 | 52.37 | 1.53 | 0.00 | 5.44 | 43.42 |
| HCT | evm.TU.Chr4.1122 | 0.00 | 0.00 | 0.00 | 1.62 | 1.65 | 0.03 | 0.00 | 8.44 | 346.98 |
| HCT | evm.TU.Chr4.1123 | 0.00 | 0.00 | 0.00 | 1.95 | 1.64 | 0.12 | 0.00 | 8.64 | 400.24 |
| HCT | evm.TU.Chr11.2324 | 101.29 | 15.08 | 26.08 | 6.71 | 4.60 | 1.57 | 0.00 | (3.30) | 0.10 |
| CHS | evm.TU.Chr8.2679_2680 | 3.42 | 51.45 | 5.04 | 808.12 | 1279.06 | 54.55 | 0.01 | 4.85 | 28.90 |
| CHS | evm.TU.Chr8.2678 | 2.24 | 17.36 | 1.06 | 753.56 | 1243.35 | 18.03 | 0.00 | 6.31 | 79.26 |
| F3'H | evm.TU.Chr11.2 | 4.19 | 77.93 | 4.23 | 3001.66 | 5179.34 | 47.90 | 0.00 | 6.24 | 75.76 |
| CHI | evm.TU.Chr10.1887 | 4.05 | 28.91 | 1.63 | 885.33 | 1189.46 | 31.03 | 0.00 | 5.63 | 49.42 |
| ATS | evm.TU.Chr12.1822 | 36.22 | 0.00 | 0.00 | 0.05 | 0.00 | 1584.99 | 0.95 | 3.15 | 8.90 |
| ATS | evm.TU.Chr2.509 | 0.85 | 2.38 | 0.73 | 0.11 | 0.08 | 16.99 | 0.99 | 0.63 | 1.55 |
| ATS | evm.TU.Chr2.510 | 0.23 | 0.00 | 0.00 | 0.00 | 0.12 | 0.29 | 1.00 | 0.13 | 1.10 |
| FDF | evm.TU.Chr10.2974 | 72843.78 | 64112.27 | 75701.38 | 69807.96 | 64888.98 | 83140.54 | 1.00 | 0.03 | 1.02 |
| SME | evm.TU.Chr11.369 | 980.26 | 62.31 | 554.86 | 1172.96 | 1171.96 | 159.01 | 0.96 | 0.40 | 1.32 |
| SME | evm.TU.Chr11.368 | 336.23 | 41.01 | 216.01 | 731.08 | 581.09 | 71.51 | 0.93 | 0.61 | 1.52 |
| SME | evm.TU.Chr1.2348 | 0.37 | 0.00 | 0.00 | 0.00 | 0.00 | 33.10 | 0.91 | 3.66 | 12.67 |
| GES | Acacia_melanoxylon_newGene_10347 | 2.18 | 0.80 | 0.46 | 269.46 | 1323.74 | 8.34 | 0.00 | 4.06 | 16.63 |
| TTS | evm.TU.Chr7.1770 | 983.08 | 1659.34 | 3963.08 | 2578.59 | 2131.47 | 1515.80 | 1.00 | 0.02 | 1.01 |
| AACT | evm.TU.Chr1.782 | 3023.09 | 2340.21 | 1421.61 | 4103.32 | 4460.64 | 3598.73 | 0.95 | 0.33 | 1.25 |
| AACT | evm.TU.Chr4.1400 | 12052.27 | 2936.02 | 9315.09 | 10710.02 | 11620.81 | 1662.41 | 0.98 | 0.22 | 1.16 |
| AACT | evm.TU.Chr5.3110 | 31053.51 | 24103.19 | 75551.54 | 48764.77 | 43161.70 | 19224.46 | 1.00 | (0.09) | 0.94 |
| HMGS | evm.TU.Chr3.2225 | 18882.28 | 4382.64 | 9695.16 | 10859.86 | 6181.30 | 54635.92 | 0.93 | 0.62 | 1.54 |
| HMGS | evm.TU.Chr7.2829 | 3609.10 | 10943.27 | 7362.12 | 305917.35 | 457556.87 | 3217.14 | 0.11 | 2.23 | 4.70 |
| HMGR | evm.TU.Chr3.3068 | 954.00 | 264.83 | 367.72 | 175.10 | 200.72 | 1411.58 | 1.00 | 0.12 | 1.09 |
| MK | evm.TU.Chr7.1743 | 0.00 | 0.02 | 0.00 | 0.00 | 0.00 | 0.23 | 0.98 | 1.69 | 3.23 |
| PMK | evm.TU.Chr3.1791 | 1475.89 | 5099.44 | 205.22 | 30327.72 | 47160.52 | 5636.64 | 0.20 | 1.79 | 3.45 |
| PMK | evm.TU.Chr11.2255 | 46441.29 | 40277.93 | 13829.88 | 41241.35 | 38828.47 | 39555.06 | 0.99 | 0.18 | 1.13 |
| MVD | evm.TU.Chr7.4497 | 20786.23 | 25137.84 | 30287.36 | 52270.49 | 71358.80 | 21330.53 | 0.85 | 0.41 | 1.33 |
| GGDS | evm.TU.Chr1.1067 | 21838.64 | 13264.84 | 24250.06 | 10797.88 | 16024.16 | 24507.49 | 1.00 | (0.04) | 0.97 |
| GGDS | evm.TU.Chr11.117 | 13543.43 | 102455.95 | 11506.95 | 215738.51 | 143574.99 | 37672.02 | 0.85 | 0.80 | 1.74 |
| GGDS | evm.TU.Chr11.582 | 3181.07 | 31057.01 | 1831.19 | 101855.38 | 101154.88 | 58648.38 | 0.29 | 1.57 | 2.96 |
| GGDS | evm.TU.Chr4.1885 | 0.67 | 0.08 | 0.40 | 0.06 | 0.16 | 5.43 | 0.96 | 1.08 | 2.11 |
| GGDS | evm.TU.Chr11.269 | 2572.72 | 2691.92 | 4805.83 | 13294.29 | 15787.79 | 1839.25 | 0.72 | 0.68 | 1.60 |
| GGDS | evm.TU.Chr3.2662 | 6414.71 | 4038.83 | 12099.70 | 9937.48 | 8317.15 | 3478.19 | 1.00 | (0.01) | 0.99 |
| GGDS | Acacia_melanoxylon_newGene_11870 | 3710.05 | 443.76 | 1840.14 | 3896.56 | 2327.35 | 273.81 | 1.00 | 0.03 | 1.02 |
| GGDS | evm.TU.Chr1.801 | 1649.48 | 2020.41 | 805.17 | 7121.70 | 6095.80 | 1508.55 | 0.61 | 0.77 | 1.70 |
| GGDS | evm.TU.Chr1.912 | 3170.69 | 4748.61 | 876.75 | 7006.86 | 6579.98 | 2571.00 | 0.95 | 0.43 | 1.34 |
| GGDS | evm.TU.Chr5.2804 | 12.20 | 43.83 | 8.03 | 22.68 | 20.61 | 107.81 | 0.96 | 0.63 | 1.54 |
| SuS | AM_new12677 | 10.51 | 0.18 | 0.03 | 3190.09 | 16546.65 | 3.82 | 5.59 | 48.25 | ####### |
| SuS | evm.Chr10.1547 | 174.90 | 9.32 | 0.91 | 30779.04 | 94163.89 | 29.05 | 4.80 | 27.88 | ####### |
| SuS | AM_new1651 | 0.02 | 0.00 | 0.00 | 9.07 | 47.89 | 3.41 | 6.40 | 84.68 | ####### |
| UGP | evm.Chr10.3447 | 5416.20 | 8499.82 | 14595.94 | 15251.74 | 11099.37 | 5394.11 | (0.20) | 0.87 | 1.83 |
| APS | evm.Chr1.1473 | 0.00 | 0.00 | 0.00 | 0.14 | 0.09 | 0.00 | 2.99 | 7.94 | 244.97 |
| APS | evm.Chr1.230 | 0.37 | 0.17 | 1.90 | 0.00 | 2.78 | 15.71 | 1.44 | 2.72 | 6.58 |
| APS | evm.Chr2.2449 | 4189.87 | 83.09 | 894.03 | 9065.69 | 11238.30 | 364.30 | 1.11 | 2.16 | 4.48 |
| SS | evm.Chr1.1011 | 2.82 | 0.00 | 0.70 | 23.38 | 22.17 | 0.57 | 2.00 | 4.01 | 16.15 |
| SS | evm.Chr1.1014 | 0.00 | 0.20 | 2.56 | 21.41 | 7.24 | 0.00 | 1.66 | 3.17 | 9.01 |
| SS | evm.Chr5.1836 | 0.84 | 0.01 | 0.00 | 0.00 | 0.00 | 15.79 | 2.20 | 4.58 | 23.96 |
| SS | evm.Chr5.1837 | 0.00 | 0.12 | 0.00 | 0.00 | 0.00 | 1.33 | 1.73 | 3.31 | 9.93 |
| SBE | evm.Chr1.3116 | 687.12 | 527.76 | 2044.56 | 1844.59 | 1119.34 | 1039.31 | (0.09) | 0.94 | 1.92 |
| DBE | evm.Chr10.4545 | 708.77 | 1575.32 | 1225.20 | 1002.73 | 1279.26 | 1395.42 | (0.22) | 0.86 | 1.81 |
| DBE | evm.Chr7.4696 | 1721.11 | 2151.09 | 4897.31 | 5732.72 | 4615.08 | 919.95 | (0.29) | 0.82 | 1.76 |
| AMY,BAM | evm.Chr4.2380 | 2.07 | 1.45 | 0.00 | 190497.96 | 6119.78 | 38.90 | 7.43 | 172.10 | ####### |
| AMY,BAM | evm.Chr12.1760 | 0.75 | 32.19 | 0.69 | 660.62 | 8870.65 | 955.09 | 4.17 | 18.04 | ####### |
| AMY,BAM | evm.Chr5.658 | 101.46 | 18.44 | 14.26 | 2357.78 | 3796.03 | 502.80 | 2.86 | 7.27 | 154.49 |
| PGI | evm.Chr4.2969 | 74598.78 | 140359.59 | 83370.90 | 406263.42 | 355922.97 | 53008.65 | 0.57 | 1.48 | 2.80 |
| PGI | evm.Chr7.1974 | 61320.76 | 128523.69 | 99446.16 | 325334.63 | 659228.75 | 85289.82 | 0.80 | 1.75 | 3.35 |
| PGM | evm.Chr11.2522 | 39038.35 | 35902.25 | 56533.31 | 55889.81 | 64903.12 | 23736.39 | 0.04 | 1.03 | 2.04 |
| PGM | evm.Chr12.1471 | 28927.65 | 37613.06 | 35314.97 | 62138.67 | 58324.43 | 11852.46 | 0.07 | 1.05 | 2.07 |
| USP,UGP | evm.Chr2.3266 | 2386.72 | 176.44 | 319.65 | 6339.83 | 5797.44 | 655.60 | 1.20 | 2.29 | 4.90 |
| USP,UGP | AM_new11334 | 3.84 | 0.58 | 0.00 | 0.73 | 4.65 | 4.70 | 1.03 | 2.05 | 4.13 |
| USP,UGP | AM_new12771 | 0.00 | 0.00 | 0.00 | 0.05 | 0.00 | 0.18 | 4.12 | 17.38 | ####### |
| USP,UGP | evm.Chr2.2249 | 973022.34 | 967894.28 | 590225.92 | 2049545.17 | 2574542.01 | 878596.20 | 0.52 | 1.43 | 2.69 |
| UGD | evm.Chr3.870 | 582594.26 | 53567.35 | 68477.37 | 518795.46 | 282222.09 | 289948.88 | 0.58 | 1.50 | 2.83 |
| UGD | evm.Chr6.1620 | 104.59 | 65.50 | 60.61 | 2300.33 | 5509.98 | 44.28 | 2.23 | 4.68 | 25.68 |
| UGE | evm.Chr11.815 | 0.00 | 0.05 | 0.36 | 0.05 | 0.41 | 0.00 | 0.05 | 1.04 | 2.05 |
| UGE | evm.Chr3.1983 | 31570.41 | 10528.95 | 20006.81 | 56512.59 | 49200.09 | 17741.86 | 0.09 | 1.06 | 2.09 |
| UXS6,AXS2 | evm.Chr2.3079 | 52369.64 | 158545.92 | 29914.11 | 101237.53 | 97010.35 | 109180.25 | 0.23 | 1.18 | 2.26 |
| UXS6,AXS2 | evm.Chr5.2547 | 0.00 | 0.02 | 0.01 | 0.00 | 0.16 | 0.20 | 1.02 | 2.02 | 4.06 |
| UXS6,AXS2 | evm.Chr7.1548_7.1549 | 52011.05 | 28929.19 | 42254.74 | 96667.04 | 112593.85 | 19835.85 | 0.36 | 1.29 | 2.44 |
| CSLD | evm.Chr5.3666 | 99788.68 | 161469.06 | 89550.18 | 150315.22 | 138514.72 | 84110.10 | 0.02 | 1.02 | 2.02 |
| XYL | evm.Chr11.2178 | 228.28 | 97.07 | 280.67 | 468.26 | 536.47 | 117.88 | 0.42 | 1.34 | 2.52 |
| XYL | evm.Chr8.2247 | 15386.82 | 5350.72 | 826.20 | 553869.66 | 491135.39 | 257.14 | 2.60 | 6.05 | 66.11 |
| XK | evm.Chr1.1569 | 4658.75 | 2451.98 | 4565.89 | 7876.83 | 9310.46 | 2080.79 | 0.31 | 1.24 | 2.36 |
| XK | evm.Chr2.1596 | 0.19 | 0.00 | 0.00 | 0.46 | 0.05 | 0.00 | 1.07 | 2.10 | 4.29 |
| XK | evm.Chr2.3012 | 0.05 | 0.00 | 0.00 | 787.74 | 1450.92 | 1.18 | 8.13 | 280.51 | ####### |
| XI | evm.Chr4.2910 | 10.35 | 2.40 | 0.84 | 5.20 | 6.51 | 1.21 | 0.07 | 1.05 | 2.07 |
| XI | evm.Chr4.2911 | 2688.94 | 3302.85 | 3487.58 | 5463.83 | 8865.13 | 2482.66 | 0.35 | 1.28 | 2.42 |
| RPE | evm.Chr7.688 | 540.75 | 332.08 | 1958.51 | 2597.86 | 1876.05 | 261.63 | (0.06) | 0.96 | 1.94 |
| RSW | evm.Chr11.1140 | 2241.99 | 131.99 | 88.91 | 1003.30 | 1046.21 | 543.47 | 0.43 | 1.35 | 2.54 |
| RSW | evm.Chr4.79 | 0.00 | 0.00 | 0.00 | 0.00 | 0.10 | 0.16 | 2.90 | 7.47 | 177.22 |
| RSW | evm.Chr5.86 | 3472.32 | 6776.10 | 4506.12 | 30039.06 | 13646.64 | 3824.19 | 0.68 | 1.61 | 3.05 |
| TSL | evm.Chr5.3541 | 0.00 | 0.00 | 0.00 | 0.00 | 0.00 | 0.57 | 3.40 | 10.56 | 1510.14 |
| TSL | evm.Chr8.2087 | 33133.87 | 109521.59 | 38367.01 | 442628.94 | 623454.47 | 98299.97 | 1.23 | 2.35 | 5.10 |
| TSL | evm.Chr9.1338 | 282573.52 | 383866.83 | 220243.54 | 1737542.75 | 1522110.74 | 65994.30 | 0.70 | 1.62 | 3.08 |
