# Supplementary material for: Comparative physiological, biochemical, metabolomic, and transcriptomic analyses reveal the formation mechanism of heartwood for Acacia melanoxylon
Source: BMC Plant Biol. 2024 Apr 22;24:308. doi: 10.1186/s12870-024-04884-1 (PMC11034122; doi:10.1186/s12870-024-04884-1)
Supplement: Supplementary file 14 — Additional file 14: Table S8. Connection network between TFs and metabolites related to phenylpropanoids and flavonoids. [file 12870_2024_4884_MOESM14_ESM.docx]

**Additional file 14: Table S8.** Connection network between TFs and metabolites related to phenylpropanoids and flavonoids.

| TFs | GeneID | SR25s-3_Count | SR25s-2_Count | SR25s-1_Count | SR25t-3_Count | SR25t-2_Count | SR25t-1_Count | SR25s-3_FPKM | SR25s-2_FPKM | SR25s-1_FPKM | SR25t-3_FPKM | SR25t-2_FPKM | SR25t-1_FPKM | FDR | log2FC |
| --- | --- | --- | --- | --- | --- | --- | --- | --- | --- | --- | --- | --- | --- | --- | --- |
| Am-WRKY-1 | evm.TU.Chr2.1563 | 176.00 | 341.00 | 49.00 | 1434.00 | 2162.00 | 799.00 | 3.06 | 4.28 | 0.79 | 19.87 | 28.76 | 11.53 | 0.01 | 2.82 |
| Am-WRKY-2 | evm.TU.Chr5.287 | 22.00 | 4.00 | 2.00 | 483.00 | 385.00 | 14.00 | 0.28 | 0.03 | 0.02 | 4.88 | 3.73 | 0.15 | 0.01 | 4.82 |
| Am-WRKY-3 | evm.TU.Chr7.346 | 117.00 | 45.00 | 10.00 | 1784.00 | 3279.00 | 59.00 | 2.02 | 0.56 | 0.15 | 24.60 | 43.42 | 0.84 | 0.00 | 4.73 |
| Am-WRKY-4 | Am_newGene_5698 | 0.00 | 0.00 | 0.00 | 56.00 | 161.00 | 33.00 | 0.00 | 0.00 | 0.00 | 1.10 | 3.05 | 0.67 | 0.00 | 8.71 |
| Am-WRKY-5 | evm.TU.Chr9.207 | 2678.00 | 836.00 | 2030.00 | 321.00 | 338.00 | 175.00 | 51.68 | 11.59 | 36.58 | 4.89 | 4.96 | 2.77 | 0.00 | (2.80) |
| Am-WRKY-6 | evm.TU.Chr3.2132 | 14.00 | 11.00 | 0.00 | 709.00 | 1153.00 | 22.00 | 0.44 | 0.26 | 0.00 | 18.86 | 29.45 | 0.60 | 0.00 | 6.05 |
| Am-WRKY-7 | evm.TU.Chr4.2416 | 93.00 | 179.00 | 28.00 | 1268.00 | 1562.00 | 356.00 | 1.42 | 1.99 | 0.40 | 15.51 | 18.34 | 4.53 | 0.00 | 3.24 |
| Am-WRKY-8 | evm.TU.Chr6.2312 | 23.00 | 19.00 | 20.00 | 805.00 | 1161.00 | 18.00 | 0.48 | 0.28 | 0.40 | 13.74 | 19.03 | 0.32 | 0.00 | 4.82 |
| Am-WRKY-9 | evm.TU.Chr13.3000 | 10.00 | 47.00 | 6.00 | 547.00 | 462.00 | 129.00 | 0.14 | 0.52 | 0.08 | 6.75 | 5.48 | 1.66 | 0.00 | 3.98 |
| Am-WRKY-10 | evm.TU.Chr6.2098 | 14.00 | 12.00 | 6.00 | 42.00 | 109.00 | 237.00 | 0.75 | 0.45 | 0.27 | 1.76 | 4.44 | 10.50 | 0.00 | 3.68 |
| Am-MYB-1 | evm.TU.Chr6.1985 | 418.00 | 158.00 | 207.00 | 1102.00 | 1513.00 | 701.00 | 9.51 | 2.58 | 4.39 | 19.91 | 26.24 | 13.19 | 0.00 | 2.01 |
| Am-MYB-2 | evm.TU.Chr12.2587 | 63.00 | 72.00 | 16.00 | 717.00 | 485.00 | 126.00 | 1.26 | 1.03 | 0.30 | 11.39 | 7.39 | 2.09 | 0.01 | 2.97 |
| Am-MYB-3 | evm.TU.Chr7.962 | 871.00 | 897.00 | 791.00 | 277.00 | 235.00 | 337.00 | 22.43 | 16.61 | 19.02 | 5.65 | 4.60 | 7.16 | 0.00 | (1.61) |
| Am-MYB-4 | evm.TU.Chr4.2517 | 527.00 | 40.00 | 249.00 | 6.00 | 7.00 | 8.00 | 19.56 | 1.06 | 8.62 | 0.16 | 0.20 | 0.24 | 0.00 | (5.26) |
| Am-MYB-5 | evm.TU.Chr8.627 | 376.00 | 199.00 | 159.00 | 1597.00 | 912.00 | 684.00 | 9.81 | 3.73 | 3.86 | 33.04 | 18.11 | 14.75 | 0.00 | 2.03 |
| Am-MYB-6 | evm.TU.Chr7.1978 | 161.00 | 13.00 | 29.00 | 5.00 | 2.00 | 2.00 | 3.79 | 0.21 | 0.63 | 0.08 | 0.03 | 0.04 | 0.01 | (4.54) |
| Am-MYB-7 | evm.TU.Chr11.382 | 4.00 | 14.00 | 4.00 | 92.00 | 71.00 | 42.00 | 0.10 | 0.26 | 0.10 | 2.02 | 1.51 | 0.96 | 0.01 | 3.09 |
| Am-MYB-8 | evm.TU.Chr13.3138 | 59.00 | 360.00 | 78.00 | 12390.00 | 13528.00 | 997.00 | 1.49 | 6.60 | 1.86 | 250.57 | 262.70 | 21.01 | 0.00 | 5.53 |
| Am-MYB-9 | evm.TU.Chr7.339 | 1124.00 | 1764.00 | 2607.00 | 492.00 | 271.00 | 165.00 | 14.95 | 16.87 | 32.37 | 5.18 | 2.73 | 1.81 | 0.00 | (2.68) |
| Am-bHLH1 | evm.TU.Chr11.1503 | 298.00 | 205.00 | 142.00 | 15.00 | 21.00 | 24.00 | 5.87 | 2.90 | 2.61 | 0.22 | 0.31 | 0.39 | 0.00 | (3.44) |
| Am-bHLH2 | evm.TU.Chr6.1063 | 2.00 | 19.00 | 15.00 | 238.00 | 90.00 | 148.00 | 0.03 | 0.22 | 0.23 | 3.13 | 1.13 | 2.02 | 0.00 | 3.64 |
| Am-bHLH3 | evm.TU.Chr2.3105 | 56.00 | 18.00 | 15.00 | 786.00 | 687.00 | 108.00 | 1.10 | 0.25 | 0.27 | 12.21 | 10.26 | 1.75 | 0.00 | 4.01 |
| Am-bHLH4 | evm.TU.Chr12.2056 | 384.00 | 284.00 | 10211.00 | 73.00 | 56.00 | 41.00 | 5.55 | 2.95 | 138.06 | 0.83 | 0.61 | 0.48 | 0.00 | (6.05) |
| Am-bHLH5 | evm.TU.Chr10.4395 | 653.00 | 350.00 | 4465.00 | 94.00 | 67.00 | 9.00 | 9.77 | 3.76 | 62.37 | 1.11 | 0.76 | 0.10 | 0.00 | (5.15) |
| Am-AP2-1 | evm.TU.Chr5.374 | 128.00 | 92.00 | 10.00 | 2975.00 | 3778.00 | 322.00 | 2.86 | 1.48 | 0.19 | 52.72 | 64.29 | 5.95 | 0.00 | 4.77 |
| Am-AP2-2 | evm.TU.Chr6.87 | 3.00 | 3.00 | 0.00 | 217.00 | 346.00 | 26.00 | 0.12 | 0.12 | 0.00 | 10.59 | 16.25 | 1.28 | 0.00 | 6.44 |
| Am-AP2-3 | evm.TU.Chr2.2554 | 9954.00 | 22849.00 | 11287.00 | 4356.00 | 3857.00 | 4217.00 | 274.81 | 453.44 | 290.88 | 95.25 | 80.97 | 96.12 | 0.01 | (1.89) |
| Am-AP2-4 | evm.TU.Chr7.451 | 398.00 | 315.00 | 351.00 | 2229.00 | 1874.00 | 785.00 | 8.20 | 4.67 | 6.75 | 36.41 | 29.38 | 13.36 | 0.00 | 2.09 |
| Am-AP2-5 | evm.TU.Chr6.2147 | 6505.00 | 5794.00 | 6498.00 | 3462.00 | 3051.00 | 2814.00 | 83.08 | 53.18 | 77.46 | 35.02 | 29.64 | 29.67 | 0.00 | (1.06) |
| Am-bZIP-1 | evm.TU.Chr7.1121 | 25.00 | 47.00 | 5.00 | 3605.00 | 3604.00 | 126.00 | 0.34 | 0.46 | 0.06 | 39.71 | 38.11 | 1.44 | 0.00 | 6.35 |
| Am-bZIP-2 | evm.TU.Chr6.2122 | 1982.00 | 1696.00 | 1160.00 | 305.00 | 313.00 | 114.00 | 22.94 | 14.11 | 12.53 | 2.79 | 2.76 | 1.08 | 0.00 | (2.84) |
| Am-TCP-1 | evm.TU.Chr12.2154 | 167.00 | 151.00 | 91.00 | 10.00 | 6.00 | 25.00 | 2.85 | 1.86 | 1.45 | 0.13 | 0.08 | 0.34 | 0.00 | (3.26) |
| Am-TCP-2 | evm.TU.Chr3.2226 | 932.00 | 769.00 | 909.00 | 202.00 | 316.00 | 147.00 | 16.71 | 9.91 | 15.21 | 2.87 | 4.30 | 2.17 | 0.00 | (2.05) |
| Am-CBF/NF-Y-1 | evm.TU.Chr6.2200 | 19.00 | 54.00 | 8.00 | 0.00 | 0.00 | 0.00 | 1.38 | 2.81 | 0.52 | 0.00 | 0.00 | 0.00 | 0.01 | (7.29) |
| Am-CBF/NF-Y-2 | evm.TU.Chr6.996 | 16349.00 | 14124.00 | 23047.00 | 10174.00 | 7960.00 | 5144.00 | 257.68 | 160.02 | 339.08 | 127.02 | 95.42 | 66.93 | 0.01 | (1.28) |
| Am-CBF/NF-Y-3 | evm.TU.Chr5.984 | 1999.00 | 698.00 | 2156.00 | 62.00 | 25.00 | 185.00 | 83.92 | 21.06 | 84.48 | 2.04 | 0.79 | 6.38 | 0.00 | (4.03) |
| Am-C2H2-1 | evm.TU.Chr10.2642 | 10.00 | 12.00 | 6.00 | 41.00 | 90.00 | 63.00 | 0.25 | 0.23 | 0.14 | 0.88 | 1.87 | 1.41 | 0.01 | 2.75 |
| Am-C2H2-2 | evm.TU.Chr12.2663 | 6791.00 | 1875.00 | 6776.00 | 260.00 | 243.00 | 144.00 | 112.05 | 22.23 | 104.38 | 3.40 | 3.04 | 1.96 | 0.00 | (4.64) |
| Am-C2H2-3 | evm.TU.Chr7.4602 | 5.00 | 15.00 | 0.00 | 380.00 | 1034.00 | 48.00 | 0.15 | 0.33 | 0.00 | 9.31 | 24.33 | 1.20 | 0.00 | 5.98 |
| Am-C2H2-4 | evm.TU.Chr8.109 | 134.00 | 103.00 | 88.00 | 15.00 | 11.00 | 13.00 | 2.90 | 1.60 | 1.77 | 0.25 | 0.17 | 0.23 | 0.00 | (3.11) |
| Am-C2H2-5 | evm.TU.Chr5.745 | 3247.00 | 2501.00 | 4328.00 | 1045.00 | 808.00 | 594.00 | 34.80 | 19.27 | 43.30 | 8.87 | 6.59 | 5.25 | 0.00 | (2.11) |
| Am-PB1 | evm.TU.Chr11.1331 | 24262.00 | 25503.00 | 25170.00 | 13853.00 | 11376.00 | 6824.00 | 205.56 | 155.32 | 199.07 | 92.97 | 73.30 | 47.73 | 0.00 | (1.32) |

| ID | metabolites | mz | rt | KEGG | 25h-1 | 25h-2 | 25h-3 | 25s-1 | 25s-2 | 25s-3 | 25t-1 | 25t-2 | 25t-3 |
| --- | --- | --- | --- | --- | --- | --- | --- | --- | --- | --- | --- | --- | --- |
| F1 | 4-Hydroxycinnamic acid | 146.98 | 785.72 | C00811 | 0.93 | 0.88 | 0.83 | 0.85 | 0.74 | 0.85 | 0.78 | 0.80 | 0.80 |
| F2 | 2,3,4,4,6-Peptahydroxychalcone 4-O-glucoside | 451.12 | 365.38 | C16408 | 0.69 | 0.61 | 0.52 | 0.27 | 0.27 | 0.20 | 0.17 | 0.17 | 0.17 |
| F3 | Sinapoyl aldehyde | 209.08 | 555.86 | C05610 | 1.10 | 1.08 | 1.16 | 0.05 | 0.55 | 0.56 | 0.72 | 0.37 | 0.67 |
| P1 | Naringenin | 273.08 | 654.31 | C00509 | 0.52 | 0.58 | 0.51 | 0.87 | 0.95 | 1.09 | 0.78 | 0.83 | 0.80 |
| P2 | Eriodictyol | 289.07 | 583.10 | C05631 | 0.66 | 0.55 | 0.45 | 0.94 | 0.96 | 1.02 | 0.52 | 0.54 | 0.57 |
| P3 | Cyanidin | 287.06 | 509.57 | C05905 | 0.49 | 0.44 | 0.51 | 1.13 | 1.17 | 1.20 | 0.58 | 0.60 | 0.65 |
| P4 | Epicatechin | 291.09 | 451.95 | C09727 | 0.83 | 0.88 | 0.90 | 0.62 | 0.66 | 0.70 | 0.46 | 0.34 | 0.41 |
| P5 | (-)-Epigallocatechin | 307.08 | 399.50 | C12136 | 0.71 | 1.04 | 1.30 | 0.79 | 0.69 | 0.51 | 0.22 | 0.25 | 0.27 |
| P6 | Isovitexin 2-O-beta-D-glucoside | 595.15 | 367.80 | C04199 | 0.67 | 0.65 | 0.62 | 0.32 | 0.41 | 0.25 | 0.45 | 0.43 | 0.47 |

| gene_id | meta_id | cor |
| --- | --- | --- |
| Am-C2H2-2 | F1 | 0.80 |
| Am-MYB-1 | F2 | -0.88 |
| Am-MYB-5 | F2 | -0.81 |
| Am-MYB-9 | F2 | 0.88 |
| Am-AP2-3 | F2 | 0.90 |
| Am-bHLH4 | F3 | -0.87 |
| Am-bHLH5 | F3 | -0.87 |
| Am-WRKY-5 | P1 | 0.80 |
| Am-TCP-2 | P1 | 0.82 |
| Am-MYB-4 | P1 | 0.83 |
| Am-MYB-3 | P1 | 0.84 |
| Am-MYB-6 | P1 | 0.88 |
| Am-C2H2-4 | P1 | 0.92 |
| Am-TCP-1 | P1 | 0.94 |
| Am-bZIP-2 | P1 | 0.96 |
| Am-bHLH1 | P1 | 0.96 |
| Am-C2H2-1 | P2 | -0.90 |
| Am-MYB-7 | P2 | -0.87 |
| Am-WRKY-1 | P2 | -0.84 |
| Am-bHLH2 | P2 | -0.83 |
| Am-MYB-1 | P2 | -0.81 |
| Am-MYB-9 | P2 | 0.80 |
| Am-CBF/NF-Y-2 | P2 | 0.81 |
| Am-C2H2-2 | P2 | 0.81 |
| Am-WRKY-5 | P2 | 0.81 |
| Am-CBF/NF-Y-3 | P2 | 0.82 |
| Am-C2H2-5 | P2 | 0.86 |
| Am-AP2-3 | P2 | 0.89 |
| Am-bHLH1 | P2 | 0.90 |
| Am-AP2-5 | P2 | 0.92 |
| Am-TCP-2 | P2 | 0.94 |
| Am-TCP-1 | P2 | 0.95 |
| Am-C2H2-4 | P2 | 0.95 |
| Am-bZIP-2 | P2 | 0.95 |
| Am-PB1 | P2 | 0.96 |
| Am-MYB-3 | P2 | 0.97 |
| Am-C2H2-1 | P3 | -0.91 |
| Am-MYB-7 | P3 | -0.87 |
| Am-WRKY-1 | P3 | -0.84 |
| Am-MYB-1 | P3 | -0.83 |
| Am-bHLH2 | P3 | -0.83 |
| Am-CBF/NF-Y-2 | P3 | 0.81 |
| Am-CBF/NF-Y-3 | P3 | 0.81 |
| Am-MYB-9 | P3 | 0.82 |
| Am-C2H2-5 | P3 | 0.86 |
| Am-bHLH1 | P3 | 0.88 |
| Am-AP2-3 | P3 | 0.91 |
| Am-AP2-5 | P3 | 0.91 |
| Am-TCP-2 | P3 | 0.93 |
| Am-TCP-1 | P3 | 0.93 |
| Am-C2H2-4 | P3 | 0.93 |
| Am-bZIP-2 | P3 | 0.93 |
| Am-PB1 | P3 | 0.95 |
| Am-MYB-3 | P3 | 0.96 |
| Am-WRKY-1 | P4 | -0.93 |
| Am-C2H2-1 | P4 | -0.91 |
| Am-MYB-7 | P4 | -0.90 |
| Am-MYB-1 | P4 | -0.90 |
| Am-WRKY-7 | P4 | -0.89 |
| Am-WRKY-4 | P4 | -0.87 |
| Am-WRKY-9 | P4 | -0.87 |
| Am-AP2-2 | P4 | -0.86 |
| Am-AP2-4 | P4 | -0.85 |
| Am-AP2-1 | P4 | -0.85 |
| Am-MYB-8 | P4 | -0.85 |
| Am-WRKY-6 | P4 | -0.83 |
| Am-WRKY-8 | P4 | -0.83 |
| Am-bHLH3 | P4 | -0.82 |
| Am-bZIP-1 | P4 | -0.82 |
| Am-WRKY-3 | P4 | -0.81 |
| Am-C2H2-3 | P4 | -0.81 |
| Am-AP2-3 | P4 | 0.86 |
| Am-TCP-2 | P4 | 0.87 |
| Am-PB1 | P4 | 0.88 |
| Am-AP2-5 | P4 | 0.88 |
| Am-bHLH1 | P4 | 0.90 |
| Am-bZIP-2 | P4 | 0.92 |
| Am-C2H2-4 | P4 | 0.93 |
| Am-TCP-1 | P4 | 0.95 |
| Am-MYB-3 | P4 | 0.97 |
| Am-C2H2-1 | P5 | -0.86 |
| Am-MYB-1 | P5 | -0.85 |
| Am-MYB-7 | P5 | -0.81 |
| Am-TCP-2 | P5 | 0.82 |
| Am-MYB-3 | P5 | 0.83 |
| Am-CBF/NF-Y-2 | P5 | 0.83 |
| Am-C2H2-5 | P5 | 0.85 |
| Am-PB1 | P5 | 0.86 |
| Am-AP2-3 | P5 | 0.88 |
| Am-MYB-9 | P5 | 0.95 |
| Am-WRKY-5 | P6 | -0.99 |
| Am-C2H2-2 | P6 | -0.97 |
| Am-MYB-4 | P6 | -0.96 |
| Am-CBF/NF-Y-3 | P6 | -0.96 |
| Am-TCP-2 | P6 | -0.95 |
| Am-AP2-5 | P6 | -0.95 |
| Am-C2H2-4 | P6 | -0.93 |
| Am-bHLH1 | P6 | -0.91 |
| Am-MYB-3 | P6 | -0.90 |
| Am-bZIP-2 | P6 | -0.90 |
| Am-C2H2-5 | P6 | -0.89 |
| Am-PB1 | P6 | -0.88 |
| Am-TCP-1 | P6 | -0.87 |
| Am-MYB-6 | P6 | -0.86 |
| Am-CBF/NF-Y-2 | P6 | -0.84 |
