# Supplementary material for: Comparative physiological, biochemical, metabolomic, and transcriptomic analyses reveal the formation mechanism of heartwood for Acacia melanoxylon
Source: BMC Plant Biol. 2024 Apr 22;24:308. doi: 10.1186/s12870-024-04884-1 (PMC11034122; doi:10.1186/s12870-024-04884-1)
Supplement: Supplementary file 15 — Additional file 15: Table S9. Differential expressed genes of terpenoids and sucrose metabolism identified in SR25SW vs. SR25TZ. [file 12870_2024_4884_MOESM15_ESM.docx]

**Additional file 15: Table S9.** Differential expressed genes of terpenoids and sucrose metabolism identified in SR25SW vs. SR25TZ.

| #ID | Annotation | FC | log2FC | FDR | regulated | SR25s-3_Count | SR25s-2_Count | SR25s-1_Count | SR25t-3_Count | SR25t-2_Count | SR25t-1_Count | SR25s-3_FPKM | SR25s-2_FPKM | SR25s-1_FPKM | SR25t-3_FPKM | SR25t-2_FPKM | SR25t-1_FPKM |
| --- | --- | --- | --- | --- | --- | --- | --- | --- | --- | --- | --- | --- | --- | --- | --- | --- | --- |
| **ethylene：** |  |  |  |  |  |  |  |  |  |  |  |  |  |  |  |  |  |
| evm.TU.Chr2.501 | 1-aminocyclopropane-1-carboxylate oxidase（ACO） | 116.13 | 6.86 | 0.00 | up | 4.00 | 25.00 | 0.00 | 1475.00 | 2532.00 | 7.00 | 0.08 | 0.47 | 0.00 | 30.86 | 50.89 | 0.14 |
| evm.TU.Chr7.4690 | 1-aminocyclopropane-1-carboxylate oxidase（ACO） | 65.67 | 6.04 | 0.00 | up | 123.00 | 669.00 | 11.00 | 22421.00 | 39833.00 | 443.00 | 3.05 | 11.92 | 0.24 | 440.80 | 751.93 | 9.06 |
| evm.TU.Chr2.496 | 1-aminocyclopropane-1-carboxylate oxidase（ACO） | 14.56 | 3.86 | 0.00 | up | 68.00 | 59.00 | 63.00 | 1077.00 | 1916.00 | 109.00 | 1.28 | 0.80 | 1.11 | 16.13 | 27.57 | 1.69 |
| evm.TU.Chr3.96 | 1-aminocyclopropane-1-carboxylate oxidase（ACO） | 44.13 | 5.46 | 0.00 | up | 21.00 | 345.00 | 152.00 | 9106.00 | 17418.00 | 249.00 | 0.36 | 4.35 | 2.49 | 126.54 | 232.43 | 3.59 |
| **auxin：** |  |  |  |  |  |  |  |  |  |  |  |  |  |  |  |  |  |
| evm.TU.Chr9.1840 | auxin transport | 701.57 | (9.45) | 0.01 | down | 121.00 | 0.00 | 264.00 | 0.00 | 0.00 | 0.00 | 2.36 | 0.00 | 4.81 | 0.00 | 0.00 | 0.00 |
| evm.TU.Chr9.1565 | auxin transport | 491.34 | (8.94) | 0.00 | down | 145.00 | 3.00 | 122.00 | 0.00 | 0.00 | 0.00 | 2.30 | 0.02 | 1.80 | 0.00 | 0.00 | 0.00 |
| Acacia_melanoxylon_newGene_12822 | auxin response | 4324971.00 | (22.04) | 0.00 | down | 137.00 | 0.00 | 0.00 | 0.00 | 0.00 | 0.00 | 0.94 | 0.00 | 0.00 | 0.00 | 0.00 | 0.00 |
| evm.TU.Chr4.2686 | auxin response | 7.81 | (2.97) | 0.00 | down | 6655.00 | 2904.00 | 11851.00 | 965.00 | 1731.00 | 272.00 | 136.66 | 42.86 | 227.20 | 15.69 | 27.02 | 4.60 |
| evm.TU.Chr9.1840 | auxin response | 701.57 | (9.45) | 0.01 | down | 121.00 | 0.00 | 264.00 | 0.00 | 0.00 | 0.00 | 2.36 | 0.00 | 4.81 | 0.00 | 0.00 | 0.00 |
| evm.TU.Chr7.1780 | auxin response | 37.81 | (5.24) | 0.00 | down | 562.00 | 258.00 | 888.00 | 38.00 | 9.00 | 3.00 | 13.96 | 4.60 | 20.59 | 0.74 | 0.17 | 0.06 |
| **GA,gibberellin：** |  |  |  |  |  |  |  |  |  |  |  |  |  |  |  |  |  |
| evm.TU.Chr10.2177 |  | 3.33 | 1.74 | 0.00 | up | 1786.00 | 1959.00 | 2569.00 | 8726.00 | 9675.00 | 4172.00 | 35.49 | 27.99 | 47.66 | 137.37 | 146.25 | 68.45 |
| evm.TU.Chr4.3351 |  | 58.63 | 5.87 | 0.00 | up | 229.00 | 52.00 | 50.00 | 365.00 | 302.00 | 15677.00 | 6.74 | 1.10 | 1.37 | 8.52 | 6.77 | 382.10 |
| Acacia_melanoxylon_newGene_5661 |  | 13.34 | 3.74 | 0.01 | up | 2.00 | 47.00 | 13.00 | 247.00 | 438.00 | 219.00 | 0.10 | 1.79 | 0.64 | 10.47 | 17.89 | 9.69 |
| **SA,Salicylic acid：** |  |  |  |  |  |  |  |  |  |  |  |  |  |  |  |  |  |
| evm.TU.Chr13.3076 |  | 48.41 | 5.60 | 0.00 | up | 4.00 | 6.00 | 0.00 | 76.00 | 435.00 | 33.00 | 0.07 | 0.08 | 0.00 | 1.14 | 6.30 | 0.52 |
| evm.TU.Chr7.3523 |  | 15.73 | 3.98 | 0.01 | up | 28.00 | 99.00 | 12.00 | 1195.00 | 1299.00 | 71.00 | 0.61 | 1.56 | 0.24 | 20.92 | 21.82 | 1.28 |
| **JA,Jasmonic acid：** |  |  |  |  |  |  |  |  |  |  |  |  |  |  |  |  |  |
| evm.TU.Chr4.3232 |  | 69.57 | 6.12 | 0.00 | up | 2.00 | 8.00 | 0.00 | 336.00 | 447.00 | 28.00 | 0.02 | 0.10 | 0.00 | 4.79 | 6.12 | 0.41 |
| Acacia_melanoxylon_newGene_10347 |  | 16.63 | 4.06 | 0.00 | up | 8.00 | 6.00 | 4.00 | 97.00 | 219.00 | 17.00 | 0.27 | 0.13 | 0.11 | 2.78 | 6.04 | 0.49 |
| **CTK，cytokinin：** |  |  |  |  |  |  |  |  |  |  |  |  |  |  |  |  |  |
| evm.TU.Chr2.2673 | cytokinin biosynthetic process | 23.21 | (4.54) | 0.01 | down | 152.00 | 22.00 | 81.00 | 7.00 | 5.00 | 0.00 | 6.13 | 0.62 | 3.06 | 0.21 | 0.14 | 0.00 |
| evm.TU.Chr3.2979 | cytokinin receptor | 11.68 | (3.55) | 0.00 | down | 11439.00 | 7968.00 | 1740.00 | 693.00 | 1035.00 | 253.00 | 79.88 | 39.99 | 11.34 | 3.83 | 5.49 | 1.46 |
| evm.TU.Chr2.2127 | cytokinin biosynthetic process | 4.67 | (2.22) | 0.00 | down | 322.00 | 736.00 | 363.00 | 108.00 | 89.00 | 118.00 | 5.02 | 8.24 | 5.28 | 1.32 | 1.05 | 1.52 |
| evm.TU.Chr7.962 | response to cytokinin | 3.05 | (1.61) | 0.00 | down | 871.00 | 897.00 | 791.00 | 277.00 | 235.00 | 337.00 | 22.43 | 16.61 | 19.02 | 5.65 | 4.60 | 7.16 |
| **CKAP,cytoskeleton-associated protein：** |  |  |  |  |  |  |  |  |  |  |  |  |  |  |  |  |  |
| evm.TU.Chr13.1187 |  | 8.21 | 3.04 | 0.00 | up | 32.00 | 10.00 | 10.00 | 151.00 | 169.00 | 120.00 | 1.73 | 0.39 | 0.52 | 6.65 | 7.15 | 5.51 |
| **Desiccation：** |  |  |  |  |  |  |  |  |  |  |  |  |  |  |  |  |  |
| Acacia_melanoxylon_newGene_9644 | desiccation related | 100.14 | 6.65 | 0.00 | up | 146.00 | 762.00 | 62.00 | 44924.00 | 69017.00 | 1150.00 | 3.35 | 12.61 | 1.32 | 819.93 | 1209.50 | 21.86 |
| **Aquaporin：** |  |  |  |  |  |  |  |  |  |  |  |  |  |  |  |  |  |
| evm.TU.Chr4.2866 | aquaporin-like | 21.46 | (4.42) | 0.00 | down | 548.00 | 498.00 | 3889.00 | 43.00 | 50.00 | 123.00 | 16.11 | 10.52 | 106.85 | 0.99 | 1.10 | 2.99 |
| evm.TU.Chr9.2739 | aquaporin-like | 394.63 | (8.62) | 0.00 | down | 1676.00 | 17.00 | 109.00 | 5.00 | 0.00 | 0.00 | 39.00 | 0.28 | 2.35 | 0.09 | 0.00 | 0.00 |
