# Supplementary material for: Comparative physiological, biochemical, metabolomic, and transcriptomic analyses reveal the formation mechanism of heartwood for Acacia melanoxylon
Source: BMC Plant Biol. 2024 Apr 22;24:308. doi: 10.1186/s12870-024-04884-1 (PMC11034122; doi:10.1186/s12870-024-04884-1)
Supplement: Supplementary file 16 — Additional file 16: Table S10. Gene differential expression of qRT-PCR in horizontal and vertical directions of the trunk. [file 12870_2024_4884_MOESM16_ESM.docx]

**Additional file 16: Table S10.** Gene differential expression of qRT-PCR in horizontal and vertical directions of the trunk.

| gene family | category | 0S1 | 0S2 | 0S3 | 0T1 | 0T2 | 0T3 | 1/4S1 | 1/4S2 | 1/4S3 | 1/4T1 | 1/4T2 | 1/4T3 | 1/2S1 | 1/2S2 | 1/2S3 | 1/2T1 | 1/2T2 | 1/2T3 | 3/4S1 | 3/4S2 | 3/4S3 | 3/4T1 | 3/4T2 | 3/4T3 |
| --- | --- | --- | --- | --- | --- | --- | --- | --- | --- | --- | --- | --- | --- | --- | --- | --- | --- | --- | --- | --- | --- | --- | --- | --- | --- |
| RPL4 | CT1 | 22.76 | 26.20 | 23.69 | 23.23 | 24.51 | 23.96 | 24.01 | 24.27 | 25.95 | 26.88 | 23.88 | 23.96 | 23.86 | 23.92 | 24.42 | 23.49 | 23.00 | 25.22 | 24.13 | 24.17 | 25.96 | 23.76 | 23.42 | 23.25 |
|  | CT2 | 22.75 | 26.54 | 23.65 | 23.29 | 24.48 | 23.93 | 23.95 | 24.11 | 25.94 | 26.81 | 23.83 | 24.09 | 23.66 | 23.56 | 24.07 | 23.67 | 22.90 | 25.62 | 24.25 | 24.28 | 25.79 | 23.57 | 23.41 | 23.30 |
|  | CT3 | 22.73 | 26.12 | 23.69 | 23.21 | 24.35 | 23.98 | 24.21 | 24.42 | 26.16 | 26.45 | 23.83 | 23.86 | 23.68 | 23.96 | 23.95 | 23.56 | 22.85 | 25.20 | 24.12 | 24.38 | 25.96 | 23.51 | 23.54 | 23.19 |
|  | CT平均值 | 22.75 | 26.28 | 23.68 | 23.24 | 24.45 | 23.96 | 24.06 | 24.27 | 26.02 | 26.71 | 23.85 | 23.97 | 23.73 | 23.81 | 24.15 | 23.58 | 22.92 | 25.35 | 24.17 | 24.28 | 25.90 | 23.61 | 23.46 | 23.25 |
|  |  |  |  |  |  |  |  |  |  |  |  |  |  |  |  |  |  |  |  |  |  |  |  |  |  |
| TFs-WRKY71 | CT1 | 28.91 | 27.69 | 27.85 | 21.91 | 22.74 | 23.46 | 30.84 | 31.16 | 29.55 | 26.54 | 23.46 | 23.61 | 28.35 | 28.03 | 30.01 | 23.04 | 21.40 | 23.86 | 30.28 | 29.30 | 32.52 | 21.80 | 21.81 | 21.68 |
|  | CT2 | 28.70 | 26.60 | 27.49 | 22.29 | 22.50 | 23.63 | 30.30 | 29.50 | 30.16 | 26.46 | 23.43 | 23.42 | 27.51 | 27.71 | 30.90 | 22.95 | 21.34 | 23.76 | 31.13 | 28.74 | 31.96 | 21.82 | 21.71 | 21.64 |
|  | CT3 | 28.85 | 27.60 | 27.70 | 21.86 | 22.62 | 23.30 | 29.40 | 31.42 | 28.96 | 26.31 | 23.48 | 23.78 | 27.48 | 28.37 | 31.29 | 23.17 | 21.35 | 23.82 | 29.66 | 30.30 | 30.53 | 21.68 | 21.57 | 21.74 |
|  | CT平均值 | 28.82 | 27.30 | 27.68 | 22.02 | 22.62 | 23.46 | 30.18 | 30.69 | 29.56 | 26.44 | 23.46 | 23.60 | 27.78 | 28.04 | 30.73 | 23.05 | 21.36 | 23.81 | 30.35 | 29.45 | 31.67 | 21.77 | 21.70 | 21.68 |
|  |  |  |  |  |  |  |  |  |  |  |  |  |  |  |  |  |  |  |  |  |  |  |  |  |  |
|  | **△CT** | 6.07 | 1.01 | 4.00 | -1.22 | -1.83 | -0.50 | 6.12 | 6.42 | 3.54 | -0.28 | -0.39 | -0.37 | 4.05 | 4.22 | 6.59 | -0.52 | -1.55 | -1.53 | 6.19 | 5.17 | 5.77 | -1.84 | -1.76 | -1.56 |
|  | **△△CT** | 0.00 | -5.06 | -2.07 | -7.30 | -7.90 | -6.57 | 0.05 | 0.35 | -2.53 | -6.35 | -6.46 | -6.44 | -2.03 | -1.85 | 0.51 | -6.59 | -7.63 | -7.61 | 0.11 | -0.90 | -0.31 | -7.92 | -7.83 | -7.64 |
|  | **2^^-△△CT^** | 1.00 | 33.35 | 4.21 | 157.07 | 239.19 | 94.98 | 0.97 | 0.79 | 5.79 | 81.72 | 88.23 | 87.00 | 4.07 | 3.61 | 0.70 | 96.63 | 197.74 | 195.13 | 0.92 | 1.87 | 1.24 | 241.50 | 228.08 | 198.97 |
|  |  |  |  |  |  |  |  |  |  |  |  |  |  |  |  |  |  |  |  |  |  |  |  |  |  |
|  | **△CT** | 6.16 | 1.41 | 4.17 | -1.33 | -1.71 | -0.50 | 6.79 | 6.89 | 3.53 | -0.18 | -0.38 | -0.36 | 4.62 | 4.21 | 5.86 | -0.53 | -1.52 | -1.48 | 6.11 | 5.02 | 6.62 | -1.81 | -1.64 | -1.57 |
|  |  | 5.95 | 0.32 | 3.81 | -0.95 | -1.95 | -0.33 | 6.24 | 5.23 | 4.15 | -0.26 | -0.42 | -0.55 | 3.78 | 3.90 | 6.75 | -0.63 | -1.58 | -1.59 | 6.96 | 4.47 | 6.06 | -1.79 | -1.75 | -1.61 |
|  |  | 6.10 | 1.31 | 4.02 | -1.38 | -1.83 | -0.65 | 5.34 | 7.15 | 2.94 | -0.40 | -0.37 | -0.19 | 3.75 | 4.56 | 7.15 | -0.40 | -1.56 | -1.53 | 5.49 | 6.03 | 4.63 | -1.93 | -1.88 | -1.51 |
|  |  |  |  |  |  |  |  |  |  |  |  |  |  |  |  |  |  |  |  |  |  |  |  |  |  |
|  | **△△CT** | 0.09 | -4.66 | -1.91 | -7.41 | -7.78 | -6.58 | 0.71 | 0.81 | -2.54 | -6.25 | -6.46 | -6.44 | -1.46 | -1.86 | -0.21 | -6.61 | -7.59 | -7.56 | 0.04 | -1.05 | 0.55 | -7.88 | -7.72 | -7.64 |
|  |  | -0.12 | -5.75 | -2.27 | -7.02 | -8.02 | -6.40 | 0.17 | -0.85 | -1.93 | -6.33 | -6.49 | -6.63 | -2.30 | -2.18 | 0.68 | -6.70 | -7.65 | -7.67 | 0.89 | -1.61 | -0.01 | -7.86 | -7.82 | -7.68 |
|  |  | 0.03 | -4.76 | -2.05 | -7.45 | -7.90 | -6.73 | -0.73 | 1.07 | -3.13 | -6.47 | -6.44 | -6.27 | -2.32 | -1.52 | 1.08 | -6.47 | -7.64 | -7.60 | -0.58 | -0.05 | -1.45 | -8.00 | -7.96 | -7.58 |
|  |  |  |  |  |  |  |  |  |  |  |  |  |  |  |  |  |  |  |  |  |  |  |  |  |  |
|  | **2^^-△△CT^** | 0.94 | 25.36 | 3.75 | 169.93 | 220.19 | 95.46 | 0.61 | 0.57 | 5.82 | 76.26 | 87.80 | 86.66 | 2.75 | 3.63 | 1.16 | 97.39 | 193.29 | 188.39 | 0.98 | 2.08 | 0.69 | 236.15 | 210.66 | 199.82 |
|  |  | 1.09 | 53.98 | 4.81 | 130.01 | 260.14 | 84.71 | 0.89 | 1.80 | 3.80 | 80.47 | 90.07 | 98.72 | 4.91 | 4.52 | 0.62 | 104.17 | 200.80 | 203.08 | 0.54 | 3.05 | 1.01 | 232.64 | 226.69 | 205.50 |
|  |  | 0.98 | 27.10 | 4.14 | 175.40 | 238.91 | 105.97 | 1.66 | 0.47 | 8.76 | 88.91 | 86.85 | 76.96 | 5.00 | 2.86 | 0.47 | 88.94 | 199.20 | 194.20 | 1.50 | 1.03 | 2.73 | 256.39 | 248.45 | 191.82 |
|  | **STD** | 0.08 | 16.05 | 0.54 | 24.78 | 19.99 | 10.63 | 0.54 | 0.74 | 2.50 | 6.44 | 1.65 | 10.90 | 1.28 | 0.83 | 0.36 | 7.63 | 3.96 | 7.40 | 0.48 | 1.01 | 1.10 | 12.82 | 18.97 | 6.87 |
|  |  | 0S1 | 0S2 | 0S3 | 0T1 | 0T2 | 0T3 | 1/4S1 | 1/4S2 | 1/4S3 | 1/4T1 | 1/4T2 | 1/4T3 | 1/2S1 | 1/2S2 | 1/2S3 | 1/2T1 | 1/2T2 | 1/2T3 | 3/4S1 | 3/4S2 | 3/4S3 | 3/4T1 | 3/4T2 | 3/4T3 |
| RPL4 | CT1 | 22.76 | 26.20 | 23.69 | 23.23 | 24.51 | 23.96 | 24.01 | 24.27 | 25.95 | 26.88 | 23.88 | 23.96 | 23.86 | 23.92 | 24.42 | 23.49 | 23.00 | 25.22 | 24.13 | 24.17 | 25.96 | 23.76 | 23.42 | 23.25 |
|  | CT2 | 22.75 | 26.54 | 23.65 | 23.29 | 24.48 | 23.93 | 23.95 | 24.11 | 25.94 | 26.81 | 23.83 | 24.09 | 23.66 | 23.56 | 24.07 | 23.67 | 22.90 | 25.62 | 24.25 | 24.28 | 25.79 | 23.57 | 23.41 | 23.30 |
|  | CT3 | 22.73 | 26.12 | 23.69 | 23.21 | 24.35 | 23.98 | 24.21 | 24.42 | 26.16 | 26.45 | 23.83 | 23.86 | 23.68 | 23.96 | 23.95 | 23.56 | 22.85 | 25.20 | 24.12 | 24.38 | 25.96 | 23.51 | 23.54 | 23.19 |
|  | CT平均值 | 22.75 | 26.28 | 23.68 | 23.24 | 24.45 | 23.96 | 24.06 | 24.27 | 26.02 | 26.71 | 23.85 | 23.97 | 23.73 | 23.81 | 24.15 | 23.58 | 22.92 | 25.35 | 24.17 | 24.28 | 25.90 | 23.61 | 23.46 | 23.25 |
|  |  |  |  |  |  |  |  |  |  |  |  |  |  |  |  |  |  |  |  |  |  |  |  |  |  |
| TFs-WRKY47 | CT1 | 29.76 | 28.84 | 29.89 | 23.45 | 24.21 | 25.34 | 29.99 | 29.97 | 28.98 | 24.49 | 23.46 | 22.81 | 29.53 | 29.65 | 29.44 | 23.75 | 22.56 | 22.57 | 29.72 | 29.11 | 29.53 | 22.42 | 21.94 | 22.04 |
|  | CT2 | 29.11 | 28.48 | 31.29 | 23.50 | 24.60 | 24.94 | 30.43 | 30.38 | 29.31 | 24.79 | 23.38 | 22.93 | 29.10 | 29.37 | 30.11 | 23.72 | 22.88 | 22.69 | 29.78 | 29.80 | 30.06 | 22.47 | 21.99 | 22.00 |
|  | CT3 | 29.84 | 28.00 | 30.08 | 23.53 | 24.33 | 25.27 | 29.20 | 30.11 | 28.54 | 24.75 | 23.34 | 22.88 | 28.96 | 29.71 | 30.14 | 23.54 | 22.69 | 22.47 | 28.83 | 30.26 | 29.81 | 22.44 | 21.78 | 22.13 |
|  | CT平均值 | 29.57 | 28.44 | 30.42 | 23.50 | 24.38 | 25.19 | 29.88 | 30.15 | 28.95 | 24.67 | 23.40 | 22.88 | 29.20 | 29.58 | 29.90 | 23.67 | 22.71 | 22.58 | 29.44 | 29.72 | 29.80 | 22.44 | 21.90 | 22.06 |
|  |  |  |  |  |  |  |  |  |  |  |  |  |  |  |  |  |  |  |  |  |  |  |  |  |  |
|  | **△CT** | 6.82 | 2.16 | 6.74 | 0.25 | -0.07 | 1.23 | 5.82 | 5.88 | 2.93 | -2.04 | -0.45 | -1.10 | 5.46 | 5.76 | 5.75 | 0.10 | -0.21 | -2.77 | 5.28 | 5.44 | 3.90 | -1.17 | -1.55 | -1.19 |
|  | **△△CT** | 0.00 | -4.67 | -0.08 | -6.57 | -6.89 | -5.59 | -1.01 | -0.94 | -3.89 | -8.86 | -7.27 | -7.92 | -1.36 | -1.06 | -1.07 | -6.73 | -7.03 | -9.60 | -1.55 | -1.38 | -2.93 | -7.99 | -8.38 | -8.01 |
|  | **2^^-△△CT^** | 1.00 | 25.42 | 1.06 | 95.12 | 118.75 | 48.31 | 2.01 | 1.92 | 14.86 | 465.76 | 154.75 | 241.99 | 2.57 | 2.09 | 2.10 | 106.00 | 130.87 | 773.97 | 2.92 | 2.60 | 7.61 | 254.41 | 332.62 | 258.36 |
|  |  |  |  |  |  |  |  |  |  |  |  |  |  |  |  |  |  |  |  |  |  |  |  |  |  |
|  | **△CT** | 7.02 | 2.56 | 6.22 | 0.20 | -0.24 | 1.39 | 5.93 | 5.70 | 2.97 | -2.22 | -0.38 | -1.16 | 5.79 | 5.83 | 5.30 | 0.18 | -0.36 | -2.78 | 5.55 | 4.83 | 3.63 | -1.19 | -1.52 | -1.21 |
|  |  | 6.36 | 2.19 | 7.61 | 0.26 | 0.16 | 0.99 | 6.38 | 6.11 | 3.30 | -1.93 | -0.46 | -1.04 | 5.36 | 5.56 | 5.97 | 0.14 | -0.04 | -2.66 | 5.61 | 5.52 | 4.16 | -1.14 | -1.46 | -1.24 |
|  |  | 7.09 | 1.72 | 6.40 | 0.29 | -0.12 | 1.32 | 5.15 | 5.83 | 2.53 | -1.97 | -0.50 | -1.09 | 5.23 | 5.90 | 6.00 | -0.03 | -0.23 | -2.88 | 4.66 | 5.98 | 3.90 | -1.17 | -1.68 | -1.12 |
|  |  |  |  |  |  |  |  |  |  |  |  |  |  |  |  |  |  |  |  |  |  |  |  |  |  |
|  | **△△CT** | 0.19 | -4.26 | -0.61 | -6.62 | -7.06 | -5.44 | -0.89 | -1.13 | -3.86 | -9.05 | -7.21 | -7.98 | -1.03 | -0.99 | -1.53 | -6.65 | -7.18 | -9.60 | -1.27 | -1.99 | -3.20 | -8.01 | -8.34 | -8.03 |
|  |  | -0.46 | -4.63 | 0.78 | -6.56 | -6.67 | -5.84 | -0.45 | -0.72 | -3.53 | -8.75 | -7.29 | -7.86 | -1.46 | -1.27 | -0.86 | -6.68 | -6.86 | -9.48 | -1.21 | -1.30 | -2.67 | -7.96 | -8.29 | -8.07 |
|  |  | 0.27 | -5.11 | -0.43 | -6.53 | -6.95 | -5.51 | -1.68 | -0.99 | -4.30 | -8.79 | -7.33 | -7.91 | -1.59 | -0.93 | -0.83 | -6.86 | -7.05 | -9.70 | -2.16 | -0.84 | -2.92 | -7.99 | -8.51 | -7.94 |
|  |  |  |  |  |  |  |  |  |  |  |  |  |  |  |  |  |  |  |  |  |  |  |  |  |  |
|  | **2^^-△△CT^** | 0.87 | 19.21 | 1.52 | 98.28 | 133.60 | 43.34 | 1.85 | 2.18 | 14.49 | 528.96 | 147.68 | 252.99 | 2.04 | 1.98 | 2.88 | 100.13 | 144.95 | 777.46 | 2.41 | 3.97 | 9.17 | 258.66 | 324.02 | 261.53 |
|  |  | 1.38 | 24.80 | 0.58 | 94.51 | 101.65 | 57.19 | 1.36 | 1.64 | 11.52 | 431.11 | 156.11 | 232.82 | 2.75 | 2.41 | 1.81 | 102.74 | 116.38 | 716.35 | 2.31 | 2.47 | 6.35 | 249.67 | 312.61 | 267.99 |
|  |  | 0.83 | 34.47 | 1.35 | 92.66 | 123.30 | 45.50 | 3.20 | 1.98 | 19.68 | 443.06 | 160.75 | 240.57 | 3.01 | 1.90 | 1.77 | 115.78 | 132.87 | 832.47 | 4.47 | 1.79 | 7.56 | 254.97 | 363.31 | 246.05 |
|  | **STD** | 0.30 | 7.72 | 0.50 | 2.87 | 16.30 | 7.45 | 0.95 | 0.27 | 4.13 | 53.38 | 6.62 | 10.18 | 0.50 | 0.27 | 0.63 | 8.38 | 14.34 | 58.08 | 1.22 | 1.12 | 1.41 | 4.52 | 26.60 | 11.27 |
|  |  | 0S1 | 0S2 | 0S3 | 0T1 | 0T2 | 0T3 | 1/4S1 | 1/4S2 | 1/4S3 | 1/4T1 | 1/4T2 | 1/4T3 | 1/2S1 | 1/2S2 | 1/2S3 | 1/2T1 | 1/2T2 | 1/2T3 | 3/4S1 | 3/4S2 | 3/4S3 | 3/4T1 | 3/4T2 | 3/4T3 |
| RPL4 | CT1 | 23.23 | 24.51 | 23.96 | 22.76 | 26.20 | 23.69 | 24.02 | 24.22 | 24.48 | 26.88 | 23.88 | 23.96 | 23.86 | 23.92 | 24.42 | 23.49 | 23.00 | 25.22 | 23.76 | 23.42 | 23.25 | 24.13 | 24.17 | 25.96 |
|  | CT2 | 23.29 | 24.48 | 23.93 | 22.75 | 26.54 | 23.65 | 24.06 | 23.98 | 24.22 | 26.81 | 23.83 | 24.09 | 23.66 | 23.56 | 24.07 | 23.67 | 22.90 | 25.62 | 23.57 | 23.41 | 23.30 | 24.25 | 24.28 | 25.79 |
|  | CT3 | 23.21 | 24.35 | 23.98 | 22.73 | 26.12 | 23.69 | 23.98 | 24.00 | 24.19 | 26.45 | 23.83 | 23.86 | 23.68 | 23.96 | 23.95 | 23.56 | 22.85 | 25.20 | 23.51 | 23.54 | 23.19 | 24.12 | 24.38 | 25.96 |
|  | CT平均值 | 23.24 | 24.45 | 23.96 | 22.75 | 26.28 | 23.68 | 24.02 | 24.07 | 24.30 | 26.71 | 23.85 | 23.97 | 23.73 | 23.81 | 24.15 | 23.58 | 22.92 | 25.35 | 23.61 | 23.46 | 23.25 | 24.17 | 24.28 | 25.90 |
|  |  |  |  |  |  |  |  |  |  |  |  |  |  |  |  |  |  |  |  |  |  |  |  |  |  |
| HCT | CT1 | 27.74 | 28.77 | 28.91 | 25.77 | 29.24 | 26.94 | 26.84 | 26.21 | 27.34 | 26.76 | 29.91 | 29.53 | 25.80 | 27.27 | 28.13 | 28.07 | 27.73 | 27.84 | 27.47 | 26.83 | 27.31 | 26.05 | 26.72 | 27.75 |
|  | CT2 | 27.51 | 28.62 | 28.61 | 25.67 | 29.64 | 26.77 | 26.58 | 25.84 | 27.08 | 26.58 | 29.77 | 28.96 | 26.02 | 27.05 | 27.44 | 28.08 | 27.37 | 27.81 | 28.10 | 27.03 | 27.18 | 26.16 | 26.93 | 28.05 |
|  | CT3 | 27.66 | 28.62 | 28.18 | 25.69 | 29.21 | 26.94 | 26.61 | 25.96 | 27.36 | 26.86 | 29.90 | 29.94 | 25.93 | 27.03 | 27.44 | 27.71 | 27.46 | 27.83 | 27.46 | 27.13 | 27.18 | 26.23 | 26.66 | 27.85 |
|  | CT平均值 | 27.64 | 28.67 | 28.57 | 25.71 | 29.36 | 26.89 | 26.68 | 26.00 | 27.26 | 26.74 | 29.86 | 29.48 | 25.92 | 27.12 | 27.67 | 27.95 | 27.52 | 27.82 | 27.68 | 27.00 | 27.22 | 26.15 | 26.77 | 27.88 |
|  |  |  |  |  |  |  |  |  |  |  |  |  |  |  |  |  |  |  |  |  |  |  |  |  |  |
|  | **△CT** | 4.39 | 4.22 | 4.61 | 2.96 | 3.08 | 3.21 | 2.66 | 1.93 | 2.96 | 0.02 | 6.02 | 5.50 | 2.19 | 3.30 | 3.52 | 4.38 | 4.60 | 2.48 | 4.06 | 3.54 | 3.98 | 1.98 | 2.49 | 1.98 |
|  | **△△CT** | 0.00 | -0.17 | 0.21 | -1.43 | -1.31 | -1.19 | -1.73 | -2.46 | -1.43 | -4.37 | 1.62 | 1.11 | -2.21 | -1.09 | -0.87 | -0.02 | 0.21 | -1.92 | -0.33 | -0.85 | -0.42 | -2.41 | -1.90 | -2.41 |
|  | **2^^-△△CT^** | 1.00 | 1.13 | 0.86 | 2.70 | 2.49 | 2.28 | 3.32 | 5.50 | 2.70 | 20.71 | 0.33 | 0.46 | 4.62 | 2.13 | 1.83 | 1.01 | 0.86 | 3.78 | 1.26 | 1.81 | 1.33 | 5.33 | 3.73 | 5.33 |
|  |  |  |  |  |  |  |  |  |  |  |  |  |  |  |  |  |  |  |  |  |  |  |  |  |  |
|  | **△CT** | 4.99 | 2.49 | 5.23 | 2.52 | 4.79 | 2.98 | 2.78 | 1.93 | 1.32 | 0.05 | 6.06 | 5.56 | 2.07 | 3.46 | 3.99 | 4.49 | 4.82 | 2.49 | 3.30 | 2.55 | 1.41 | 2.44 | 3.26 | 4.50 |
|  |  | 4.76 | 2.33 | 4.93 | 2.43 | 5.19 | 2.81 | 2.52 | 1.57 | 1.06 | -0.13 | 5.92 | 4.99 | 2.29 | 3.24 | 3.29 | 4.51 | 4.45 | 2.46 | 3.94 | 2.75 | 1.28 | 2.55 | 3.48 | 4.81 |
|  |  | 4.91 | 2.34 | 4.50 | 2.44 | 4.76 | 2.98 | 2.56 | 1.69 | 1.34 | 0.15 | 6.06 | 5.97 | 2.20 | 3.21 | 3.30 | 4.14 | 4.55 | 2.48 | 3.29 | 2.86 | 1.28 | 2.62 | 3.20 | 4.60 |
|  |  |  |  |  |  |  |  |  |  |  |  |  |  |  |  |  |  |  |  |  |  |  |  |  |  |
|  | **△△CT** | 0.60 | -1.91 | 0.84 | -1.87 | 0.40 | -1.41 | -1.62 | -2.46 | -3.07 | -4.35 | 1.67 | 1.17 | -2.32 | -0.94 | -0.41 | 0.10 | 0.42 | -1.91 | -1.09 | -1.84 | -2.98 | -1.96 | -1.14 | 0.11 |
|  |  | 0.37 | -2.06 | 0.54 | -1.97 | 0.80 | -1.58 | -1.87 | -2.83 | -3.33 | -4.52 | 1.53 | 0.59 | -2.11 | -1.16 | -1.10 | 0.11 | 0.06 | -1.93 | -0.46 | -1.64 | -3.12 | -1.85 | -0.92 | 0.41 |
|  |  | 0.52 | -2.05 | 0.11 | -1.95 | 0.37 | -1.41 | -1.84 | -2.70 | -3.05 | -4.25 | 1.66 | 1.57 | -2.19 | -1.18 | -1.10 | -0.26 | 0.15 | -1.92 | -1.10 | -1.54 | -3.12 | -1.77 | -1.19 | 0.20 |
|  |  |  |  |  |  |  |  |  |  |  |  |  |  |  |  |  |  |  |  |  |  |  |  |  |  |
|  | **2^^-△△CT^** | 0.66 | 3.75 | 0.56 | 3.66 | 0.76 | 2.66 | 3.06 | 5.50 | 8.41 | 20.36 | 0.31 | 0.45 | 5.00 | 1.91 | 1.33 | 0.93 | 0.75 | 3.75 | 2.13 | 3.58 | 7.92 | 3.88 | 2.20 | 0.93 |
|  |  | 0.77 | 4.17 | 0.69 | 3.91 | 0.57 | 2.99 | 3.66 | 7.10 | 10.08 | 23.02 | 0.35 | 0.66 | 4.31 | 2.23 | 2.15 | 0.93 | 0.96 | 3.82 | 1.37 | 3.13 | 8.67 | 3.59 | 1.89 | 0.75 |
|  |  | 0.70 | 4.15 | 0.93 | 3.87 | 0.77 | 2.66 | 3.57 | 6.51 | 8.28 | 18.97 | 0.32 | 0.34 | 4.57 | 2.27 | 2.14 | 1.19 | 0.90 | 3.78 | 2.15 | 2.90 | 8.67 | 3.42 | 2.28 | 0.87 |
|  | **STD** | 0.06 | 0.24 | 0.19 | 0.14 | 0.11 | 0.19 | 0.32 | 0.81 | 1.01 | 2.06 | 0.02 | 0.17 | 0.35 | 0.19 | 0.47 | 0.15 | 0.11 | 0.03 | 0.44 | 0.35 | 0.43 | 0.23 | 0.21 | 0.09 |
|  |  | 0S1 | 0S2 | 0S3 | 0T1 | 0T2 | 0T3 | 1/4S1 | 1/4S2 | 1/4S3 | 1/4T1 | 1/4T2 | 1/4T3 | 1/2S1 | 1/2S2 | 1/2S3 | 1/2T1 | 1/2T2 | 1/2T3 | 3/4S1 | 3/4S2 | 3/4S3 | 3/4T1 | 3/4T2 | 3/4T3 |
| RPL4 | CT1 | 22.76 | 26.20 | 23.69 | 23.23 | 24.51 | 23.96 | 24.01 | 24.27 | 25.95 | 26.88 | 23.88 | 23.96 | 23.86 | 23.92 | 24.42 | 23.49 | 23.00 | 25.22 | 24.13 | 24.17 | 25.96 | 23.76 | 23.42 | 23.25 |
|  | CT2 | 22.75 | 26.54 | 23.65 | 23.29 | 24.48 | 23.93 | 23.95 | 24.11 | 25.94 | 26.81 | 23.83 | 24.09 | 23.66 | 23.56 | 24.07 | 23.67 | 22.90 | 25.62 | 24.25 | 24.28 | 25.79 | 23.57 | 23.41 | 23.30 |
|  | CT3 | 22.73 | 26.12 | 23.69 | 23.21 | 24.35 | 23.98 | 24.21 | 24.42 | 26.16 | 26.45 | 23.83 | 23.86 | 23.68 | 23.96 | 23.95 | 23.56 | 22.85 | 25.20 | 24.12 | 24.38 | 25.96 | 23.51 | 23.54 | 23.19 |
|  | CT平均值 | 22.75 | 26.28 | 23.68 | 23.24 | 24.45 | 23.96 | 24.06 | 24.27 | 26.02 | 26.71 | 23.85 | 23.97 | 23.73 | 23.81 | 24.15 | 23.58 | 22.92 | 25.35 | 24.17 | 24.28 | 25.90 | 23.61 | 23.46 | 23.25 |
|  |  |  |  |  |  |  |  |  |  |  |  |  |  |  |  |  |  |  |  |  |  |  |  |  |  |
| F3'H | CT1 | 30.03 | 23.20 | 28.24 | 17.74 | 18.55 | 19.41 | 28.67 | 29.36 | 28.01 | 19.61 | 17.50 | 17.68 | 26.62 | 29.86 | 29.75 | 19.11 | 16.95 | 17.37 | 27.90 | 30.51 | 29.98 | 16.95 | 15.63 | 16.47 |
|  | CT2 | 30.03 | 23.37 | 28.21 | 17.89 | 18.44 | 19.49 | 28.21 | 29.36 | 28.32 | 19.48 | 17.49 | 17.69 | 26.07 | 29.30 | 29.98 | 18.91 | 16.91 | 17.35 | 28.22 | 31.33 | 29.96 | 16.37 | 15.85 | 16.30 |
|  | CT3 | 30.25 | 23.56 | 28.11 | 17.83 | 18.66 | 19.61 | 28.38 | 29.96 | 28.18 | 19.61 | 17.46 | 17.55 | 26.27 | 29.70 | 29.55 | 18.98 | 16.94 | 17.33 | 27.76 | 30.16 | 30.52 | 17.22 | 16.05 | 16.45 |
|  | CT平均值 | 30.10 | 23.38 | 28.19 | 17.82 | 18.55 | 19.50 | 28.42 | 29.56 | 28.17 | 19.57 | 17.48 | 17.64 | 26.32 | 29.62 | 29.76 | 19.00 | 16.93 | 17.35 | 27.96 | 30.67 | 30.16 | 16.84 | 15.84 | 16.41 |
|  |  |  |  |  |  |  |  |  |  |  |  |  |  |  |  |  |  |  |  |  |  |  |  |  |  |
|  | **△CT** | 7.36 | -2.91 | 4.51 | -5.42 | -5.90 | -4.46 | 4.36 | 5.29 | 2.15 | -7.15 | -6.36 | -6.33 | 2.59 | 5.80 | 5.62 | -4.58 | -5.98 | -8.00 | 3.79 | 6.39 | 4.25 | -6.77 | -7.62 | -6.84 |
|  | **△△CT** | 0.00 | -10.27 | -2.85 | -12.78 | -13.26 | -11.81 | -3.00 | -2.07 | -5.20 | -14.50 | -13.72 | -13.69 | -4.77 | -1.56 | -1.74 | -11.93 | -13.34 | -15.35 | -3.57 | -0.97 | -3.10 | -14.13 | -14.97 | -14.20 |
|  | **2^^-△△CT^** | 1.00 | 1232.69 | 7.21 | 7035.35 | 9784.85 | 3599.94 | 7.98 | 4.19 | 36.85 | 23248.19 | 13512.30 | 13197.06 | 27.27 | 2.94 | 3.35 | 3910.65 | 10371.98 | 41888.94 | 11.84 | 1.95 | 8.60 | 17869.25 | 32164.01 | 18782.09 |
|  |  |  |  |  |  |  |  |  |  |  |  |  |  |  |  |  |  |  |  |  |  |  |  |  |  |
|  | **△CT** | 7.29 | -3.09 | 4.56 | -5.50 | -5.90 | -4.55 | 4.61 | 5.08 | 2.00 | -7.10 | -6.35 | -6.29 | 2.89 | 6.04 | 5.61 | -4.47 | -5.96 | -7.98 | 3.73 | 6.24 | 4.08 | -6.67 | -7.83 | -6.78 |
|  |  | 7.28 | -2.92 | 4.53 | -5.35 | -6.01 | -4.47 | 4.15 | 5.09 | 2.30 | -7.23 | -6.36 | -6.28 | 2.33 | 5.48 | 5.83 | -4.67 | -6.01 | -8.00 | 4.05 | 7.05 | 4.06 | -7.24 | -7.61 | -6.95 |
|  |  | 7.51 | -2.73 | 4.44 | -5.42 | -5.78 | -4.35 | 4.32 | 5.69 | 2.17 | -7.10 | -6.38 | -6.42 | 2.54 | 5.89 | 5.41 | -4.59 | -5.98 | -8.01 | 3.59 | 5.88 | 4.62 | -6.39 | -7.41 | -6.80 |
|  |  |  |  |  |  |  |  |  |  |  |  |  |  |  |  |  |  |  |  |  |  |  |  |  |  |
|  | **△△CT** | -0.07 | -10.44 | -2.80 | -12.86 | -13.26 | -11.91 | -2.74 | -2.27 | -5.36 | -14.46 | -13.71 | -13.65 | -4.47 | -1.32 | -1.75 | -11.83 | -13.32 | -15.34 | -3.62 | -1.12 | -3.28 | -14.02 | -15.19 | -14.13 |
|  |  | -0.08 | -10.28 | -2.83 | -12.71 | -13.37 | -11.83 | -3.21 | -2.26 | -5.06 | -14.59 | -13.72 | -13.64 | -5.02 | -1.88 | -1.53 | -12.02 | -13.36 | -15.36 | -3.31 | -0.31 | -3.30 | -14.60 | -14.97 | -14.30 |
|  |  | 0.15 | -10.08 | -2.92 | -12.77 | -13.14 | -11.70 | -3.04 | -1.67 | -5.19 | -14.46 | -13.74 | -13.78 | -4.82 | -1.47 | -1.95 | -11.95 | -13.34 | -15.37 | -3.77 | -1.47 | -2.74 | -13.75 | -14.77 | -14.15 |
|  |  |  |  |  |  |  |  |  |  |  |  |  |  |  |  |  |  |  |  |  |  |  |  |  |  |
|  | **2^^-△△CT^** | 1.05 | 1393.09 | 6.96 | 7425.96 | 9811.57 | 3851.21 | 6.70 | 4.83 | 41.08 | 22578.36 | 13379.49 | 12842.27 | 22.11 | 2.49 | 3.37 | 3631.64 | 10235.19 | 41348.95 | 12.32 | 2.17 | 9.72 | 16658.39 | 37296.11 | 17984.39 |
|  |  | 1.06 | 1239.71 | 7.10 | 6695.66 | 10565.13 | 3631.90 | 9.22 | 4.81 | 33.33 | 24708.45 | 13478.86 | 12754.02 | 32.52 | 3.67 | 2.88 | 4163.37 | 10535.80 | 41918.14 | 9.90 | 1.24 | 9.82 | 24880.56 | 32013.01 | 20218.63 |
|  |  | 0.90 | 1084.58 | 7.58 | 7003.44 | 9037.52 | 3335.44 | 8.22 | 3.17 | 36.56 | 22523.17 | 13680.29 | 14032.73 | 28.20 | 2.77 | 3.86 | 3955.46 | 10347.18 | 42406.43 | 13.60 | 2.78 | 6.66 | 13766.57 | 27868.95 | 18221.50 |
|  | **STD** | 0.09 | 154.26 | 0.32 | 366.65 | 763.83 | 258.84 | 1.27 | 0.95 | 3.89 | 1246.05 | 153.26 | 714.15 | 5.23 | 0.62 | 0.49 | 267.96 | 151.92 | 529.25 | 1.88 | 0.78 | 1.79 | 5766.10 | 4725.03 | 1227.23 |
|  |  | 0S1 | 0S2 | 0S3 | 0T1 | 0T2 | 0T3 | 1/4S1 | 1/4S2 | 1/4S3 | 1/4T1 | 1/4T2 | 1/4T3 | 1/2S1 | 1/2S2 | 1/2S3 | 1/2T1 | 1/2T2 | 1/2T3 | 3/4S1 | 3/4S2 | 3/4S3 | 3/4T1 | 3/4T2 | 3/4T3 |
| RPL4 | CT1 | 22.76 | 26.20 | 23.69 | 23.23 | 24.51 | 23.96 | 24.01 | 24.27 | 25.95 | 26.88 | 23.88 | 23.96 | 23.86 | 23.92 | 24.42 | 23.49 | 23.00 | 25.22 | 24.13 | 24.17 | 25.96 | 23.76 | 23.42 | 23.25 |
|  | CT2 | 22.75 | 26.54 | 23.65 | 23.29 | 24.48 | 23.93 | 23.95 | 24.11 | 25.94 | 26.81 | 23.83 | 24.09 | 23.66 | 23.56 | 24.07 | 23.67 | 22.90 | 25.62 | 24.25 | 24.28 | 25.79 | 23.57 | 23.41 | 23.30 |
|  | CT3 | 22.73 | 26.12 | 23.69 | 23.21 | 24.35 | 23.98 | 24.21 | 24.42 | 26.16 | 26.45 | 23.83 | 23.86 | 23.68 | 23.96 | 23.95 | 23.56 | 22.85 | 25.20 | 24.12 | 24.38 | 25.96 | 23.51 | 23.54 | 23.19 |
|  | CT平均值 | 22.75 | 26.28 | 23.68 | 23.24 | 24.45 | 23.96 | 24.06 | 24.27 | 26.02 | 26.71 | 23.85 | 23.97 | 23.73 | 23.81 | 24.15 | 23.58 | 22.92 | 25.35 | 24.17 | 24.28 | 25.90 | 23.61 | 23.46 | 23.25 |
|  |  |  |  |  |  |  |  |  |  |  |  |  |  |  |  |  |  |  |  |  |  |  |  |  |  |
| CAD | CT1 | 30.72 | 31.51 | 31.37 | 28.16 | 25.94 | 29.87 | 30.95 | 30.51 | 31.38 | 29.83 | 28.71 | 26.98 | 30.22 | 30.68 | 30.43 | 28.02 | 26.43 | 27.66 | 29.45 | 31.48 | 30.79 | 25.60 | 26.21 | 25.14 |
|  | CT2 | 31.59 | 31.20 | 31.41 | 28.00 | 25.63 | 29.16 | 30.40 | 29.86 | 31.20 | 30.91 | 29.09 | 26.99 | 30.51 | 30.65 | 30.88 | 27.54 | 26.13 | 27.61 | 29.71 | 30.36 | 30.60 | 25.56 | 26.20 | 24.91 |
|  | CT3 | 30.86 | 31.82 | 32.58 | 28.44 | 25.70 | 29.41 | 30.90 | 30.73 | 31.23 | 29.90 | 28.83 | 27.14 | 30.67 | 30.59 | 30.85 | 27.93 | 26.17 | 27.55 | 30.06 | 30.56 | 30.88 | 25.55 | 26.50 | 24.98 |
|  | CT平均值 | 31.06 | 31.51 | 31.79 | 28.20 | 25.76 | 29.48 | 30.75 | 30.36 | 31.27 | 30.21 | 28.88 | 27.04 | 30.47 | 30.64 | 30.72 | 27.83 | 26.24 | 27.61 | 29.74 | 30.80 | 30.76 | 25.57 | 26.30 | 25.01 |
|  |  |  |  |  |  |  |  |  |  |  |  |  |  |  |  |  |  |  |  |  |  |  |  |  |  |
|  | **△CT** | 8.31 | 5.22 | 8.11 | 4.96 | 1.31 | 5.52 | 6.69 | 6.09 | 5.25 | 3.50 | 5.03 | 3.07 | 6.73 | 6.82 | 6.57 | 4.26 | 3.33 | 2.26 | 5.57 | 6.53 | 4.85 | 1.96 | 2.85 | 1.76 |
|  | **△△CT** | 0.00 | -3.09 | -0.20 | -3.36 | -7.00 | -2.79 | -1.62 | -2.22 | -3.06 | -4.82 | -3.28 | -5.25 | -1.58 | -1.49 | -1.74 | -4.05 | -4.99 | -6.05 | -2.74 | -1.78 | -3.46 | -6.36 | -5.47 | -6.55 |
|  | **2^^-△△CT^** | 1.00 | 8.51 | 1.15 | 10.23 | 128.14 | 6.90 | 3.07 | 4.65 | 8.33 | 28.17 | 9.72 | 37.96 | 2.98 | 2.81 | 3.34 | 16.62 | 31.68 | 66.40 | 6.67 | 3.45 | 11.00 | 81.94 | 44.20 | 93.63 |
|  |  |  |  |  |  |  |  |  |  |  |  |  |  |  |  |  |  |  |  |  |  |  |  |  |  |
|  | **△CT** | 7.98 | 5.22 | 7.69 | 4.91 | 1.49 | 5.92 | 6.89 | 6.24 | 5.36 | 3.11 | 4.86 | 3.01 | 6.49 | 6.86 | 6.28 | 4.44 | 3.51 | 2.31 | 5.29 | 7.21 | 4.88 | 1.99 | 2.75 | 1.89 |
|  |  | 8.84 | 4.91 | 7.73 | 4.76 | 1.18 | 5.20 | 6.34 | 5.58 | 5.19 | 4.19 | 5.24 | 3.02 | 6.78 | 6.83 | 6.73 | 3.97 | 3.21 | 2.26 | 5.54 | 6.09 | 4.69 | 1.94 | 2.75 | 1.66 |
|  |  | 8.12 | 5.53 | 8.90 | 5.20 | 1.26 | 5.46 | 6.84 | 6.46 | 5.21 | 3.18 | 4.99 | 3.17 | 6.93 | 6.77 | 6.70 | 4.36 | 3.25 | 2.21 | 5.89 | 6.29 | 4.98 | 1.93 | 3.04 | 1.74 |
|  |  |  |  |  |  |  |  |  |  |  |  |  |  |  |  |  |  |  |  |  |  |  |  |  |  |
|  | **△△CT** | -0.33 | -3.09 | -0.62 | -3.40 | -6.82 | -2.40 | -1.42 | -2.08 | -2.95 | -5.20 | -3.45 | -5.30 | -1.82 | -1.45 | -2.03 | -3.87 | -4.80 | -6.00 | -3.02 | -1.11 | -3.43 | -6.32 | -5.56 | -6.42 |
|  |  | 0.53 | -3.40 | -0.58 | -3.55 | -7.13 | -3.11 | -1.97 | -2.73 | -3.12 | -4.12 | -3.07 | -5.29 | -1.53 | -1.48 | -1.58 | -4.34 | -5.10 | -6.05 | -2.77 | -2.22 | -3.62 | -6.37 | -5.57 | -6.65 |
|  |  | -0.19 | -2.78 | 0.59 | -3.11 | -7.05 | -2.86 | -1.47 | -1.85 | -3.10 | -5.13 | -3.33 | -5.14 | -1.38 | -1.54 | -1.61 | -3.95 | -5.06 | -6.11 | -2.42 | -2.02 | -3.33 | -6.38 | -5.27 | -6.57 |
|  |  |  |  |  |  |  |  |  |  |  |  |  |  |  |  |  |  |  |  |  |  |  |  |  |  |
|  | **2^^-△△CT^** | 1.26 | 8.51 | 1.53 | 10.55 | 113.03 | 5.26 | 2.67 | 4.22 | 7.73 | 36.77 | 10.92 | 39.48 | 3.53 | 2.73 | 4.08 | 14.60 | 27.88 | 64.10 | 8.13 | 2.15 | 10.76 | 80.16 | 47.27 | 85.83 |
|  |  | 0.69 | 10.54 | 1.50 | 11.72 | 140.02 | 8.63 | 3.92 | 6.62 | 8.72 | 17.39 | 8.38 | 39.26 | 2.90 | 2.79 | 2.99 | 20.29 | 34.25 | 66.33 | 6.82 | 4.67 | 12.29 | 82.53 | 47.38 | 100.41 |
|  |  | 1.14 | 6.86 | 0.66 | 8.66 | 132.93 | 7.24 | 2.77 | 3.60 | 8.58 | 34.98 | 10.03 | 35.28 | 2.60 | 2.91 | 3.05 | 15.49 | 33.29 | 68.84 | 5.35 | 4.07 | 10.06 | 83.18 | 38.55 | 95.24 |
|  | **STD** | 0.30 | 1.85 | 0.49 | 1.54 | 13.99 | 1.69 | 0.69 | 1.59 | 0.54 | 10.71 | 1.29 | 2.36 | 0.48 | 0.09 | 0.62 | 3.06 | 3.43 | 2.37 | 1.39 | 1.32 | 1.14 | 1.59 | 5.06 | 7.40 |
|  |  | 0S1 | 0S2 | 0S3 | 0T1 | 0T2 | 0T3 | 1/4S1 | 1/4S2 | 1/4S3 | 1/4T1 | 1/4T2 | 1/4T3 | 1/2S1 | 1/2S2 | 1/2S3 | 1/2T1 | 1/2T2 | 1/2T3 | 3/4S1 | 3/4S2 | 3/4S3 | 3/4T1 | 3/4T2 | 3/4T3 |
| RPL4 | CT1 | 22.76 | 26.20 | 23.69 | 23.23 | 24.51 | 23.96 | 24.01 | 24.27 | 25.95 | 26.88 | 23.88 | 23.96 | 23.86 | 23.92 | 24.42 | 23.49 | 23.00 | 25.22 | 24.13 | 24.17 | 25.96 | 23.76 | 23.42 | 23.25 |
|  | CT2 | 22.75 | 26.54 | 23.65 | 23.29 | 24.48 | 23.93 | 23.95 | 24.11 | 25.94 | 26.81 | 23.83 | 24.09 | 23.66 | 23.56 | 24.07 | 23.67 | 22.90 | 25.62 | 24.25 | 24.28 | 25.79 | 23.57 | 23.41 | 23.30 |
|  | CT3 | 22.73 | 26.12 | 23.69 | 23.21 | 24.35 | 23.98 | 24.21 | 24.42 | 26.16 | 26.45 | 23.83 | 23.86 | 23.68 | 23.96 | 23.95 | 23.56 | 22.85 | 25.20 | 24.12 | 24.38 | 25.96 | 23.51 | 23.54 | 23.19 |
|  | CT平均值 | 22.75 | 26.28 | 23.68 | 23.24 | 24.45 | 23.96 | 24.06 | 24.27 | 26.02 | 26.71 | 23.85 | 23.97 | 23.73 | 23.81 | 24.15 | 23.58 | 22.92 | 25.35 | 24.17 | 24.28 | 25.90 | 23.61 | 23.46 | 23.25 |
|  |  |  |  |  |  |  |  |  |  |  |  |  |  |  |  |  |  |  |  |  |  |  |  |  |  |
| PAL | CT1 | 28.99 | 27.12 | 29.55 | 22.18 | 23.13 | 24.05 | 29.72 | 29.54 | 29.99 | 24.79 | 22.71 | 22.28 | 27.91 | 29.97 | 29.25 | 23.80 | 21.91 | 22.87 | 29.17 | 29.69 | 29.48 | 21.65 | 20.75 | 21.74 |
|  | CT2 | 29.61 | 27.43 | 29.70 | 22.17 | 23.02 | 23.80 | 29.54 | 29.35 | 29.89 | 24.58 | 22.61 | 22.65 | 27.57 | 30.75 | 29.35 | 23.98 | 21.90 | 22.94 | 29.43 | 29.91 | 29.42 | 21.70 | 21.01 | 21.70 |
|  | CT3 | 29.81 | 27.51 | 29.34 | 22.43 | 23.44 | 24.22 | 29.97 | 29.68 | 29.75 | 24.57 | 22.76 | 22.63 | 27.77 | 30.27 | 29.15 | 23.92 | 21.95 | 23.02 | 29.11 | 29.53 | 29.18 | 21.95 | 20.67 | 21.82 |
|  | CT平均值 | 29.47 | 27.35 | 29.53 | 22.26 | 23.20 | 24.02 | 29.74 | 29.52 | 29.88 | 24.65 | 22.69 | 22.52 | 27.75 | 30.33 | 29.25 | 23.90 | 21.92 | 22.94 | 29.23 | 29.71 | 29.36 | 21.77 | 20.81 | 21.75 |
|  |  |  |  |  |  |  |  |  |  |  |  |  |  |  |  |  |  |  |  |  |  |  |  |  |  |
|  | **△CT** | 6.72 | 1.07 | 5.85 | -0.98 | -1.25 | 0.07 | 5.69 | 5.25 | 3.86 | -2.07 | -1.15 | -1.45 | 4.02 | 6.51 | 5.11 | 0.33 | -1.00 | -2.41 | 5.07 | 5.43 | 3.46 | -1.85 | -2.65 | -1.50 |
|  | **△△CT** | 0.00 | -5.66 | -0.87 | -7.71 | -7.97 | -6.66 | -1.04 | -1.47 | -2.86 | -8.79 | -7.88 | -8.18 | -2.70 | -0.21 | -1.62 | -6.40 | -7.72 | -9.13 | -1.66 | -1.29 | -3.27 | -8.57 | -9.37 | -8.22 |
|  | **2^^-△△CT^** | 1.00 | 50.48 | 1.83 | 208.94 | 251.10 | 101.00 | 2.05 | 2.77 | 7.26 | 443.19 | 235.19 | 289.10 | 6.51 | 1.16 | 3.07 | 84.37 | 210.98 | 560.81 | 3.15 | 2.44 | 9.62 | 379.79 | 663.17 | 298.08 |
|  |  |  |  |  |  |  |  |  |  |  |  |  |  |  |  |  |  |  |  |  |  |  |  |  |  |
|  | **△CT** | 6.24 | 0.83 | 5.87 | -1.07 | -1.31 | 0.09 | 5.66 | 5.27 | 3.98 | -1.92 | -1.14 | -1.69 | 4.18 | 6.16 | 5.10 | 0.23 | -1.01 | -2.48 | 5.00 | 5.42 | 3.58 | -1.96 | -2.71 | -1.50 |
|  |  | 6.87 | 1.14 | 6.02 | -1.07 | -1.43 | -0.16 | 5.48 | 5.08 | 3.88 | -2.14 | -1.24 | -1.32 | 3.84 | 6.93 | 5.21 | 0.40 | -1.01 | -2.41 | 5.26 | 5.64 | 3.52 | -1.91 | -2.45 | -1.55 |
|  |  | 7.06 | 1.22 | 5.66 | -0.81 | -1.00 | 0.26 | 5.91 | 5.41 | 3.74 | -2.15 | -1.08 | -1.34 | 4.04 | 6.45 | 5.00 | 0.35 | -0.97 | -2.33 | 4.94 | 5.25 | 3.28 | -1.66 | -2.79 | -1.43 |
|  |  |  |  |  |  |  |  |  |  |  |  |  |  |  |  |  |  |  |  |  |  |  |  |  |  |
|  | **△△CT** | -0.48 | -5.89 | -0.86 | -7.79 | -8.04 | -6.63 | -1.06 | -1.45 | -2.75 | -8.64 | -7.86 | -8.41 | -2.54 | -0.57 | -1.62 | -6.50 | -7.73 | -9.20 | -1.73 | -1.31 | -3.15 | -8.68 | -9.43 | -8.23 |
|  |  | 0.14 | -5.58 | -0.70 | -7.79 | -8.15 | -6.88 | -1.24 | -1.64 | -2.85 | -8.86 | -7.96 | -8.05 | -2.88 | 0.21 | -1.52 | -6.32 | -7.74 | -9.14 | -1.46 | -1.09 | -3.21 | -8.64 | -9.17 | -8.28 |
|  |  | 0.34 | -5.50 | -1.06 | -7.54 | -7.73 | -6.46 | -0.81 | -1.32 | -2.99 | -8.87 | -7.81 | -8.07 | -2.69 | -0.27 | -1.72 | -6.38 | -7.69 | -9.06 | -1.78 | -1.47 | -3.45 | -8.39 | -9.52 | -8.15 |
|  |  |  |  |  |  |  |  |  |  |  |  |  |  |  |  |  |  |  |  |  |  |  |  |  |  |
|  | **2^^-△△CT^** | 1.40 | 59.30 | 1.81 | 221.19 | 262.84 | 99.12 | 2.08 | 2.74 | 6.71 | 399.99 | 233.02 | 340.97 | 5.83 | 1.48 | 3.08 | 90.30 | 212.91 | 588.74 | 3.31 | 2.48 | 8.85 | 411.38 | 691.24 | 300.00 |
|  |  | 0.91 | 47.96 | 1.63 | 221.96 | 284.22 | 117.96 | 2.37 | 3.12 | 7.19 | 465.05 | 249.27 | 264.26 | 7.37 | 0.86 | 2.86 | 79.95 | 213.47 | 562.57 | 2.75 | 2.13 | 9.23 | 397.63 | 576.20 | 309.82 |
|  |  | 0.79 | 45.23 | 2.08 | 185.79 | 211.94 | 88.11 | 1.76 | 2.49 | 7.94 | 467.99 | 223.98 | 268.17 | 6.44 | 1.21 | 3.30 | 83.20 | 206.63 | 532.53 | 3.44 | 2.77 | 10.90 | 334.91 | 732.28 | 284.95 |
|  | **STD** | 0.32 | 7.46 | 0.23 | 20.67 | 37.13 | 15.10 | 0.30 | 0.32 | 0.62 | 38.44 | 12.82 | 43.20 | 0.78 | 0.31 | 0.22 | 5.29 | 3.80 | 28.13 | 0.37 | 0.32 | 1.09 | 40.77 | 80.91 | 12.52 |
|  |  | 0S1 | 0S2 | 0S3 | 0T1 | 0T2 | 0T3 | 1/4S1 | 1/4S2 | 1/4S3 | 1/4T1 | 1/4T2 | 1/4T3 | 1/2S1 | 1/2S2 | 1/2S3 | 1/2T1 | 1/2T2 | 1/2T3 | 3/4S1 | 3/4S2 | 3/4S3 | 3/4T1 | 3/4T2 | 3/4T3 |
| RPL4 | CT1 | 22.76 | 26.20 | 23.69 | 23.23 | 24.51 | 23.96 | 24.01 | 24.27 | 25.95 | 26.88 | 23.88 | 23.96 | 23.86 | 23.92 | 24.42 | 23.49 | 23.00 | 25.22 | 24.13 | 24.17 | 25.96 | 23.76 | 23.42 | 23.25 |
|  | CT2 | 22.75 | 26.54 | 23.65 | 23.29 | 24.48 | 23.93 | 23.95 | 24.11 | 25.94 | 26.81 | 23.83 | 24.09 | 23.66 | 23.56 | 24.07 | 23.67 | 22.90 | 25.62 | 24.25 | 24.28 | 25.79 | 23.57 | 23.41 | 23.30 |
|  | CT3 | 22.73 | 26.12 | 23.69 | 23.21 | 24.35 | 23.98 | 24.21 | 24.42 | 26.16 | 26.45 | 23.83 | 23.86 | 23.68 | 23.96 | 23.95 | 23.56 | 22.85 | 25.20 | 24.12 | 24.38 | 25.96 | 23.51 | 23.54 | 23.19 |
|  | CT平均值 | 22.75 | 26.28 | 23.68 | 23.24 | 24.45 | 23.96 | 24.06 | 24.27 | 26.02 | 26.71 | 23.85 | 23.97 | 23.73 | 23.81 | 24.15 | 23.58 | 22.92 | 25.35 | 24.17 | 24.28 | 25.90 | 23.61 | 23.46 | 23.25 |
|  |  |  |  |  |  |  |  |  |  |  |  |  |  |  |  |  |  |  |  |  |  |  |  |  |  |
| AMY | CT1 | 30.76 | 30.92 | 31.91 | 31.11 | 26.71 | 30.79 | 30.82 | 30.34 | 31.35 | 31.30 | 29.30 | 25.81 | 29.47 | 30.50 | 29.97 | 30.07 | 27.30 | 27.95 | 30.85 | 29.38 | 29.10 | 27.28 | 29.79 | 27.30 |
|  | CT2 | 30.33 | 30.94 | 31.90 | 30.70 | 26.84 | 31.13 | 30.18 | 30.49 | 30.70 | 31.10 | 29.74 | 25.84 | 29.60 | 30.95 | 29.87 | 29.86 | 26.91 | 27.86 | 31.13 | 29.71 | 28.92 | 27.22 | 29.53 | 26.99 |
|  | CT3 | 30.52 | 31.05 | 31.90 | 30.84 | 26.56 | 30.76 | 30.22 | 30.34 | 31.27 | 31.04 | 29.43 | 25.82 | 29.60 | 31.08 | 29.78 | 29.90 | 26.97 | 27.98 | 31.55 | 29.65 | 28.62 | 27.01 | 29.80 | 27.13 |
|  | CT平均值 | 30.53 | 30.97 | 31.90 | 30.88 | 26.70 | 30.89 | 30.41 | 30.39 | 31.11 | 31.15 | 29.49 | 25.82 | 29.56 | 30.84 | 29.87 | 29.94 | 27.06 | 27.93 | 31.18 | 29.58 | 28.88 | 27.17 | 29.71 | 27.14 |
|  |  |  |  |  |  |  |  |  |  |  |  |  |  |  |  |  |  |  |  |  |  |  |  |  |  |
|  | **△CT** | 7.79 | 4.69 | 8.22 | 7.64 | 2.25 | 6.93 | 6.35 | 6.12 | 5.09 | 4.44 | 5.64 | 1.85 | 5.83 | 7.03 | 5.73 | 6.37 | 4.14 | 2.58 | 7.01 | 5.30 | 2.98 | 3.56 | 6.25 | 3.89 |
|  | **△△CT** | 0.00 | -3.10 | 0.44 | -0.15 | -5.53 | -0.85 | -1.44 | -1.67 | -2.70 | -3.35 | -2.15 | -5.93 | -1.96 | -0.76 | -2.06 | -1.42 | -3.65 | -5.20 | -0.78 | -2.49 | -4.81 | -4.23 | -1.54 | -3.89 |
|  | **2^^-△△CT^** | 1.00 | 8.58 | 0.74 | 1.11 | 46.29 | 1.81 | 2.71 | 3.18 | 6.49 | 10.21 | 4.42 | 61.14 | 3.89 | 1.69 | 4.17 | 2.67 | 12.51 | 36.88 | 1.71 | 5.60 | 28.03 | 18.77 | 2.91 | 14.88 |
|  |  |  |  |  |  |  |  |  |  |  |  |  |  |  |  |  |  |  |  |  |  |  |  |  |  |
|  | **△CT** | 8.01 | 4.63 | 8.23 | 7.87 | 2.26 | 6.83 | 6.76 | 6.07 | 5.33 | 4.59 | 5.45 | 1.84 | 5.74 | 6.69 | 5.83 | 6.49 | 4.38 | 2.60 | 6.68 | 5.10 | 3.20 | 3.67 | 6.34 | 4.05 |
|  |  | 7.58 | 4.66 | 8.22 | 7.46 | 2.39 | 7.17 | 6.12 | 6.21 | 4.69 | 4.39 | 5.89 | 1.87 | 5.87 | 7.13 | 5.73 | 6.29 | 3.99 | 2.51 | 6.96 | 5.43 | 3.02 | 3.60 | 6.07 | 3.74 |
|  |  | 7.77 | 4.77 | 8.22 | 7.59 | 2.11 | 6.80 | 6.16 | 6.07 | 5.25 | 4.33 | 5.58 | 1.85 | 5.87 | 7.26 | 5.63 | 6.33 | 4.05 | 2.64 | 7.39 | 5.37 | 2.71 | 3.40 | 6.34 | 3.89 |
|  |  |  |  |  |  |  |  |  |  |  |  |  |  |  |  |  |  |  |  |  |  |  |  |  |  |
|  | **△△CT** | 0.22 | -3.16 | 0.45 | 0.08 | -5.52 | -0.96 | -1.03 | -1.71 | -2.46 | -3.20 | -2.34 | -5.95 | -2.05 | -1.10 | -1.96 | -1.30 | -3.40 | -5.19 | -1.10 | -2.69 | -4.59 | -4.12 | -1.45 | -3.74 |
|  |  | -0.21 | -3.13 | 0.43 | -0.33 | -5.39 | -0.62 | -1.67 | -1.57 | -3.10 | -3.40 | -1.90 | -5.91 | -1.92 | -0.65 | -2.06 | -1.50 | -3.80 | -5.28 | -0.83 | -2.36 | -4.77 | -4.18 | -1.72 | -4.05 |
|  |  | -0.02 | -3.02 | 0.43 | -0.19 | -5.68 | -0.99 | -1.63 | -1.72 | -2.54 | -3.46 | -2.21 | -5.94 | -1.92 | -0.52 | -2.16 | -1.46 | -3.74 | -5.15 | -0.40 | -2.41 | -5.07 | -4.39 | -1.45 | -3.90 |
|  |  |  |  |  |  |  |  |  |  |  |  |  |  |  |  |  |  |  |  |  |  |  |  |  |  |
|  | **2^^-△△CT^** | 0.86 | 8.92 | 0.73 | 0.95 | 46.04 | 1.94 | 2.04 | 3.28 | 5.50 | 9.17 | 5.05 | 61.79 | 4.13 | 2.14 | 3.89 | 2.45 | 10.58 | 36.41 | 2.15 | 6.44 | 24.03 | 17.37 | 2.73 | 13.33 |
|  |  | 1.15 | 8.76 | 0.74 | 1.26 | 42.02 | 1.54 | 3.18 | 2.97 | 8.57 | 10.56 | 3.72 | 60.32 | 3.77 | 1.57 | 4.17 | 2.83 | 13.91 | 38.77 | 1.78 | 5.12 | 27.21 | 18.18 | 3.29 | 16.54 |
|  |  | 1.01 | 8.10 | 0.74 | 1.14 | 51.27 | 1.98 | 3.09 | 3.29 | 5.80 | 11.00 | 4.61 | 61.32 | 3.78 | 1.44 | 4.46 | 2.75 | 13.32 | 35.53 | 1.32 | 5.33 | 33.66 | 20.94 | 2.73 | 14.93 |
|  | **STD** | 0.15 | 0.44 | 0.00 | 0.16 | 4.63 | 0.25 | 0.63 | 0.18 | 1.69 | 0.95 | 0.68 | 0.75 | 0.21 | 0.37 | 0.29 | 0.20 | 1.78 | 1.68 | 0.41 | 0.71 | 4.90 | 1.87 | 0.32 | 1.61 |
|  |  | 0S1 | 0S2 | 0S3 | 0T1 | 0T2 | 0T3 | 1/4S1 | 1/4S2 | 1/4S3 | 1/4T1 | 1/4T2 | 1/4T3 | 1/2S1 | 1/2S2 | 1/2S3 | 1/2T1 | 1/2T2 | 1/2T3 | 3/4S1 | 3/4S2 | 3/4S3 | 3/4T1 | 3/4T2 | 3/4T3 |
| RPL4 | CT1 | 22.76 | 26.20 | 23.69 | 23.23 | 24.51 | 23.96 | 24.01 | 24.27 | 25.95 | 26.88 | 23.88 | 23.96 | 23.86 | 23.92 | 24.42 | 23.49 | 23.00 | 25.22 | 24.13 | 24.17 | 25.96 | 23.76 | 23.42 | 23.25 |
|  | CT2 | 22.75 | 26.54 | 23.65 | 23.29 | 24.48 | 23.93 | 23.95 | 24.11 | 25.94 | 26.81 | 23.83 | 24.09 | 23.66 | 23.56 | 24.07 | 23.67 | 22.90 | 25.62 | 24.25 | 24.28 | 25.79 | 23.57 | 23.41 | 23.30 |
|  | CT3 | 22.73 | 26.12 | 23.69 | 23.21 | 24.35 | 23.98 | 24.21 | 24.42 | 26.16 | 26.45 | 23.83 | 23.86 | 23.68 | 23.96 | 23.95 | 23.56 | 22.85 | 25.20 | 24.12 | 24.38 | 25.96 | 23.51 | 23.54 | 23.19 |
|  | CT平均值 | 22.75 | 26.28 | 23.68 | 23.24 | 24.45 | 23.96 | 24.06 | 24.27 | 26.02 | 26.71 | 23.85 | 23.97 | 23.73 | 23.81 | 24.15 | 23.58 | 22.92 | 25.35 | 24.17 | 24.28 | 25.90 | 23.61 | 23.46 | 23.25 |
|  |  |  |  |  |  |  |  |  |  |  |  |  |  |  |  |  |  |  |  |  |  |  |  |  |  |
| SUS | CT1 | 24.63 | 27.26 | 25.31 | 23.25 | 24.06 | 24.42 | 25.36 | 25.78 | 25.94 | 27.15 | 24.48 | 24.14 | 24.96 | 24.92 | 25.35 | 24.12 | 22.19 | 24.89 | 25.24 | 25.35 | 26.88 | 22.07 | 22.74 | 22.90 |
|  | CT2 | 24.50 | 27.12 | 25.07 | 23.15 | 24.07 | 24.57 | 25.40 | 25.69 | 25.96 | 27.21 | 24.85 | 24.26 | 24.55 | 24.94 | 25.52 | 23.99 | 22.23 | 24.79 | 25.79 | 25.13 | 26.75 | 22.05 | 22.73 | 22.97 |
|  | CT3 | 24.42 | 27.08 | 25.24 | 23.17 | 24.23 | 24.55 | 25.51 | 25.82 | 25.83 | 26.94 | 24.32 | 23.93 | 24.72 | 25.02 | 25.91 | 24.22 | 22.20 | 24.94 | 25.23 | 25.46 | 27.00 | 22.14 | 22.89 | 22.91 |
|  | CT平均值 | 24.51 | 27.16 | 25.21 | 23.19 | 24.12 | 24.51 | 25.43 | 25.76 | 25.91 | 27.10 | 24.55 | 24.11 | 24.74 | 24.96 | 25.59 | 24.11 | 22.21 | 24.88 | 25.42 | 25.31 | 26.88 | 22.09 | 22.79 | 22.92 |
|  |  |  |  |  |  |  |  |  |  |  |  |  |  |  |  |  |  |  |  |  |  |  |  |  |  |
|  | **△CT** | 1.77 | 0.87 | 1.53 | -0.05 | -0.33 | 0.56 | 1.37 | 1.49 | -0.10 | 0.39 | 0.70 | 0.14 | 1.01 | 1.14 | 1.45 | 0.53 | -0.71 | -0.47 | 1.25 | 1.04 | 0.97 | -1.52 | -0.67 | -0.32 |
|  | **△△CT** | 0.00 | -0.90 | -0.24 | -1.82 | -2.10 | -1.21 | -0.40 | -0.27 | -1.87 | -1.38 | -1.07 | -1.63 | -0.76 | -0.62 | -0.32 | -1.23 | -2.48 | -2.24 | -0.51 | -0.73 | -0.79 | -3.29 | -2.44 | -2.09 |
|  | **2^^-△△CT^** | 1.00 | 1.86 | 1.18 | 3.53 | 4.28 | 2.32 | 1.32 | 1.21 | 3.66 | 2.61 | 2.09 | 3.09 | 1.69 | 1.54 | 1.25 | 2.35 | 5.57 | 4.73 | 1.43 | 1.66 | 1.73 | 9.80 | 5.42 | 4.26 |
|  |  |  |  |  |  |  |  |  |  |  |  |  |  |  |  |  |  |  |  |  |  |  |  |  |  |
|  | **△CT** | 1.88 | 0.98 | 1.63 | 0.01 | -0.39 | 0.46 | 1.30 | 1.51 | -0.08 | 0.44 | 0.63 | 0.17 | 1.23 | 1.11 | 1.21 | 0.54 | -0.73 | -0.46 | 1.08 | 1.07 | 0.98 | -1.54 | -0.71 | -0.35 |
|  |  | 1.75 | 0.84 | 1.39 | -0.09 | -0.38 | 0.62 | 1.35 | 1.42 | -0.06 | 0.50 | 1.00 | 0.29 | 0.82 | 1.12 | 1.37 | 0.41 | -0.69 | -0.56 | 1.62 | 0.85 | 0.85 | -1.56 | -0.73 | -0.28 |
|  |  | 1.67 | 0.79 | 1.56 | -0.07 | -0.22 | 0.60 | 1.46 | 1.55 | -0.18 | 0.22 | 0.47 | -0.04 | 0.98 | 1.21 | 1.76 | 0.65 | -0.72 | -0.40 | 1.07 | 1.18 | 1.09 | -1.47 | -0.57 | -0.34 |
|  |  |  |  |  |  |  |  |  |  |  |  |  |  |  |  |  |  |  |  |  |  |  |  |  |  |
|  | **△△CT** | 0.11 | -0.79 | -0.13 | -1.76 | -2.16 | -1.31 | -0.47 | -0.26 | -1.84 | -1.33 | -1.14 | -1.60 | -0.54 | -0.66 | -0.56 | -1.23 | -2.50 | -2.23 | -0.69 | -0.69 | -0.79 | -3.31 | -2.48 | -2.12 |
|  |  | -0.02 | -0.93 | -0.38 | -1.86 | -2.14 | -1.15 | -0.42 | -0.34 | -1.82 | -1.27 | -0.77 | -1.48 | -0.95 | -0.65 | -0.40 | -1.36 | -2.45 | -2.32 | -0.15 | -0.92 | -0.92 | -3.33 | -2.50 | -2.05 |
|  |  | -0.10 | -0.97 | -0.20 | -1.84 | -1.99 | -1.17 | -0.31 | -0.22 | -1.95 | -1.55 | -1.30 | -1.81 | -0.78 | -0.56 | -0.01 | -1.12 | -2.49 | -2.17 | -0.70 | -0.59 | -0.67 | -3.24 | -2.34 | -2.11 |
|  |  |  |  |  |  |  |  |  |  |  |  |  |  |  |  |  |  |  |  |  |  |  |  |  |  |
|  | **2^^-△△CT^** | 0.92 | 1.73 | 1.10 | 3.39 | 4.46 | 2.48 | 1.38 | 1.20 | 3.59 | 2.52 | 2.20 | 3.03 | 1.45 | 1.58 | 1.48 | 2.34 | 5.64 | 4.68 | 1.62 | 1.62 | 1.73 | 9.92 | 5.59 | 4.34 |
|  |  | 1.01 | 1.91 | 1.30 | 3.62 | 4.42 | 2.22 | 1.34 | 1.27 | 3.54 | 2.41 | 1.70 | 2.78 | 1.93 | 1.57 | 1.32 | 2.56 | 5.48 | 5.01 | 1.11 | 1.89 | 1.89 | 10.06 | 5.66 | 4.13 |
|  |  | 1.07 | 1.96 | 1.15 | 3.58 | 3.97 | 2.25 | 1.24 | 1.16 | 3.87 | 2.92 | 2.46 | 3.51 | 1.72 | 1.47 | 1.01 | 2.17 | 5.61 | 4.51 | 1.63 | 1.50 | 1.60 | 9.43 | 5.05 | 4.31 |
|  | **STD** | 0.07 | 0.12 | 0.10 | 0.12 | 0.27 | 0.14 | 0.07 | 0.05 | 0.18 | 0.27 | 0.38 | 0.37 | 0.24 | 0.06 | 0.24 | 0.19 | 0.09 | 0.25 | 0.30 | 0.20 | 0.15 | 0.33 | 0.33 | 0.12 |
