# Supplementary material for: Comparative physiological, biochemical, metabolomic, and transcriptomic analyses reveal the formation mechanism of heartwood for Acacia melanoxylon
Source: BMC Plant Biol. 2024 Apr 22;24:308. doi: 10.1186/s12870-024-04884-1 (PMC11034122; doi:10.1186/s12870-024-04884-1)
Supplement: Supplementary file 17 — Additional file 17: Table S11. Raw data of FPKM related to the genes of RT-PCR in SR25SW vs. SR25TZ. [file 12870_2024_4884_MOESM17_ESM.docx]

**Additional file 17: Table S11.** Raw data of FPKM related to the genes of RT-PCR in SR25SW vs. SR25TZ.

| Gene family | #ID | SR25s-3_Count | SR25s-2_Count | SR25s-1_Count | SR25t-3_Count | SR25t-2_Count | SR25t-1_Count | SR25s-3_FPKM | SR25s-2_FPKM | SR25s-1_FPKM | SR25t-3_FPKM | SR25t-2_FPKM | SR25t-1_FPKM | regulated | FDR | log2FC | FC |
| --- | --- | --- | --- | --- | --- | --- | --- | --- | --- | --- | --- | --- | --- | --- | --- | --- | --- |
| HCT(hydroxycinnamoyl shikimate transferase) | evm.TU.Chr4.1122 | 0.00 | 0.00 | 0.00 | 105.00 | 111.00 | 2.00 | 0.00 | 0.00 | 0.00 | 1.62 | 1.65 | 0.03 | up | 0.00 | 8.44 | 346.98 |
| F3'H(flavanone 3'-hydroxylase) | evm.TU.Chr11.2 | 210.00 | 5430.00 | 227.00 | 189780.00 | 341046.00 | 2906.00 | 4.19 | 77.93 | 4.23 | 3001.66 | 5179.34 | 47.90 | up | 0.00 | 6.24 | 75.76 |
| CAD(cinnamyl-alcohol dehydrogenase) | evm.TU.Chr1.3453 | 2.00 | 18.00 | 4.00 | 674.00 | 1115.00 | 13.00 | 0.02 | 0.14 | 0.04 | 5.87 | 9.33 | 0.12 | up | 0.00 | 5.99 | 63.77 |
| PAL(phenylalanine ammonia-lyase) | evm.TU.Chr2.409 | 65.00 | 167.00 | 8.00 | 3410.00 | 5098.00 | 117.00 | 0.85 | 1.56 | 0.10 | 35.28 | 50.65 | 1.26 | up | 0.00 | 4.94 | 30.65 |
| AMY(alpha-amylase) [EC:3.2.1.1] | evm.TU.Chr4.2380 | 11.00 | 11.00 | 0.00 | 3710.00 | 679.00 | 52.00 | 0.19 | 0.13 | 0.00 | 51.35 | 9.01 | 0.75 | up | 0.00 | 7.43 | 172.10 |
| SUS/Susy(sucrose synthase)[EC:2.4.1.13] | evm.TU.Chr10.1547 | 158.00 | 43.00 | 12.00 | 2351.00 | 4196.00 | 71.00 | 1.11 | 0.22 | 0.08 | 13.09 | 22.44 | 0.41 | up | 0.00 | 4.80 | 27.88 |
| WRKY(transcription factors) | evm.TU.Chr2.1563 | 176.00 | 341.00 | 49.00 | 1434.00 | 2162.00 | 799.00 | 3.06 | 4.28 | 0.79 | 19.87 | 28.76 | 11.53 | up | 0.01 | 2.82 | 7.06 |
| WRKY(transcription factors) | evm.TU.Chr7.346 | 117.00 | 45.00 | 10.00 | 1784.00 | 3279.00 | 59.00 | 2.02 | 0.56 | 0.15 | 24.60 | 43.42 | 0.84 | up | 0.00 | 4.73 | 26.57 |
